# A New Approach to Staging Diabetic Eye Disease: Staging of Diabetic Retinal Neurodegeneration and Diabetic Macular Edema

**DOI:** 10.1016/j.xops.2023.100420

**Published:** 2023-10-31

**Authors:** Roomasa Channa, Risa M. Wolf, Rafael Simo, Mitchell Brigell, Patrice Fort, Christine Curcio, Stephanie Lynch, Frank Verbraak, Michael D. Abramoff, Diabetic Retinal Neurodegeneration and Macular Edema working group of the Mary Tyler Moore Vision Initiative’s Diabetic Retinal Disease Staging Update Project, Michael D. Abramoff, Roomasa Channa, Risa M. Wolf, Rafael Simo, Mitch Brigell, Patrice Fort, Christine Curcio, Stephanie Lynch, Frank Verbraak, Thomas W. Gardner

**Affiliations:** 1 Department of Ophthalmology and Visual Sciences, University of Wisconsin, Madison, Wisconsin; 2 Department of Pediatric Endocrinology, Johns Hopkins University School of Medicine, Baltimore, Maryland; 3 Division of Endocrinology, Vall d’Hebron University Hospital, CIBERDEM, Barcelona, Spain; 4 Consultant, Belmont, Massachusetts; 5 Department of Ophthalmology and Visual Sciences, University of Michigan, Ann Arbor, Michigan; 6 Department of Ophthalmology, University of Alabama, Birmingham, Alabama; 7 Georgia Eye, Athens, Georgia; 8 Department of Ophthalmology, Amsterdam University Medical Center, Amsterdam, The Netherlands; 9 Department of Ophthalmology and Visual Sciences, University of Iowa, Iowa City, Iowa

**Keywords:** Biomarkers, Diabetic retinal disease, Imaging modalities, Neuro-retinal layers

## Abstract

**Topic:**

The goal of this review was to summarize the current level of evidence on biomarkers to quantify diabetic retinal neurodegeneration (DRN) and diabetic macular edema (DME).

**Clinical relevance:**

With advances in retinal diagnostics, we have more data on patients with diabetes than ever before. However, the staging system for diabetic retinal disease is still based only on color fundus photographs and we do not have clear guidelines on how to incorporate data from the relatively newer modalities into clinical practice.

**Methods:**

In this review, we use a Delphi process with experts to identify the most promising modalities to identify DRN and DME. These included microperimetry, full-field flash electroretinogram, spectral-domain OCT, adaptive optics, and OCT angiography. We then used a previously published method of determining the evidence level to complete detailed evidence grids for each modality.

**Results:**

Our results showed that among the modalities evaluated, the level of evidence to quantify DRN and DME was highest for OCT (level 1) and lowest for adaptive optics (level 4).

**Conclusion:**

For most of the modalities evaluated, prospective studies are needed to elucidate their role in the management and outcomes of diabetic retinal diseases.

**Financial Disclosure(s):**

Proprietary or commercial disclosure may be found in the Footnotes and Disclosures at the end of this article.

**Key Points**

1. The current staging system of diabetic retinal disease is based on color fundus photographs and does not incorporate the changes seen in the retina using relatively newer modalities.
2. This review summarizes the level of evidence available in the literature regarding selected modalities to quantify diabetic retinal neurodegeneration and diabetic macular edema.
3. We found the highest level of evidence for spectral-domain OCT to quantify DRN and DME to be potentially included in an updated classification scheme for DRD.
4. Prospective studies are needed to elucidate the impact on patient outcomes from the additional information provided by the newer imaging modalities.

Diabetes (DM) is the primary cause of visual disability in the United States and around the world. In the retina, DM leads to diabetic retinal disease (DRD), which can result in vision loss and blindness if not managed appropriately.^1^ Since a key publication by Leber in 1875,^2^ research into the pathophysiology of DRD has focused on its vascular component.^3^ Consequently, the ETDRS was developed over 40 years ago, to associate specific DRD vascular phenotypes on clinical examinations and standard fundus photographs with visual outcomes (if left untreated),^4^ forming a so-called prognostic standard.^5^ Because of this tight link between ETDRS severity and outcome, it continues to be widely used as a prognostic standard and surrogate outcome for evaluations, in the diagnostic and treatment settings.^5^ In parallel, a metric for diabetic macular edema (DME), “clinically significant macular edema” was developed, based on stereo photographs of the macula, and continues to be used for the evaluation of focal and grid photocoagulation.^6^

However, the introduction of new diagnostic techniques, especially imaging modalities such as spectral-domain OCT (SD-OCT), widefield fundus imaging, adaptive optics, OCT angiography (OCTA), and electroretinogram (ERG), as well as relatively new treatment modalities, such as antivascular endothelial growth factor injections, has made it clear that many phenotypic aspects of DRD are not adequately captured in ETDRS/clinically significant macular edema scales.^7^^,^^8^ Initially, this led to the development of the concept of center-involved macular edema, based solely on macular OCT, which also became a prognostic standard.^9^^,^^10^ The rediscovery of diabetic retinal neurodegeneration (DRN) as an important factor in DRD^11^^,^^12^ and the fact that DRN may occur in the absence of vascular changes in the retina^3^ made it clear that an expansion of DRD staging to include additional metrics independent of vascular changes^13^ is required. In fact, the American Diabetes Association has recently defined diabetic retinopathy as a highly specific neurovascular disease.^14^ An ever-expanding number of disparate metrics on top of ETDRS, without an underlying pathophysiological framework, will make it increasingly unwieldy to associate phenotype with the outcome especially when clinical usage and trial end points are considered.

The purpose of this paper is to offer a systematic review of current and emerging biomarkers to quantify DRD, related to DRN and DME. As mentioned, DRN, an early component of DRD,^9^ can precede any other manifestation of DRD, including any vascular manifestations,^11^^,^^15^ and may also affect future ischemic and exudative (DME) forms of DRD. Although the relationship between DRN and visual outcome is poorly understood, there is mounting evidence indicating that the impairment of the neurovascular unit plays a key role in vascular leakage, a critical feature of the early stages of DRD.^7^^,^^9^ Thus, metrics that measure aspects of neurovascular unit dysfunction should be prioritized. To illustrate, whether DME is a factor in the development or acceleration of DRN, whether DRN instead is a factor in the development of DME, or whether they operate independently of each other is yet unknown.^8^ Thus, it is entirely possible that either metrics for one of them also measures the other, but also, that we will continue to require independent measures for both DRN and DME.

## Methods

In this systematic review, we performed a deliberate review process for each set of biomarkers. Our definition of a biomarker is based on the definition by the National Institutes of Health and United States Food and Drug Administration^16^: a biomarker is a “defined characteristic that is measured as an indicator of normal biological processes, or pathogenic processes.” For example, capillary occlusion is a biomarker, and this biomarker can be estimated using various modalities (i.e., the technology used for estimating the biomarker): including OCTA, histology, and fundus fluorescein angiography). The numeric representation of these estimates are the biomarker’s parameters, which we have also described as “dimensions.”*^8^* In the case of capillary nonperfusion, this includes capillary density (estimated from OCTA), hypofluorescence (estimated from fundus fluorescein angiography), foveal avascular zone (FAZ) area (estimated from OCTA), and FAZ irregularity (estimated from fundus fluorescein angiography or OCTA). We determined the biomarkers and potential parameters with relevance to DRD through a Delphi process. We grouped them according to the measurement modality. Ultimately, we determined relevant sets of biomarkers for each of the following modalities: adaptive optics scanning laser ophthalmoscopy (AOSLO), microperimetry, ERG, neuroretinal OCT analysis, and SD-OCT and OCTA imaging in DME. Other parameters and biomarkers were considered but not included because a limited number of peer-reviewed publications were available or because the method was considered too experimental. The method used to determine evidence levels was based on a previously published paper by Simon et al.^17^ Table 1 lists the definitions of the levels of evidence, and Table 2 lists the elements used to make the determination regarding the level of evidence. At least 2 members of the workgroup completed an evidence grid for each biomarker (Supplemental Material, available at www.ophthalmologyscience.org/). The evidence grids provide details regarding search and pruning criteria, the number of studies included, the scientific understanding regarding the relationship of the parameter to DRD, performance expectations in DRD, and types of data for evaluating the level of evidence. We have summarized the biomarkers and parameters in Table 3. A summary of the review process for each set of biomarkers is as follows:

• At least 2 members independently performed a literature search
• Search results were pruned based on journal quality and relevance to the subject
• Non-English full text and any language case reports or case series were pruned

**Table 1:** Level of Evidence Determination∗ (Based on Elements from Table 2)

Level of Evidence	Category from Table 1	Validation Studies Available
I	A	None required
I	B	≥ 1 with consistent results
II	B	None or inconsistent results
II	C	≥ 2 with consistent results
III	C	None or 1 with consistent results or inconsistent results
IV_V	D	not applicable†

∗ Levels of Evidence (LOEs) revised from those originally proposed by Simon et al.^17^

† NA = not applicable because LOE IV and V studies will never be satisfactory for determination of medical utility.

**Table 2:** Elements for Level of Evidence Determination

Category Element	A Prospective	B Prospective Using Archived Samples	C Prospective/Observational	D Retrospective/Observational
Clinical trial	PCT designed to address tumor marker	Prospective trial not designed to address tumor marker, but design accommodates tumor marker utility. Accommodation of predictive marker requires PRCT	Prospective observational registry, treatment, and follow-up not dictated	No prospective aspect to study
Patients and patient data	Prospectively enrolled, treated, and followed in PCT	Prospectively enrolled, treated, and followed in clinical trial and, especially if a predictive utility is considered, a PRCT addressing the treatment of interest	Prospectively enrolled in registry, but treatment and follow-up standard of care	No prospective stipulation of treatment or follow-up; patient data collected by retrospective chart review
Specimen collection, processing, and archival	Specimens collected, processed, and assayed for specific marker in real-time	Specimens collected, processed, and archived prospectively using generic SOPs. Assayed after trial completion	Specimens collected, processed, and archived prospectively using generic SOPs. Assayed after trial completion	Specimens collected, processed and archived with no prospective SOPs
Statistical design and analysis	Study powered to address tumor marker question	Study powered to address therapeutic question and underpowered to address tumor marker question	Study not prospectively powered at all. Retrospective study design confounded by selection of specimens for study	Study not prospectively powered at all. Retrospective study design confounded by selection of specimens for study
		Focused analysis plan for marker question developed before doing assays	Focused analysis plan for marker question developed before doing assays	No focused analysis plan for marker question developed before doing assays
Validation	Result unlikely to be a play of chance	Result more likely to be a play of chance than A but less likely than C	Result very likely to be a play of chance	Result very likely to be a play of chance
	Although preferred, validation not required	Requires ≥ 1 validation studies	Requires subsequent validation studies	Requires subsequent validation

PCT = prospective controlled trial; PRCT = prospective randomized controlled trial; SOPs = standard operating practices.

**Table 3:** List of Biomarkers and Parameters for Measuring Diabetic Retinal Neurodegeneration and Diabetic Macular Edema

Biomarker	Parameter/Dimensions	Modality	Level of Evidence
Neuroretinal function	Retinal sensitivity	Microperimetry	3A
	Implicit time, oscillatory potentials	Full-field flash ERG	1B
Neuroretinal structure	Qualitative and quantitative evaluation of neuroretina	Spectral-domain OCT	2C
	Numerous possible parameters	Adaptive Optics Scanning Laser Ophthalmoscopy	4
Retinal structural changes associated with DME	Central subfield thickness plus additional parameters	Spectral-domain OCT	1
Retinal vascular changes associated with DME	Vessel densities, foveal avascular zone, and others	OCT angiography	2B

DME = diabetic macular edema; ERG = electroretinogram.

The results were inserted into the so-called “GRID” (see Supplementary Material).

At regular workgroup meetings every 2 to 4 weeks, ≥ 1 grids were reviewed. An iterative Delphi process was performed to complete the grid and identify:

• Evidence level
• Epistemological gaps
• What to include in DRD staging
• Using the completed grids, members created a summary narrative for each biomarker set
• Gap analysis was performed

The level of evidence was determined according to the scale in Tables 1 and 2.^17^ The grids are included as Appendices, although the narratives are presented in the results section below.

This study adhered to the tenets of the Declaration of Helsinki.

## Results

In the following subsections, we have summarized key points from each of the grids completed by the workgroup for the following biomarkers:

1) Retinal sensitivity assessed by microperimetry
2) Qualitative and quantitative evaluation of neuroretinal layers using SD-OCT
3) Neuroretinal function evaluated by full-field flash electroretinogram (ffERG)
4) Quantification of DME neurodegeneration using SD-OCT
5) Quantification of OCT-angiography metrics for DME
6) Retinal microstructure imaged by adaptive optics

Table 4 summarizes the stage of DRD for which each of the imaging modalities is likely useful and also the relative state of readiness of each of the modalities.

**Table 4:** Retinal Imaging: Diabetic Retinal Neurodegeneration and Macular Edema

DRD Stage	Ready (For Current Use or within the Next 1–2 Years)	Promising (Unmet, But Defined Research Needs That Can Be Accomplished within Next 5 Years)	Potential (Unmet Research Needs That Will Need > 5 Years to Accomplish)
Subclinical DRD (not clinically visible or evident)	SD-OCT with analytics	OCTA Full-field flash ERG Microperimetry	Adaptive optics
Early-stage clinical DRD	SD-OCT with analytics	OCTA Full-field flash ERG Microperimetry	Adaptive optics
Mild-stage clinical DRD	SD-OCT with analytics	OCTA Full-field flash ERG Microperimetry	
Late-stage clinical DRD	SD-OCT with analytics	OCTA Full-field flash ERG Microperimetry	

DRD = diabetic retinal disease; ERG = electroretinogram; OCTA = OCT angiography; SD-OCT = spectral-domain OCT.

### Retinal Sensitivity Assessed by Microperimetry

#### Description of the Parameter

Retinal sensitivity, assessed by microperimetry, is currently used to measure retinal neural dysfunction in age-related macular degeneration and retinitis pigmentosa but not in the setting of DRD. There is a lack of consensus regarding normal values, mainly due to the different devices used and the characteristics of subjects included. In eyes with age-related macular degeneration, the point-wise coefficient of repeatability of microperimetry (representing the location where 95% of the test–retest differences are expected to lie) is ≤ ±4.37 dB.^18^

#### Scientific Understanding of Relationship to DRD

The reason for the reduction of retinal sensitivity in DRD is most likely due to neural impairment/neural loss as has been reported in aging.^19^ The rationale to propose the use of retinal sensitivity assessed by microperimetry to evaluate DM-induced retinal neurodysfunction is based on the following considerations:

a) Retinal sensitivity assessed by microperimetry has been correlated with ganglion cell layer-inner-plexiform layer thickness and ganglion cell count in subjects with DM. A reduction of 27 μm in total retinal thickness results in approximately 1 dB of sensitivity loss in subjects with mild nonproliferative DR.^20^
b) Microperimetry is a noninvasive and rapid test (taking approximately 6–7 minutes to complete).

#### Performance Expectations in DRD

There are limited studies evaluating the association between retinal sensitivity assessed by microperimetry and the diabetic retinopathy severity scale (DRSS). In the early stages of DR, supplementation with high-dose docosahexaenoic acid plus xanthophyll carotenoid multivitamin was associated with a progressive and significant improvement of macular function measured by microperimetry (macular sensitivity increased from 25.9 ± 2.4 dB at baseline to 27.3± 2.3 dB in the docosahexaenoic acid group; *P* < 0.05).^21^ In the advanced stages of DR, retinal sensitivity from microperimetry was correlated with visual response in patients undergoing treatment with intravitreal ranibizumab for DME. In good responders, the mean intrasubject improvement in sensitivity after 3 months of treatment was 2.28 dB (*P* = 0.049), whereas, in poor responders, it was 1.07 dB (*P* = 0.28).^22^

#### Statistical Considerations

Several variables should be considered when interpreting output from microperimetry. These include age, presence of macular edema, area of capillary nonperfusion, cognitive impairment, presence of cataracts, degrees of the macular area analyzed, number of stimulus points, type of device, and mesopic adaptation.

#### Gap Analysis

Creation of a normative database stratified by age, sex, and ethnicity. A large prospective study is ongoing.^23^ In this study, the correlation between retinal sensitivity measured by microperimetry (MAIA) and other parameters of DRD will be evaluated in subjects with type 2 DM.

#### Level of Evidence

3a.

### Qualitative and Quantitative Evaluation of the Neuroretinal Layers Using SD-OCT

#### Description of the Parameter

Spectral domain OCT can be used for qualitative and quantitative evaluation of the neuroretinal layers including macular retinal nerve fiber layer thickness (mRNFL), macular ganglion cell layer, macular ganglion-cell inner plexiform layer, peripapillary retinal nerve fiber layer, and macular ganglion cell complex: sum of the mRNFL and macular ganglion-cell inner plexiform layer. We did not consider swept-source OCT as a separate means of evaluating the parameter because of its limited availability and limited studies on the topic. A recent meta-analysis evaluating thickness measurements of these retinal layers using SD-OCT concluded that ganglion cell-inner plexiform layer **(**GC-IPL), ganglion cell complex, and mRNFL were significantly thinner among patients with DM but no retinopathy (NDR) compared with healthy controls (n = 1204 NDR vs. 1013 controls for GC-IPL analysis; n = 390 NDR vs. 416 controls for RNFL analysis; and n = 636 for NDR vs. 504 controls for ganglion cell complex analysis).^24^ One of the largest cross-sectional studies to date on this topic, including 5433 eyes with DM and no/mild DRD and 123 868 eyes in subjects without DM, concluded that GC-IPL thickness was significantly lower in the group with DM and no/mild DRD compared with no DM but that there was no difference in mRNFL thickness.^25^ GC-IPL thickness may be a more robust and earlier marker of retinal neurodegenerative changes in DM compared with mRNFL thickness.

For GC-IPL thickness, the proprietary software uses a private normative data set to flag those with thinning. At this point, normative values are not as well established for the thickness of individual retinal layers as they are for total retinal thickness. Therefore, for an individual patient, a change in thickness over time may be more clinically relevant than thickness measurement at a given point in time. Among others, age, refractive error, axial length, sex, ethnicity, glaucoma, and genetic factors have been reported to impact GC-IPL thickness.^26^^,^^27^ Other confounders, such as total retinal thickness, may also exist and need to be studied in people with DM.^28^ Additional data are needed to identify the main factors affecting inner retinal thickness.

For qualitative assessment of inner retinal layers, evaluating SD-OCT scans for the presence and extent of disorganization of inner retinal layers (DRIL) is reported to be a reliable and reproducible metric.^29^ In cross-sectional studies, DRIL has also been associated with severity of DRD, ellipsoid zone disruption, reduced visual acuity (VA), reduced contrast sensitivity, reduced performance on standard automated perimetry, and inner retinal and RNFL thinning.^30^^,^^31^

#### Scientific Understanding of the Relationship to DRD

There is no established association between DRN, as assessed by SD-OCT, and DRSS. There are few prospective studies evaluating the relationship of this parameter to outcomes in DRDs, such as vision loss, response to treatment, or DRD progression. A prospective cohort study showed that in patients with DM, quadrants of the retina with increased GCL thinning were more likely to develop DRD changes over the course of 6 years of follow-up.^32^ A retrospective cohort study showed that lower mGCIPL thickness at baseline and a higher rate of decline in mGCIPL thickness were associated with the progression of DR.^33^ Investigation of donor eyes has demonstrated damage to neuronal tissue concurrent with vascular changes, and other studies have shown thinner RNFL in donor eyes with DM compared with age-matched control eyes.^15^^,^^34^ Preclinical studies (summarized in the grid) shed important insights into the pathophysiological basis of DRN and the impact of DRN on this parameter. Many interventions have been shown to decrease neurodegeneration in animal models of DM, but there are limited animal studies on the impact of interventions on inner retinal thickness measurements using OCT.^35^ A lack of nonhuman primate models limits progress; these have so far been unable to reliably model retinal neovascularization or neuronal degeneration.^36^

#### Performance Expectations in DRD

There is currently no standardized method to clinically evaluate DRN. Longitudinal studies report thinning of mRNFL and GC-IPL over time in patients with type 1 and type 2 DM.^15^^,^^33^^,^^37^, ^38^, ^39^, ^40^ Studies show that these changes occur before the development of DRD as assessed using fundus photos, clinical examination, and histological examination.^15^^,^^33^ More recent studies have also evaluated DRD changes using OCTA.^38^^,^^41^^,^^42^ These studies have evaluated changes over time and reported that measures of microvascular damage (i.e., hemorrhages, vessel density, or FAZ metrics) and neurodegeneration (i.e., GCL-and-IPL thinning) progressed over a 2 to 4 year follow-up period. Another study, using FA to identify early DRD changes, compared the rate of GC-IPL thinning in healthy controls, eyes with DM (no DR), and DM (mild-to-moderate NPDR). This prospective study followed patients over 3 years and reported significantly higher rates of GC-IPL thinning in the groups with DM (NPDR) and DM (no DR), compared with healthy controls. The reported annual rates of GC-IPL thinning were −0.277 μm per year, −0.627 μm per year, and −0.987 μm per year in healthy controls, DM (no DR), and DM (NPDR) groups respectively. A previous study that followed 45 patients with DM (no or minimal DR) over a 4-year period reported annual GC-IPL thinning of 0.29 μm per year on time-domain OCT. The difference in OCT device used might account for the difference in actual numbers, but both independently conducted prospective studies showed progressive thinning of GC-IPL over multiple years.^43^

No interventions have been definitively shown to decrease the progressive thinning of inner retinal layers in humans. Results from the EUROCONDOR trials suggested that topical brimonidine and somatostatin prevented the progression of neurodysfunction (not structural changes) among a subgroup of patients who had neurodysfunction at baseline.^44^ A retrospective study showed that GC-IPL and RNFL thinning in humans may be slowed by intravitreal steroids.^45^

#### Statistical Considerations

When including this parameter in clinical studies, factors known to influence this parameter including age, gender, lens opacities, optic nerve disorders, cognitive impairment, glaucoma, ethnicity, axial length, and refractive error, should be considered or corrected for.^46^^,^^47^ However, these factors may not fully explain the variation in RNFL and GC-IPL thickness. A large study exploring various factors associated with RNFL and GC-IPL reported that their model explained only 6.7% and 11.2 % of the variation in RNFL and GC-IPL thickness, respectively.^48^ Total retinal thickness has been shown to be strongly associated with mRNFL and GC-IPL thickness and accounting for total retinal thickness, or considering the ratio of layer thickness and total retinal thickness may be important when comparing this parameter between and within patients with DM.^28^ Additionally, multilevel analyses may be needed to address correlation within eyes and between eyes from the same person.

#### Gap Analysis

More clarity on the factors contributing to the variation in RNFL and GC-IPL thickness among patients with DM is needed. Well-designed prospective studies are needed to evaluate the impact of qualitative and quantitative changes in inner retinal layers on DRD outcomes and identify those at the highest risk of progressive retinal neurodegeneration. We also need interventional studies to evaluate whether the prevention of progressive neuroretinal thinning or disruption in DM can improve DRD outcomes.

#### Level of Evidence

2C.

### Full-Field Flash Electroretinogram

#### Description of the Parameter

The parameters most relevant for studying the effect of DRD on the function of neurons of the inner retina are the b-wave implicit time (IT), 30 Hz flicker IT, oscillatory potential amplitude, and IT from the standard ffERG.^49^ We have not included the multifocal ERG or pattern ERG due to their limited availability, poor statistical parameters (test–retest reliability and signal-to-noise properties), and testing burden (duration of test and fixation requirements). We have also not included the photopic negative response because of the limited number of studies available and the lack of standardization on acquisition parameters. There is a large body of literature on cross-sectional studies showing a correlation between the selected ffERG parameters (i.e., b-wave implicit time [IT], 30 Hz flicker IT, and oscillatory potentials [OPs] IT) and DRD severity or structural measures on the effect of DRD on the retina. There have been a number of cross-sectional studies linking ETDRS DRSS level and ERG parameters in patients with DR.^50^^,^^51^ We will focus on studies evaluating the prognostic value of these parameters and their use in clinical trials.

The ITs of selected ffERG parameters have the requisite statistical parameters for use in the staging of DRD severity and are sensitive to changes in DRD severity. Full-field flash electroretinogram amplitude measurements have much higher intrasubject and intersubject variability and are not well suited for DRD staging.^52^

Finally, cone-mediated responses have proven to be sensitive to DRD severity, eliminating the need for dark adaptation before testing. There is some evidence that, in the earliest stages of DRD and in people with DM without clinical evidence of DR, rod system responses may be more sensitive to early neuronal dysfunction.^53^ In this case, recent evidence shows that 10 minutes of dark adaptation is sufficient to reliably assess the rod pathway.^54^

#### Scientific Understanding of the Relationship to DRD

Multiple studies have shown ERG evidence of neuronal dysfunction in subjects without clinical evidence of retinopathy.^55^^,^^56^ The onset of ERG changes before vascular changes has also been observed in animal models of DM.^57^ Several studies have assessed the prognostic value of ffERG. Brigell et al^58^ showed that an increase in ffERG flicker IT was associated with an increased risk of the need for intervention for DR/DME over the subsequent 1 to 3 years, independent of DRSS. Subjects with all stages of DRD were assessed with flicker ERG and 7-field color fundus photos at baseline, and the outcome (intervention for DME or PDR) was retrospectively assessed over a 3-year follow-up period.^58^ For patients with structural evidence of vision-threatening DR at baseline, the incidence of intervention was 19%, 31%, and 53% at 1, 2, and 3 years of follow-up, respectively. In these patients, intervention incidence increased to 34%, 54%, and 74% in the subsequent 1, 2, and 3 years, respectively, in eyes with increased ERG flicker IT, whereas if flicker IT was below criterion IT, the risk was reduced to 3%, 4%, and 29%, respectively, reducing risk to similar levels seen for patients without vision-threatening DR at baseline. More recently these results were validated in a prospective RCT in which patients with moderate to severe NPDR without center-involved DME were at significantly higher risk of progressing to DME or PDR over the 1-year course of the trial regardless of treatment group if their flicker ERG IT was prolonged at baseline.^59^

#### Performance Expectations in DRD

The ffERG parameters have been shown to become increasingly abnormal as DRD progresses. As with structural measurements, the decline in ffERG parameters can be reduced or stabilized with improved glycemic control.^60^

The ffERG has been used as an outcome measure in randomized controlled clinical trials for DR.^61^^,^^62^ Each of these studies had at least 1 shortcoming including small sample size, a short duration of therapy, and failure to measure more standard structural and functional outcome measures.

Recent technological advances have made the ffERG much more amenable for widespread use in screening/staging of disease and in multicenter clinical trials. Handheld Ganzfeld devices are now widely available at a lower price than conventional ERG systems. Skin electrodes have been validated against more conventional contact lens electrodes. Some newer systems adjust flash intensity based on pupil diameter to obtain a consistent flash retinal illuminance without the need for dilation of the pupil.

#### Types of Data Available for Evidential Evaluation

There are multiple in vivo studies that have evaluated this parameter in animal models of DM. These are summarized in the grid

#### Statistical Considerations

For longitudinal measures of IT, an increase of ≥ 5 ms is clinically significant for the dark-adapted 3.0 a-wave, the light-adapted 3.0 a-wave, the b-wave, and the light-adapted 3.0 flicker. A 10 ms criterion is needed for the dark-adapted 0.01 b-wave and the dark-adapted 3.0 b-wave IT.^63^

Thresholds for abnormality are generally determined by age-adjusted 95% confidence interval.^64^ Normative data are available on most commercial ERG devices. Factors other than disease state, including age and blood glucose levels, can influence the parameter.

#### Gap Analysis

It is unclear which ERG parameter is most sensitive to disease progression, and this may vary with the stage of the disease. A longitudinal observational study in patients stratified by DR severity comparing photopic (light-adapted) and scotopic (dark-adapted) b-wave and OP parameters, along with other functional and structural measures, would help clarify the role of the ffERG in DR staging.

#### Level of Evidence

1B.

### Quantitative and Qualitative Evaluation of SD-OCT for DME Neurodegeneration

#### Description of the Parameter

Macular OCT is used for the measurement of the severity and progression of macular edema, specifically center-involving DME. Center-involving DME has been well-documented as a predictor of decreased VA in patients with DM. However, the correlation is moderate. There are likely other factors, such as retinal neural integrity, that influence visual function. The advantages of macular OCT-based metrics, as a parameter, are that they can be measured in a standardized fashion, are noninvasive, and the equipment is relatively ubiquitous in the ophthalmology clinical setting. Multiple modalities can be used to evaluate the macula in the context of DME: slit-lamp examination with or without macular contact lens, fluorescein angiography, SD-OCT, and OCT angiography. This review is limited to SD-OCT. The following parameters can be quantified or qualitatively assessed using SD-OCT:

• Central point or foveal thickness
• Central subfield macular thickness (CMT) in a circular area of diameter 1 mm centered at the fovea
• Macular volume
• Presence or absence of serous retinal detachment
• Intensity of intraretinal hyperreflective dots
• Number or size of intraretinal cysts
• Polarization of the OCT signal
• Vitreoretinal integrity: vitreoretinal alterations
• Inner retinal integrity: DRIL
• Outer retina integrity: Inner outer segment band or ellipsoid zone disruption
• Outer retina integrity: retinal pigment epthelium band
• Morphologic pattern of DME (diffuse thickening, cystoid, subretinal fluid, etc.)

#### Scientific Understanding of the Relationship to DRD

The correlation of best-corrected VA with each of the parameters listed above has been widely studied, and only a modest correlation with central foveal thickness exists. The most extensive study found that, on average, baseline VA was 4.4 (95% confidence interval: 3.5, 5.3) letters better for every 100 microns decrease in center point thickness.^65^ Other studies have shown similar correlation coefficients of 0.574,^66^ 0.558,^67^ and 0.56.^68^ It is not known whether stratifying these data by patient sex and/or specific thicknesses of individual retinal layers would increase the correlation with VA. There is a significant but slightly weaker relationship between change in OCT center point thickness and change in VA over a 3.5-month study period after focal laser treatment.^65^ Other measures, such as extent of DRIL and presence/absence of DRIL, have been associated with VA.^69^, ^70^, ^71^ Multiple studies have shown that, in subjects with longstanding DME, measurements of outer retina integrity and vitreoretinal integrity are associated with good VA, after treatment,^72^, ^73^, ^74^ but larger studies have not replicated this finding.^75^

#### Performance Expectations in DRD

There is a dose-response relationship between treatment level and changes in VA and CMT. This relationship is not specific to DRD and is consistent temporally as well as across distinct types of intervention.

#### Statistical Considerations

The correlation between change in VA and CMT is approximately 0.5. Covariates include age, sex, refractive error, time of day of imaging, presence of macular ischemia, and integrity of the inner, outer retina, and vitreoretinal interface.

#### Gap Analysis

The literature does not contain a multivariate equation that correlates OCT-based thickness with VA, although accounting for other factors that independently influence visual potential. Furthermore, it is not clear if eyes that receive repeated antivascular endothelial growth factor treatments have an increased risk of ocular hypertension, glaucoma, and/or RNFL thinning, all of which could influence retinal thickness measurements, and the literature on this topic is mixed (see Grid). Further investigation requires a longitudinal, prospective observational cohort study in eyes with/without DME and with/without DR to evaluate the relative strength of the following factors as predictive of VA changes: central foveal thickness, central macular thickness, macular volume, presence of DRIL, IS/OS integrity, thickness of each retinal layer. There are many existing OCT and VA data sets from longitudinal pharmacologic studies of DME. These could be further analyzed in a retrospective manner to determine if SD-OCT features other than CMT are more predictive of outcomes.

In the future, it is possible that SD-OCT will have the capability to automatically detect and quantify macular parameters including the integrity of retinal structures using artificial intelligence.

#### Level of Evidence

I.

### Quantification of OCTA Metrics for DME

#### Description of the Parameter

OCT angiography is a noninvasive imaging modality that measures changes in FAZ and vessel density and can identify vascular changes including microaneurysms, intraretinal microvascular abnormalities, neovascularization, and vascular loops. In this section, we will review the role of OCTA in identifying and quantifying retinal vascular changes in patients with DME. A majority of the studies evaluating the robustness, repeatability, and reproducibility of OCTA metrics have been done either in healthy eyes or eyes with DRD but no significant edema.^76^, ^77^, ^78^

With improved technology, artifacts associated with OCTA have decreased, but the presence of DME makes OCTA measurements less reliable. These artifacts can be because of segmentation errors,^79^ compression of vessels from cystic spaces leading to falsely decreased perfusion as the flow decreases below the detection limits of OCTA,^80^ or extravascular fluid in cysts may have suspended scattered particles in motion, generating a spurious OCT flow signal.^81^ A study comparing 20 eyes with DME with 24 healthy eyes reported measurement errors in vessel density in all eyes with DME, compared with about a third of healthy eyes.^82^ Studies have suggested that 3D OCTA-derived metrics of perfusion may have better repeatability metrics. In 20 eyes with DR and DME, ICC was 0.6 to 0.8 for 2D and 0.93 to 0.97 for 3D OCT metrics, and the coefficient of variation ranged from 2.2 to 4.2 for 2D and 1.9 to 2.0 for 3D.^83^ Although OCTA-derived metrics may be less useful once edema has developed, they may be helpful in predicting the development of DME as described below.

#### Scientific Understanding of the Relationship to DRD

OCT angiography evaluates microvasculature, and it is well established that this is affected in eyes with DME. Two prospective studies have demonstrated the association of OCTA metrics with the development of DME. In a study looking at a subset of patients enrolled in the TIME2b clinical trial (patients with moderate to severe DRD were treated with AKB-9778 over 1 year), macular and peripapillary data were obtained from 1 of 3 commercially available OCTA devices: Zeiss Cirrus, Angiovue, or Topcon swept-source OCT, with images graded by a reading center. In this study, pretreatment larger FAZ area and the presence of intraretinal microvascular abnormalities were associated with DME development during the course of the study.^84^ In another prospective study of 205 eyes (129 patients with DM) using the Topcon swept-source OCTA, vessel density of the superficial capillary plexus predicted DME development.^85^

Studies have reported various OCTA metrics that may be predictive of VA outcome or treatment response in patients with DME (see Grid), but these findings must be interpreted with caution, given the high occurrence of artifacts in OCTA images of patients with DME.

#### Performance Expectations in DRD

Two prospective studies, described above, have shown an association between OCTA metrics and the development of DME. Both identified different OCTA metrics: in one study, larger FAZ and the presence of intraretinal microvascular abnormalities and, in the other study, vessel density of superficial capillary plexus predicted the development of DME.

#### Statistical Considerations

Output needs adjustment for multiple statistical comparisons and covariates such as age, gender, ethnicity, scan quality/signal strength, axial length, and retinal thickness.^80^^,^^86^

#### Gap Analysis

OCT angiography output gives many parameters. Prospective studies need to evaluate which parameters and scanning protocols are the most predictive of clinical outcomes. The availability of retinal thickness measures together with vascular measures gives an opportunity to study early neurodegenerative and vascular changes simultaneously.

#### Level of Evidence

2B.

### Adaptive Optics Imaging of Retinal Microstructure

#### Description of the Parameter

Adaptive optics scanning laser ophthalmoscopy imaging is a noninvasive imaging technology with the capability to assess multiple elements of the retinal microstructure. A noninvasive imaging technique, AOSLO captures en face, black-and-white images of the retina with a resolution of approximately 2 microns.^87^^,^^88^ It is uniquely capable of imaging individual retinal cells in vivo, including vascular changes and photoreceptor degeneration. Although most studies of AOSLO focus on normal eyes and inherited retinal degenerations, we evaluate a small number of studies demonstrating AOSLO’s potential to identify microscopic changes in DR.

The following parameters have been assessed from AOSLO images:

• photoreceptor density and spacing
• microaneurysm density, diameter, perfusion status, intraluminal hyperreflectivity, and/or wall hyperreflectivity
• capillary diameter
• capillary density
• vascular tortuosity
• FAZ size and shape
• blood flow velocity
• individual retinal ganglion cells

#### Scientific Understanding of the Relationship to DRD

In subjects with DM, eyes with DR have an average capillary diameter of 8.2 μm (SD 1.1 μm), although normal eyes have an average diameter of 6.1 μm (SD 0.75 μm) (*P* < 0.01).^89^ With regard to capillary density, Tam et al^90^ found no difference between patients with type 2 DM without DR compared with controls. Therefore, it has been challenging to establish a specific threshold for pathologic alterations in capillary density.^91^

#### Performance Expectations in DRD

Multiple limitations currently prevent AOSLO from widespread use including the time-intensive nature of image acquisition and analysis, limited availability of devices, and lack of standardization and normal data sets. Adaptive optics scanning laser ophthalmoscopy is exclusively used in research settings at present.

#### Statistical Considerations

The quantitative relationship between retinal structural changes and visual outcomes has not yet been characterized.

#### Gap Analysis

If AOSLO were to become a more widespread or commercially viable technology, it would be important for a standardized device and analysis software to be available, as well as access to normal data sets. Additionally, DME is a confounder leading to suboptimal images.

#### Level of Evidence

Level 4.

## Discussion

The ETDRS classification has been the standard for decades, but it is now widely recognized in the field of DRD that the ETDRS classification, based solely on microvascular structural lesions, by definition, fails to express a substantial fraction of the retinal damage relevant to DRD. On the other hand, given that many of our protocols, standards of care, diagnostics, and therapeutics, as well as estimates of clinical outcomes were developed based on the ETDRS system, it is important that the new classification scheme retain continuity. An advantage of this approach would be continuity from a system that is currently in worldwide use, although avoiding ethically questionable natural history studies, in which patients are left untreated to determine how different disease severity classes result in various outcomes.^5^

This study, and the others resulting from the Mary Tyler Moore Vision Initiative’s DRD Staging Update Project, are the first steps toward a new, comprehensive and clinically useful classification system to understand the temporal and spatial relationships between existing and new parameters and biomarkers for DRD, as we have proposed earlier.^8^ Revising the DRD classification scheme has the potential to impact clinical outcomes for millions of individuals with DM. However, at present there is a dearth of data to fully understand the impact of incorporating these newer imaging modalities and updating the DRD classification on clinical outcomes. Clearly, a substantial, longitudinal, prospective observational cohort study in eyes, with and without DR, DME, and DRN, in which eyes are evaluated with the following modalities: VA, perimetry, fundus photographs, SD-OCT, OCTA, ERG, and microperimetry, allows for the questions posed in this study to be addressed and the impact on patient outcome to be evaluated. Specifically, the studies would be designed to evaluate the predictive power of OCTA-derived FAZ regularity and capillary density, OCT-derived central macular thickness, macular volume, presence of DRIL, IS/OS integrity, the thickness of each retinal layer, ERG-derived flicker, b-wave, and OP IT, microperimetry derived retinal sensitivity, on DRSS and visual outcomes.

In our earlier proposal,^7^ we had mentioned the importance of evaluating these parameters independently, essentially treating them as independent axes on which DRD can be evaluated. Although it can be expected that multiple parameters may be found to be correlated (i.e., predict each other), ultimately only 1 predictive parameter is needed, likely the more patient-friendly, lower resource parameter. However, it may be too early to perform such a “dimensional collapse,” because the data needed to elucidate the role of these modalities in the management of DRD are still limited. Prospective studies are needed to elucidate the role of these modalities in the management of DRD and the impact of identifying these retinal changes on patient outcomes.^8^

## References

[1] Preferred Practice Patterns: diabetic retinopathy. San Francisco, CA. http://www.aao.org/preferred-practice-pattern/diabetic-retinopathy-ppp-updated-2016. Accessed August 4, 2022.

[2] Leber T.K. (1875). Über die Erkrankungen des Auges bei diabetes mellitus. Graefe’s Arch.

[3] Lynch S.K., Abràmoff M.D. (2017). Diabetic retinopathy is a neurodegenerative disorder. Vision Res.

[4] (1991). Fundus photographic risk factors for progression of diabetic retinopathy. ETDRS Report No. 12. Early Treatment Diabetic Retinopathy Study Research Group. Ophthalmology.

[5] Abràmoff M.D., Cunningham B., Patel B. (2022). Foundational considerations for artificial intelligence using ophthalmic images. Ophthalmology.

[6] (1995). Focal photocoagulation treatment of diabetic macular edema. Relationship of treatment effect to fluorescein angiographic and other retinal characteristics at baseline: ETDRS Report No. 19. Early Treatment Diabetic Retinopathy Study Research Group. Arch Ophthalmol.

[7] Sun J.K., Aiello L.P., Abràmoff M.D. (2021). Updating the staging system for diabetic retinal disease. Ophthalmology.

[8] Abramoff M.D., Fort P.E., Han I.C., Jayasundera K.T., Sohn E.H., Gardner T.W. (2018). Approach for a clinically useful comprehensive classification of vascular and neural aspects of diabetic retinal disease. Invest Ophthalmol Vis Sci.

[9] Browning D.J., Glassman A.R., Aiello L.P. (2008). Optical coherence tomography measurements and analysis methods in optical coherence tomography studies of diabetic macular edema. Ophthalmology.

[10] Glassman A.R., Beck R.W., Browning D.J., Danis R.P., Kollman C. (2009). Diabetic Retinopathy Clinical Research Network Study Group. Comparison of optical coherence tomography in diabetic macular edema, with and without reading center manual grading from a clinical trials perspective. Invest Ophthalmol Vis Sci.

[11] Simó R., Stitt A.W., Gardner T.W. (2018). Neurodegeneration in diabetic retinopathy: does it really matter?. Diabetologia.

[12] Simó R., Hernández C. (2014). European Consortium for the Early Treatment of Diabetic Retinopathy (EUROCONDOR). Neurodegeneration in the diabetic eye: new insights and therapeutic perspectives. Trends Endocrinol Metab.

[13] Wilkinson C.P., Ferris F.L., Klein R.E. (2003). Proposed international clinical diabetic retinopathy and diabetic macular edema disease severity scales. Ophthalmology.

[14] Solomon S.D., Chew E., Duh E.J. (2017). Diabetic retinopathy: A position statement by the American Diabetes Association. Diabetes Care.

[15] Sohn E.H., van Dijk H.W., Jiao C. (2016). Retinal neurodegeneration may precede microvascular changes characteristic of diabetic retinopathy in diabetes mellitus [article]. Proc Natl Acad Sci U S A.

[16] United States Food and Drug Administration (FDA) The Best Resource: Harmonizing Biomarker Terminology. https://www.fda.gov/media/99221/download.

[17] Simon R.M., Paik S., Hayes D.F. (2009). Use of archived specimens in evaluation of prognostic and predictive biomarkers. J Natl Cancer Inst.

[18] Wu Z., Ayton L.N., Guymer R.H., Luu C.D. (2013). Intrasession test-retest variability of microperimetry in age-related macular degeneration. Invest Ophthalmol Vis Sci.

[19] Johnson C.A., Adams A.J., Lewis R.A. (1989). Evidence for a neural basis of age-related visual field loss in normal observers. Invest Ophthalmol Vis Sci.

[20] Park J.C., Chen Y.F., Liu M., Liu K., McAnany J.J. (2020). Structural and functional abnormalities in early-stage diabetic retinopathy. Curr Eye Res.

[21] Rodríguez González-Herrero M.E., Ruiz M., López Román F.J., Marín Sánchez J.M., Domingo J.C. (2018). Supplementation with a highly concentrated docosahexaenoic acid plus xanthophyll carotenoid multivitamin in nonproliferative diabetic retinopathy: prospective controlled study of macular function by fundus microperimetry. Clin Ophthalmol.

[22] Palkovits S., Hirnschall N., Georgiev S., Leisser C., Findl O. (2021). Effect of cataract extraction on retinal sensitivity measurements. Ophthalmic Res.

[23] Retinal and cognitive dysfunction in type 2 diabetes: unraveling the common pathways and identification of patients at risk of dementia. https://cordis.europa.eu/project/id/847749/reporting.

[24] Tang Z., Chan M.Y., Leung W.Y. (2021). Assessment of retinal neurodegeneration with spectral-domain optical coherence tomography: a systematic review and meta-analysis. Eye (Lond).

[25] Channa R., Lee K., Staggers K.A. (2021). Detecting retinal neurodegeneration in people with diabetes: findings from the UK Biobank. PLOS ONE.

[26] Mwanza J.C., Durbin M.K., Budenz D.L. (2011). Profile and predictors of normal ganglion cell-inner plexiform layer thickness measured with frequency-domain optical coherence tomography. Invest Ophthalmol Vis Sci.

[27] Bloch E., Yonova-Doing E., Jones-Odeh E., Williams K.M., Kozareva D., Hammond C.J. (2017). Genetic and environmental factors associated with the ganglion cell complex in a healthy aging British cohort. JAMA Ophthalmol.

[28] Banghart M., Lee K., Bahrainian M. (2022). Total retinal thickness: a neglected factor in the evaluation of inner retinal thickness. BMJ Open Ophthalmol.

[29] Sampani K., Abdulaal M., Peiris T. (2020). Comparison of SDOCT scan types for grading disorganization of retinal inner layers and other morphologic features of diabetic macular edema. Transl Vis Sci Technol.

[30] Joltikov K.A., Sesi C.A., de Castro V.M. (2018). Disorganization of retinal inner layers (DRIL) and neuroretinal dysfunction in early diabetic retinopathy. Invest Ophthalmol Vis Sci.

[31] Nadri G., Saxena S., Stefanickova J. (2019). Disorganization of retinal inner layers correlates with ellipsoid zone disruption and retinal nerve fiber layer thinning in diabetic retinopathy. J Diabetes Complications.

[32] van de Kreeke J.A., Darma S., Chan Pin, Yin J.M.P.L. (2020). The spatial relation of diabetic retinal neurodegeneration with diabetic retinopathy. PLOS ONE.

[33] Kim K., Kim E.S., Yu S.Y. (2018). Longitudinal relationship between retinal diabetic neurodegeneration and progression of diabetic retinopathy in patients with Type 2 diabetes. Am J Ophthalmol.

[34] Bloodworth J.M. (1962). Diabetic retinopathy. Diabetes.

[35] Wang J., Cui X., Roon P., Smith S.B. (2016). Role of sigma 1 receptor in retinal degeneration of the Ins2Akita/+ murine model of diabetic retinopathy. Invest Ophthalmol Vis Sci.

[36] Robinson R., Barathi V.A., Chaurasia S.S., Wong T.Y., Kern T.S. (2012). Update on animal models of diabetic retinopathy: from molecular approaches to mice and higher mammals. Dis Model Mech.

[37] Pinilla I., Ferreras A., Idoipe M. (2013). Changes in frequency-doubling perimetry in patients with type I diabetes prior to retinopathy. BioMed Res Int.

[38] Kim K., Kim E.S., Kim D.G., Yu S.Y. (2019). Progressive retinal neurodegeneration and microvascular change in diabetic retinopathy: longitudinal study using OCT angiography. Acta Diabetol.

[39] Lim H.B., Shin Y.I., Lee M.W., Park G.S., Kim J.Y. (2019). Longitudinal changes in the peripapillary retinal nerve fiber layer thickness of patients with Type 2 diabetes. JAMA Ophthalmol.

[40] Khawaja A.P., Chan M.P., Garway-Heath D.F. (2013). Associations with retinal nerve fiber layer measures in the EPIC-Norfolk Eye Study. Invest Ophthalmol Vis Sci.

[41] Aschauer J., Pollreisz A., Karst S. (2022). Longitudinal analysis of microvascular perfusion and neurodegenerative changes in early type 2 diabetic retinal disease. Br J Ophthalmol.

[42] Bontzos G., Kabanarou S.A., Gkizis I., Ragkousis A., Xirou T., Peto T. (2021). Retinal neurodegeneration, macular circulation and morphology of the foveal avascular zone in diabetic patients: quantitative cross-sectional study using OCT-A. Acta Ophthalmol.

[43] Lim H.B., Shin Y.I., Lee M.W., Koo H., Lee W.H., Kim J.Y. (2020). Ganglion cell – inner plexiform layer damage in diabetic patients: 3-year prospective, longitudinal, observational study. Sci Rep.

[44] Simó R., Hernández C. (2012). European Consortium for the Early Treatment of Diabetic Retinopathy (EUROCONDOR). Neurodegeneration is an early event in diabetic retinopathy: therapeutic implications. Br J Ophthalmol.

[45] Lynch S.K., Lee K., Chen Z. (2019). Intravitreal fluocinolone acetonide may decelerate diabetic retinal neurodegeneration. Invest Ophthalmol Vis Sci.

[46] Darma S., Kok P.H., van den Berg T.J. (2015). Optical density filters modeling media opacities cause decreased SD-OCT retinal layer thickness measurements with inter- and intra-individual variation. Acta Ophthalmol.

[47] Al-Hawasi A., Lagali N. (2022). Retinal ganglion cell layer thickness and volume measured by OCT changes with age, sex, and axial length in a healthy population. BMC Ophthalmol.

[48] Khawaja A.P., Chua S., Hysi P.G. (2020). Comparison of associations with different macular inner retinal thickness parameters in a large cohort: the UK Biobank. Ophthalmology.

[49] McCulloch D.L., Marmor M.F., Brigell M.G. (2015). ISCEV Standard for full-field clinical electroretinography (2015 update). Doc Ophthalmol.

[50] Maa A.Y., Feuer W.J., Davis C.Q. (2016). A novel device for accurate and efficient testing for vision-threatening diabetic retinopathy. J Diabetes Complications.

[51] Bresnick G.H., Palta M. (1987). Oscillatory potential amplitudes. Relation to severity of diabetic retinopathy. Arch Ophthalmol.

[52] Corîci A.C., Alexandru D.O., Corîci O.M., Puianu M., Iancău M., Ştefănescu-Dima A. (2015). Variability of normal values of electroretinogram parameters due to aging in healthy individuals. Curr Health Sci J.

[53] Pardue M.T., Barnes C.S., Kim M.K. (2014). Rodent hyperglycemia-induced inner retinal deficits are mirrored in human diabetes. Transl Vis Sci Technol.

[54] Hamilton R., Graham K. (2016). Effect of shorter dark adaptation on ISCEV standard DA 0.01 and DA 3 skin ERGs in healthy adults. Doc Ophthalmol.

[55] Motz C.T., Chesler K.C., Allen R.S. (2020). Novel detection and restorative levodopa treatment for preclinical diabetic retinopathy. Diabetes.

[56] Juen S., Kieselbach G.F. (1990). Electrophysiological changes in juvenile diabetics without retinopathy. Arch Ophthalmol.

[57] Hammoum I., Benlarbi M., Dellaa A. (2018). Retinal dysfunction parallels morphologic alterations and precede clinically detectable vascular alterations in *Meriones shawi*, a model of type 2 diabetes. Exp Eye Res.

[58] Brigell M.G., Chiang B., Maa A.Y., Davis C.Q. (2020). Enhancing risk assessment in patients with diabetic retinopathy by combining measures of retinal function and structure. Transl Vis Sci Technol.

[59] Brigell M.G., Davis Q., Waheed N.K. (2020). Predictive value of ERG, OCT-A, and UWF-FA in patients with diabetic retinopathy. [ARVO Annual Meeting Abstract]. Invest Ophthalmol Vis Sci.

[60] Klefter O.N., Holfort S.K., Larsen M. (2016). Baseline haemoglobin A1c influences retinal function after long-term insulin pump therapy. Graefes Arch Clin Exp Ophthalmol.

[61] Messias K., Barroso R.M., Jorge R., Messias A. (2018). Retinal function in eyes with proliferative diabetic retinopathy treated with intravitreal ranibizumab and multispot laser panretinal photocoagulation. Doc Ophthalmol.

[62] Toscano L., Messias A., Messias K. (2021). Proliferative diabetic retinopathy treated with intravitreal ranibizumab and photocoagulation directed at ischemic retinal areas-A randomized study. Doc Ophthalmol.

[63] Grover S., Fishman G.A., Birch D.G., Locke K.G., Rosner B. (2003). Variability of full-field electroretinogram responses in subjects without diffuse photoreceptor cell disease. Ophthalmology.

[64] Birch D.G., Anderson J.L., Fish G.E., Jost B.F. (1992). Pattern-reversal electroretinographic acuity in untreated eyes with subfoveal neovascular membranes. Invest Ophthalmol Vis Sci.

[65] Virgili G., Menchini F., Casazza G. (2015). Optical coherence tomography (OCT) for detection of macular oedema in patients with diabetic retinopathy. Cochrane Database Syst Rev.

[66] Browning D.J., Glassman A.R., Diabetic Retinopathy Clinical Research Network (2007). Relationship between optical coherence tomography-measured central retinal thickness and visual acuity in diabetic macular edema. Ophthalmology.

[67] Islam F. (2016). Retinal thickness and visual acuity in diabetic macular edema: an optical coherence tomography-based study. J Coll Physicians Surg Pak.

[68] Alkuraya H., Kangave D., Abu El-Asrar A.M. (2005). The correlation between optical coherence tomographic features and severity of retinopathy, macular thickness and visual acuity in diabetic macular edema. Int Ophthalmol.

[69] Cennamo G., Montorio D., Fossataro F., Fossataro C., Tranfa F. (2021). Evaluation of vessel density in disorganization of retinal inner layers after resolved diabetic macular edema using optical coherence tomography angiography. PLOS ONE.

[70] Luís M.E., Sampaio F., Costa J., Cabral D., Teixeira C., Ferreira J.T. (2021). Dril influences short-term visual outcome after intravitreal corticosteroid injection for refractory diabetic macular edema. Curr Eye Res.

[71] Pinto M., Mathis T., Massin P. (2021). Visual acuity gain profiles and anatomical prognosis factors in patients with drug-naive diabetic macular edema treated with dexamethasone implant: the NAVEDEX study. Pharmaceutics.

[72] Borrelli E., Grosso D., Barresi C. (2022). Long-term Visual Outcomes and Morphologic Biomarkers of Vision Loss in Eyes with Diabetic Macular Edema treated with anti-VEGF Therapy. Am J Ophthalmol.

[73] Meduri A., Oliverio G.W., Trombetta L., Giordano M., Inferrera L., Trombetta C.J. (2021). Optical coherence tomography predictors of favorable functional response in naive diabetic macular edema eyes treated with dexamethasone implants as a first-line agent. J Ophthalmol.

[74] Endo H., Kase S., Tanaka H. (2021). Factors based on optical coherence tomography correlated with vision impairment in diabetic patients. Sci Rep.

[75] Etheridge T., Blodi B., Oden N. (2021). Spectral domain OCT predictors of visual acuity in the study of COmparative treatments for REtinal vein Occlusion 2: SCORE 2 Report 15. Ophthalmol Retina.

[76] Czakó C., Sándor G., Ecsedy M. (2018). Intrasession and between-visit variability of retinal vessel density values measured with OCT angiography in diabetic patients. Sci Rep.

[77] Tang F.Y., Ng D.S., Lam A. (2017). Determinants of quantitative optical coherence tomography angiography metrics in patients with diabetes. Sci Rep.

[78] Corvi F., Pellegrini M., Erba S., Cozzi M., Staurenghi G., Giani A. (2018). Reproducibility of vessel density, fractal dimension, and foveal avascular zone using 7 different optical coherence tomography angiography devices. Am J Ophthalmol.

[79] Lee J., Moon B.G., Cho A.R., Yoon Y.H. (2016). Optical coherence tomography angiography of DME and its association with anti-VEGF treatment response. Ophthalmology.

[80] Sun Z., Yang D., Tang Z., Ng D.S., Cheung C.Y. (2021). Optical coherence tomography angiography in diabetic retinopathy: an updated review. Eye (Lond).

[81] Kashani A.H., Green K.M., Kwon J. (2018). Suspended scattering particles in motion: A novel feature of OCT angiography in exudative maculopathies. Ophthalmol Retina.

[82] Ghasemi Falavarjani K., Habibi A., Anvari P. (2020). Effect of segmentation error correction on optical coherence tomography angiography measurements in healthy subjects and diabetic macular oedema. Br J Ophthalmol.

[83] Borrelli E., Parravano M., Costanzo E. (2021). Using three-dimensional optical coherence tomography angiography metrics improves repeatability on quantification of ischemia in eyes with diabetic macular edema. Retina.

[84] Custo Greig E., Brigell M., Cao F. (2020). Macular and peripapillary optical coherence tomography angiography metrics predict progression in diabetic retinopathy: a sub-analysis of TIME-2b study data. Am J Ophthalmol.

[85] Sun Z., Tang F., Wong R. (2019). OCT angiography metrics predict progression of diabetic retinopathy and development of diabetic macular edema: A prospective study. Ophthalmology.

[86] Durbin M.K., An L., Shemonski N.D. (2017). Quantification of retinal microvascular density in optical coherence tomographic angiography images in diabetic retinopathy. JAMA Ophthalmol.

[87] Roorda A., Romero-Borja F., Donnelly W., Queener H., Hebert T., Campbell M. (2002). Adaptive optics scanning laser ophthalmoscopy. Opt Express.

[88] Liang J., Williams D.R., Miller D.T. (1997). Supernormal vision and high-resolution retinal imaging through adaptive optics. J Opt Soc Am A Opt Image Sci Vis.

[89] Burns S.A., Elsner A.E., Chui T.Y. (2014). In vivo adaptive optics microvascular imaging in diabetic patients without clinically severe diabetic retinopathy. Biomed Opt Express.

[90] Tam J., Dhamdhere K.P., Tiruveedhula P. (2011). Disruption of the retinal parafoveal capillary network in type 2 diabetes before the onset of diabetic retinopathy. Invest Ophthalmol Vis Sci.

[91] Palochak C.M.A., Lee H.E., Song J. (2019). Retinal blood velocity and flow in early diabetes and diabetic retinopathy using adaptive optics scanning laser ophthalmoscopy. J Clin Med.

