# Supplementary material for: A New Approach to Staging Diabetic Eye Disease: Staging of Diabetic Retinal Neurodegeneration and Diabetic Macular Edema
Source: Ophthalmol Sci. 2023 Oct 31;4(3):100420. doi: 10.1016/j.xops.2023.100420 (PMC10818256; doi:10.1016/j.xops.2023.100420)
Supplement: Table S1 [file mmc1.pdf]

**Parameter**	
Parameter name	AOSLO Retinal Microstructure
Search terms	(AOSLO OR ”adaptive optics” OR “cone density”) (“diabetic retinopathy” OR exudate OR microaneurysm)
Search results	September 21, 2021: 77 papers
Pruning	Not diabetes; not structural; vessel structure; vessel diameter; not AOSLO as outcome parameter
How is this parameter assessed?	Retinal structure can be assessed at the microscopic level using adaptive optics scanning laser ophthalmoscopy (AOSLO) technology. A non-invasive imaging technique, AOSLO captures *en face*, black-and-white images of the retina with a resolution of approximately 2 microns.[1-3] Thus, it is uniquely capable of imaging individual retinal cells *in vivo*.<br><br>AOSLO is performed by obtaining videos of the posterior pole stepwise across the area of interest, generally the central 1 to 10 degrees. The patient must fixate on a light source throughout the duration of the exam. After the exam is complete, software averages each video corresponding to a retinal locus into a representative still frame. Multiple stills are then assembled into a montage.<br><br>Once the montage has been assembled, it is possible to analyze structural characteristics of photoreceptors and small blood vessels in the foveal and parafoveal region. This is usually done manually, although some automated programs have been developed for the research setting.<br><br>Assessment can focus on one or more of the following parameters:<br>• photoreceptor density and spacing<br>• microaneurysm (MA) density, diameter, perfusion status, intraluminal hyperreflectivity, and/or wall hyperreflectivity<br>• capillary density and diameter<br>• vascular tortuosity<br>• foveal avascular zone (FAZ) size and shape<br>• blood velocity

	**Furthermore, it is possible to image retinal ganglion cells (GC) using confocal AOSLO.[4] Additional studies are needed to determine if GC density and/or other characteristics change measurably (i.e. detectably with AOSLO) in pathologic conditions.**<br><br>**Ongoing developments in AOSLO technology include wide-field imaging,[5] which would permit visualization of extra-foveal retinal features.**
Has analytical validation been accomplished?<br>If yes, give specifics of evaluation of parameter precision, accuracy, limit of detection, limit of quantitation, specificity, linearity and range, ruggedness and robustness.	Because AOSLO machines and analysis techniques vary from one imaging center to another, it is difficult to validate the parameters in a generalized manner. Most assessments of reliability compare grading of cone spacing measurements, which have been found to have good intergrader reliability with manual grading as well as good performance by grading using an automated convolutional neural network.[6,7] However, more work is needed to standardize the methods of data acquisition and analysis.
What kind of variable is this (e.g. a binary event, time to event or quantitative/continuous outcome)?	Continuous: photoreceptor density, MA density, MA diameter, capillary density, capillary diameter, vascular tortuosity, FAZ size, blood velocity<br><br>Categorical: photoreceptor spacing (e.g., 4, 6, or 8 cones surrounding cone of interest), FAZ shape<br><br>Binary: MA perfusion status (perfused/not), MA intraluminal hyperreflectivity (present/absent), MA wall hyperreflectivity (present/absent)
Are there useful cut points or thresholds for outcome use?	Photoreceptor density and spacing: Normative values have been established in multiple studies.[8-15] However, there are no standardized measures for determining a disease state.<br><br>Capillary diameter: In one study, eyes with DR have an average capillary diameter of 8.2 µm (standard deviation (SD 1.1 µm), while normals had an average diameter of 6.1 µm (SD of 0.75 µm) ($p<0.01$).[16] This suggests there may be a threshold at which capillaries could be deemed pathologic.<br><br>Capillary density: Tam et al. found decreased capillary density in patients with type 2 DM without DR compared to controls, but the difference was not statistically significant.[17]

	Blood velocity: Retinal blood velocity is variably affected in different subsets of the diabetic population;[18] therefore, it is difficult to establish a threshold for pathologic state.
**Scientific Understanding of Relationship to DRD**	
What is the biological, anatomic and/or functional rationale or plausibility for the association of this parameter with DRD?<br>(i.e., what is the degree to which diabetes triggers subsequent steps in a pathophysiologic pathway and the role of the parameter in that causal or outcome pathway)	Photoreceptors: Damage may occur due to direct toxic effects of hyperglycemia, and/or vascular insufficiency resulting in localized ischemia<br><br>Vasculature: Hyperglycemia leads to pericyte loss and basement membrane thickening. Capillaries increase in diameter as a result and may have alterations in blood flow. MAs result from pathologic pericyte loss and subsequent weakening of the capillary wall. Damage to endothelial cell integrity leads to fluid leakage into the interstitial space. Clinically, this manifests as diabetic macular edema (DME).
What is the current understanding of the molecular mechanism(s) underlying the alterations in this parameter in association with DRD?<br>(specify whether mechanisms are physiologic, pathologic or pharmacologic)	Photoreceptor spacing: Alterations in photoreceptor packing correlate with deep capillary plexus ischemia ($p=0.030$) and can be identified in patients with type 1 DM prior to the onset of clinical DR.[19,20]<br><br>Vascular tortuosity: Redistribution of leukocytes may underlie the measurable increase in A-V tortuosity seen in patients with type 2 DM but no clinical DR.[13,21]<br><br>MA wall hyperreflectivity: It is postulated that MA wall hyperreflectivity might correlate with increased basement membrane thickness, a feature which is characteristic of MAs on histopathological studies.[22] In one study of 30 eyes, MA wall hyperreflectivity was correlated with disorganization of the retinal inner layers (DRIL), as seen on SD-OCT ($p=0.03$).[23] DRIL has been associated with poorer visual outcomes in eyes with DME.[24,25]<br><br>FAZ characteristics: Capillary dropout occurs as a result of hyperglycemic damage to vasculature. This results in increased FAZ size and irregularity.<br><br>*All of the mechanisms described above are pathologic.*

What is the outcome measure with which this parameter is associated?	Parameters assessed using AOSLO could be associated with decreased visual sensitivity, although this has not been directly correlated to date.
What is the link between the parameter and the accepted clinical outcome measure?	AOSLO identifies structural retinal damage. Retinal microaneurysm severity has been shown clinically to correlate with DR progression.[26,27] Likewise, altered photoreceptor spacing is seen in diabetic macular ischemia,[10] although the exact relationship between spacing and visual function has not been well established in diseased eyes. Presumably, a decrease in photoreceptor density would imply decreased visual acuity. However, at least one study has demonstrated that microperimetry can detect functioning retina cells even in areas where photoreceptors are not optically apparent on AOSLO.[28]
**Performance Expectations in DRD**	
What sensitivity to detect change does this parameter provide compared to the current standard (if available)?	The resolution of AOSLO is ~2 to 2.5 µm, compared to approximately 5 µm with SD-OCT. AOSLO has the potential to detect alterations in retinal structure earlier than with conventional imaging tools (color fundus photography, FA, SD-OCT) because it of its increased resolution.<br><br>The current gold standard for assessment of retinal photoreceptor density is *ex vivo* histopathology, which cannot be performed in living eyes.<br><br>**Of note: Studies using confocal AOSLO to analyze photoreceptor characteristics would also necessitate additional imaging to help interpret pathology. (This is because lac k of reflectivity/imageability of cones is altered in pathologic states, as shown in literature on AOSLO in eyes with AMD and macular telangiectasia.[29-31]) Non-confocal, split-detection AOSLO, on the other hand, can image photoreceptors regardless of their waveguiding properties.[32,33]**
Is there consistency of response across species? If yes, please explain	n/a
Is there consistency of response across mechanistically or mechanically distinct interventions? If yes, please explain	n/a

Is there a dose response to the magnitude of changes in this parameter and changes in the clinical outcome?<br>If yes, please give specifics of that relationship	Currently unknown, as AOSLO studies looking at longitudinal visual outcomes are lacking. MA characteristics on AOSLO imaging did not correlate with baseline logMAR VA in one study's multivariate analysis.[18]
Is there a temporal relationship between changes in this parameter and the clinical outcome?<br>If yes, please give specifics of that relationship	Alterations in parafoveal capillaries can be detected through AOSLO prior to the development of clinical DR[13]<br><br>The temporal relationship between DR progression and AOSLO parameter changes has not been well characterized. A single longitudinal study of AOSLO in diabetic eyes showed that most measures remained relatively stable over a 16-month period.[16] Another study, which imaged a single eye with PDR over a span of 20 weeks, documented increased capillary bends and hairpins, but capillary diameter remained stable. Additionally, both progression <u>and</u> regression of MAs were noted.[34]
What is the specificity of changes in this parameter for DRD?	Photoreceptor loss may be seen in patients with non-diabetic retinal disease (e.g., macular telangiectasia, cone-rod degeneration). Although MAs are most commonly associated with DR, they may be found in other retinal vascular disease, such as hypertensive retinopathy and vascular occlusions.<br><br>Of note, AOSLO image acquisition may be affected by media opacity, such as cataract and vitreous hemorrhage. **The denser the cataract, the more likely AOSLO will fail to capture adequate images.[35]**
**Types of Data and Available for Evidential Evaluation**	
Are there preclinical studies that address the relationship of this parameter to outcomes in DRD?	No
If yes, please summarize the available evidence from *in silico* studies	n/a
References for *in silico* studies	n/a
If yes, please summarize the available evidence from *in vitro* studies	n/a

References for *in vitro* studies	n/a
If yes, please summarize the available evidence from *in vivo* studies	n/a
References for *in vivo* studies	n/a
Are there clinical studies that address the relationship of this parameter to outcomes in DRD?	Yes – 1 trial ongoing: "Long-term Morphological and Functional Retinal Changes After Topical Citicoline Administration in Patients With Mild Signs of Diabetic Retinopathy in Type 1 Diabetes Mellitus: Pilot Study" ClinicalTrials.gov Identifier: NCT04009980
If yes, which of the following clinical study types have been performed: systematic review, prospective randomized controlled trial, retrospective randomized controlled trial, cohort study, case/control study?	This is a prospective, interventional, randomized, double-masked, single center pilot study. The primary outcome measure is change in retinal sensitivity after 36 months of topical citicoline use. The secondary outcome measure is morphological changes over 36 months, as measured by AOSLO and SD-OCT.
If yes, please summarize the available evidence from clinical studies	Results have not been published as of November 2020.
References for clinical studies	n/a
Are there literature reviews that address the relationship of this parameter to outcomes in DRD?	No
References for literature reviews	n/a
Please give the Level of Evidence available from these combined studies (use Tables 1 and 2 below to determine Level of Evidence. For this purpose, please substitute "DRD parameter" for "tumor marker" or "marker" in Table 1)	n/a
**Statistical Considerations**	
What is the specific relationship of the parameter to clinical outcomes?<br>Please specify effect sizes and measures of variability	The quantitative relationship between retinal structural changes and visual outcomes has not yet been characterized.

What is the usefulness of the parameter or its thresholds for clinical or research decision making?	Clinical decision-making: AOSLO may permit detection of early retinal structural changes. Its utility would be strongest to identify patients with pre-clinical DR, allowing clinicians to counsel patients about the risk of vision loss and the importance of blood glucose and blood pressure management.<br><br>Research decision-making: Because AOSLO allows precise characterization of the degree of retinal photoreceptor loss, it could be used as a very sensitive research tool to differentiate outcomes among patient cohorts that otherwise appear clinically indistinct.
Are there covariates that should be adjusted for when considering this parameter?	1. Refractive error<br>2. Axial eye length<br>3. Possibly age – additional work is needed to form normative database across broad age range of patients
Are there any additional statistical considerations for the use of this parameter?	Resolution of AOSLO may be limited in moderate or severe DME.
**Gap Analysis**	
What are the gaps in the literature to prove or disprove the utility of this parameter?	• Lack of large sample sizes<br>• Lack of prospective longitudinal studies correlating changes in AOSLO microstructure with risk of DR progression<br>• Lack of generalizability to eyes with DME, which are often excluded from AOSLO studies
In your opinion, what clinical research study/studies could address these gaps?	Ideally, one would design a prospective, longitudinal study to identify whether alterations in AOSLO microstructure correlate with 1) visual acuity and/or field loss, 2) risk of DR progression.
Are there currently available datasets that could be used for these validation efforts?	Possibly. A large observational cohort study (N = 600 eyes) to assess qualitative and quantitative features of AO images in diseased vs. normal eyes is ongoing as of November 2020. (ClinicalTrials.gov Identifier: NCT02317328) Results from this study would not be longitudinal nor specific to DR but may help solidify quantitative thresholds for pathologic retinal changes.

	Note: An observational cohort study was initiated in 2016 to assess peripheral retina structure differences between healthy controls and participants with diabetic retinopathy. However, it was terminated due to lack of funding. (ClinicalTrials.gov Identifier: NCT02826655)
**Miscellaneous Questions**	
Is this parameter currently employed in clinical use?	No
Is assessment instrumentation needed to measure this parameter currently: available commercially, available but not FDA approved, not readily available, or not available?	Available commercially. Not FDA approved.<br><br>Many of the AOSLO systems used at academic centers are non-identical and not commercially available.
What is the ease of implementation in the following environments: high resource academic center, high resource community practice, low resource/underserved environment?	Implementation limited by cost-effectiveness and labor-intensive nature of image post-processing. Currently could be implemented in high-resource academic centers.
What is the duration to measure this parameter in the average patient (order of magnitude, i.e. 1min, 10min, 100min)	10 to 30 minutes per eye. In general, the time is proportional to the amount of retinal surface area under investigation.
Please comment on "patient friendliness" metrics (e.g. invasiveness, use of contrast dye, claustrophobia, distance to operator, ability to use given COVID-19 considerations)	Non-invasive. Patient must be able to sit still for duration of imaging. It would be similar to an extended visual field test in terms of physical requirements. Some units require operator to be positioned in close proximity to patient. Utility potentially limited in the setting of cognitive impairment, severe orthopedic problems, and any other conditions that prevent the patient from positioning at the machine comfortably.
What sites are appropriate for this assessment? Indicate all relevant site types: retina clinic, general ophthalmology clinic, optometry clinic, endocrinology clinic, general medical clinic, patient home.	Retina clinic in tertiary care center
Is there any technology or advance either currently available, in development, or not yet developed that would make this parameter no longer important or relevant?	Yes – OCT +/- OCT-A with increased resolution has the potential to replace some of the need for AOSLO image acquisition

If yes, please specify what technology or advance	**Adaptive Optics OCT (AO-OCT) is a developing technology that obtains very high-resolution scans of the retina and displays them as axial sections, as with traditional SD-OCT.**
What unmet need in the staging of DRD does this parameter address?	• Detection of pre-clinical structural retinal damage<br>• Potential for high sensitivity measurements of retinal ischemia<br>• Structural correlation between neural and vascular dysfunction (e.g., photoreceptor loss and microaneurysms)
**Summary**	
Based on the above data, please provide an integrated evaluation regarding the overall importance of this parameter to the field currently. If not currently relevant, please summarize the potential for future relevance, necessary steps for validation and a reasonable time frame for this to occur.	AOSLO is a non-invasive technology with capability to assess multiple elements of the retinal microstructure, including vascular changes and photoreceptor degeneration. Although most studies of AOSLO to date have focused on normal eyes and inherited retinal degenerations, there are a number of studies demonstrating AOSLO's potential to identify microscopic changes in DR.<br><br>Two principal limitations that prevent AOSLO from widespread use: the time-intensive nature of image acquisition and analysis, as well as the limited availability of devices. The technology is mostly used in research settings at present. If AOSLO were to become a more widespread or commercially viable technology, it would be important to develop optical and analytical methods for dealing with DME, which affects image quality. This would be critical for long-term studies in DR.<br><br>Additionally, AOSLO would benefit from a large study in order to expand the normative data base, especially among older patients. Once standardized data sets are in use, there would be potential for longitudinal study of the natural history of DR (and possibly prospective controlled trials).
Additional references	Wynne, et al., 2021 PMID 33161127.[36]<br>AbdelAl, et al., 2019 PMID 31188056.[37]<br>Cristescu, et al., 2019 PMID 31198895.[38]<br>Cristescu, et al., 2019 PMID 32377236.[39]

	Yamaguchi, et al, PMID 27641223.[40]<br>Ro-Mase , et al., PMID 32492109.[41]<br>Parravano, et al., 2020. PMID 32180131.[42]

References

1. Liang J, Williams DR, Miller DT. Supernormal vision and high-resolution retinal imaging through adaptive optics. *J Opt Soc Am A Opt Image Sci Vis.* 1997;14(11):2884-2892.
2. Roorda A, Romero-Borja F, Donnelly Iii W, Queener H, Hebert T, Campbell M. Adaptive optics scanning laser ophthalmoscopy. *Opt Express.* 2002;10(9):405-412.
3. Zhang Y, Poonja S, Roorda A. MEMS-based adaptive optics scanning laser ophthalmoscopy. *Opt Lett.* 2006;31(9):1268-1270.
4. Rossi EA, Granger CE, Sharma R, et al. Imaging individual neurons in the retinal ganglion cell layer of the living eye. *Proc Natl Acad Sci U S A.* 2017;114(3):586-591.
5. Ferguson RD, Zhong Z, Hammer DX, et al. Adaptive optics scanning laser ophthalmoscope with integrated wide-field retinal imaging and tracking. *J Opt Soc Am A Opt Image Sci Vis.* 2010;27(11):A265-277.
6. Cunefare D, Huckenpahler AL, Patterson EJ, Dubra A, Carroll J, Farsiu S. RAC-CNN: multimodal deep learning based automatic detection and classification of rod and cone photoreceptors in adaptive optics scanning light ophthalmoscope images. *Biomed Opt Express.* 2019;10(8):3815-3832.
7. Zayit-Soudry S, Sippl-Swezey N, Porco TC, et al. Repeatability of Cone Spacing Measures in Eyes With Inherited Retinal Degenerations. *Invest Ophthalmol Vis Sci.* 2015;56(10):6179-6189.
8. Curcio CA, Sloan KR, Kalina RE, Hendrickson AE. Human photoreceptor topography. *J Comp Neurol.* 1990;292(4):497-523.
9. Curcio CA, Sloan KR. Packing geometry of human cone photoreceptors: variation with eccentricity and evidence for local anisotropy. *Vis Neurosci.* 1992;9(2):169-180.
10. Merino D, Duncan JL, Tiruveedhula P, Roorda A. Observation of cone and rod photoreceptors in normal subjects and patients using a new generation adaptive optics scanning laser ophthalmoscope. *Biomed Opt Express.* 2011;2(8):2189-2201.
11. Godara P, Wagner-Schuman M, Rha J, Connor TB, Jr., Stepien KE, Carroll J. Imaging the photoreceptor mosaic with adaptive optics: beyond counting cones. *Adv Exp Med Biol.* 2012;723:451-458.
12. Bidaut Garnier M, Flores M, Debellemanière G, et al. Reliability of cone counts using an adaptive optics retinal camera. *Clin Exp Ophthalmol.* 2014;42(9):833-840.
13. Liu BS, Tarima S, Visotcky A, et al. The reliability of parafoveal cone density measurements. *Br J Ophthalmol.* 2014;98(8):1126-1131.
14. Zhang T, Godara P, Blanco ER, et al. Variability in Human Cone Topography Assessed by Adaptive Optics Scanning Laser Ophthalmoscopy. *Am J Ophthalmol.* 2015;160(2):290-300.e291.
15. Wells-Gray EM, Choi SS, Bries A, Doble N. Variation in rod and cone density from the fovea to the mid-periphery in healthy human retinas using adaptive optics scanning laser ophthalmoscopy. *Eye (Lond).* 2016;30(8):1135-1143.
16. Burns SA, Elsner AE, Chui TY, et al. In vivo adaptive optics microvascular imaging in diabetic patients without clinically severe diabetic retinopathy. *Biomed Opt Express.* 2014;5(3):961-974.

17. Tam J, Dhamdhere KP, Tiruveedhula P, et al. Disruption of the retinal parafoveal capillary network in type 2 diabetes before the onset of diabetic retinopathy. *Invest Ophthalmol Vis Sci.* 2011;52(12):9257-9266.
18. Palochak CMA, Lee HE, Song J, et al. Retinal Blood Velocity and Flow in Early Diabetes and Diabetic Retinopathy Using Adaptive Optics Scanning Laser Ophthalmoscopy. *J Clin Med.* 2019;8(8).
19. Lombardo M, Parravano M, Serrao S, Ziccardi L, Giannini D, Lombardo G. Investigation of Adaptive Optics Imaging Biomarkers for Detecting Pathological Changes of the Cone Mosaic in Patients with Type 1 Diabetes Mellitus. *PLoS One.* 2016;11(3):e0151380.
20. Nesper PL, Scarinci F, Fawzi AA. Adaptive Optics Reveals Photoreceptor Abnormalities in Diabetic Macular Ischemia. *PLoS One.* 2017;12(1):e0169926.
21. Tam J, Dhamdhere KP, Tiruveedhula P, et al. Subclinical capillary changes in non-proliferative diabetic retinopathy. *Optom Vis Sci.* 2012;89(5):E692-703.
22. Stitt AW, Gardiner TA, Archer DB. Histological and ultrastructural investigation of retinal microaneurysm development in diabetic patients. *Br J Ophthalmol.* 1995;79(4):362-367.
23. Lammer J, Karst SG, Lin MM, et al. Association of Microaneurysms on Adaptive Optics Scanning Laser Ophthalmoscopy With Surrounding Neuroretinal Pathology and Visual Function in Diabetes. *Invest Ophthalmol Vis Sci.* 2018;59(13):5633-5640.
24. Sun JK, Radwan SH, Soliman AZ, et al. Neural Retinal Disorganization as a Robust Marker of Visual Acuity in Current and Resolved Diabetic Macular Edema. *Diabetes.* 2015;64(7):2560-2570.
25. Sun JK, Lin MM, Lammer J, et al. Disorganization of the retinal inner layers as a predictor of visual acuity in eyes with center-involved diabetic macular edema. *JAMA Ophthalmol.* 2014;132(11):1309-1316.
26. Klein R, Meuer SM, Moss SE, Klein BE. The relationship of retinal microaneurysm counts to the 4-year progression of diabetic retinopathy. *Arch Ophthalmol.* 1989;107(12):1780-1785.
27. Ribeiro ML, Nunes SG, Cunha-Vaz JG. Microaneurysm turnover at the macula predicts risk of development of clinically significant macular edema in persons with mild nonproliferative diabetic retinopathy. *Diabetes Care.* 2013;36(5):1254-1259.
28. Tu JH, Foote KG, Lujan BJ, et al. Dysflective cones: Visual function and cone reflectivity in long-term follow-up of acute bilateral foveolitis. *Am J Ophthalmol Case Rep.* 2017;7:14-19.
29. Litts KM, Wang X, Clark ME, et al. Exploring photoreceptor reflectivity through multimodal imaging of outer retinal tubulation in advanced age-related macular degeneration. *Retina.* 2017;37(5):978-988.
30. Wang Q, Tuten WS, Lujan BJ, et al. Adaptive optics microperimetry and OCT images show preserved function and recovery of cone visibility in macular telangiectasia type 2 retinal lesions. *Invest Ophthalmol Vis Sci.* 2015;56(2):778-786.
31. Zhang Y, Wang X, Rivero EB, et al. Photoreceptor perturbation around subretinal drusenoid deposits as revealed by adaptive optics scanning laser ophthalmoscopy. *Am J Ophthalmol.* 2014;158(3):584-596.e581.
32. Scoles D, Sulai YN, Langlo CS, et al. In vivo imaging of human cone photoreceptor inner segments. *Invest Ophthalmol Vis Sci.* 2014;55(7):4244-4251.
33. Sun LW, Johnson RD, Williams V, et al. Multimodal Imaging of Photoreceptor Structure in Choroideremia. *PLoS One.* 2016;11(12):e0167526.
34. Chui TY, Pinhas A, Gan A, et al. Longitudinal imaging of microvascular remodelling in proliferative diabetic retinopathy using adaptive optics scanning light ophthalmoscopy. *Ophthalmic Physiol Opt.* 2016;36(3):290-302.
35. Zhang Y, Wang X, Clark ME, Curcio CA, Owsley C. Imaging of Age-Related Macular Degeneration by Adaptive Optics Scanning Laser Ophthalmoscopy in Eyes With Aged Lenses or Intraocular Lenses. *Transl Vis Sci Technol.* 2020;9(8):41.

36. Wynne N, Carroll J, Duncan JL. Promises and pitfalls of evaluating photoreceptor-based retinal disease with adaptive optics scanning light ophthalmoscopy (AOSLO). *Prog Retin Eye Res.* 2021;83:100920.
37. AbdelAl O, Ashraf M, Sampani K, Sun JK. "For Mass Eye and Ear Special Issue" Adaptive Optics in the Evaluation of Diabetic Retinopathy. *Semin Ophthalmol.* 2019;34(4):189-197.
38. Cristescu IE, Ochinciuc R, Balta F, Zagrean L. High-resolution imaging of diabetic retinopathy lesions using an adaptive optics retinal camera. *Rom J Ophthalmol.* 2019;63(1):29-34.
39. Cristescu IE, Zagrean L, Balta F, Branisteanu DC. Retinal Microcirculation Investigation in Type I and II Diabetic Patients Without Retinopathy Using an Adaptive Optics Retinal Camera. *Acta Endocrinol (Buchar).* 2019;15(4):417-422.
40. Yamaguchi M, Nakao S, Kaizu Y, et al. High-Resolution Imaging by Adaptive Optics Scanning Laser Ophthalmoscopy Reveals Two Morphologically Distinct Types of Retinal Hard Exudates. [Erratum in: Sci Rep. 2016 Nov 03;6:35127]. *Sci Rep.* 2016;6:33574.
41. Ro-Mase T, Ishiko S, Omae T, Ishibazawa A, Shimouchi A, Yoshida A. Association Between Alterations of the Choriocapillaris Microcirculation and Visual Function and Cone Photoreceptors in Patients With Diabetes. *Invest Ophthalmol Vis Sci.* 2020;61(6):1.
42. Parravano M, Scarinci F, Parisi V, et al. Citicoline and Vitamin B(12) Eye Drops in Type 1 Diabetes: Results of a 3-year Pilot Study Evaluating Morpho-Functional Retinal Changes. *Adv Ther.* 2020;37(4):1646-1663.
