# Supplementary material for: A New Approach to Staging Diabetic Eye Disease: Staging of Diabetic Retinal Neurodegeneration and Diabetic Macular Edema
Source: Ophthalmol Sci. 2023 Oct 31;4(3):100420. doi: 10.1016/j.xops.2023.100420 (PMC10818256; doi:10.1016/j.xops.2023.100420)
Supplement: Table S2 [file mmc2.pdf]

**Parameter**	
Parameter name	Center-involved diabetic macular edema (ci-DME) as measured with Optical coherence tomography (OCT)
Pubmed search terms	(OCT or "optical coherence tomography") AND thickness AND outcome AND diabetes AND ("diabetic macular edema" OR "center involved")
Search results	667; Sept 21, 2021
Pruning	Not vitrectomy, not surgery, not cataract, not phacoemulsification,<br>Not photocoagulation, not vitreomacular adhesion, not inflammation, not treat and extend, not vessel, not angiography, not OCTa; epiretinal; not perimetry; not microperimetry
How is this parameter assessed?	DME may be assessed through the following methods:<br>1. Slit-lamp exam w/ or w/o macular contact lens<br>2. Optical coherence tomography (OCT)<br>3. Fluorescein angiography<br>The following variables can be assessed with OCT:<br>• Central point or foveal thickness (CPT)<br>• Central subfield or macular thickness (CST, CMT) – circular area of diameter 1 mm centered at fovea<br>• Macular volume<br>• Presence or absence of ci-DME<br>• Severity of ci-DME<br>• Time to development of ci-DME<br>• Serous retinal detachment<br>• Intensity of hyperreflective dots<br>• Number, volume or area, size of cysts<br>• Polarization<br>• Choroidal thickness<br>• Morphologic pattern of DME (diffuse thickening, cystoid, subretinal fluid, etc.)
Has analytical validation been accomplished?<br>If yes, give specifics of evaluation of parameter precision, accuracy, limit of	2015 Cochrane review for diagnostic accuracy of OCT for detecting clinically significant DME compared to fundus biomicroscopy: 9 studies (759 participants, 1303 eyes), pooled

detection, limit of quantitation, specificity, linearity and range, ruggedness and robustness.	sensitivity 0.78 (95% confidence interval (CI) 0.72 to 0.83) and specificity 0.86 (95% CI 0.76 to 0.93).[11]<br><br>Coefficients of repeatability for CMT and CPE in DME are 11% and 17%, respectively.[12] Therefore, CMT may be a more precise parameter.
What kind of variable is this (e.g. a binary event, time to event or quantitative/continuous outcome)?	Could be a categorical variable (DME severity; morphologic pattern), binary event (presence of DME yes or no), time-to- event (time to development of DME), or continuous variable (central macular thickness; macular volume; reflectivity of cysts)
Are there useful cut points or thresholds for outcome use?	Value for central macular thickness is automatically generated by commercial OCT upon completion of scan. This value can be compared to population norms.<br><br>As defined in DRCR papers, the threshold for diagnosis of DME was mean OCT retinal thickness of ≥250 μm in the central subfield or ≥300 μm in at least one of the four inner subfields.[13, 14]
**Scientific Understanding of Relationship to DRD**	
What is the biological, anatomic and/or functional rationale or plausibility for the association of this parameter with DRD?<br>(i.e., what is the degree to which diabetes triggers subsequent steps in a pathophysiologic pathway and the role of the parameter in that causal or outcome pathway)	Hyperglycemia leads to pericyte loss and basement membrane thickening. Neurodegeneration may play a role. Capillaries increase in diameter as a result and may have alterations in blood flow. MAs result from pathologic pericyte loss and subsequent weakening of the capillary wall. Damage to endothelial cell integrity leads to fluid leakage into the interstitial space. Clinically, this manifests as diabetic macular edema (DME).
What is the current understanding of the molecular mechanism(s) underlying the alterations in this parameter in association with DRD?<br>(specify whether mechanisms are physiologic, pathologic or pharmacologic)	Pathologic alterations in DR include elevated extracellular glutamate, enzymatic glycation of proteins, increased polyol production, and activation of protein kinase C. These changes cause oxidative stress and alterations in cell protein transcription, which have subsequent downstream effects. There are likely many factors involved. However, in summary, these jointly contribute to the breakdown of blood-retina-barrier junctions and the leakage of fluid into the interstitial space.

	The degree hydrostatic pressure in the retinal vasculature, as well as the osmotic pressure in the extracellular environment from hyperglycemic products, influence the severity of DME.<br><br>The following paper outlines molecular mechanisms of DME: Romero-Aroca et al., 2016.[15]
What is the outcome measure with which this parameter is associated?	Visual acuity, color vision, contrast
What is the link between the parameter and the accepted clinical outcome measure?	There is a modest correlation between BCVA and the OCT-measured central foveal thickness in a 2007 DRCR report, which found that, on average, baseline was 4.4 (95% C.I.: 3.5, 5.3) letters better for every 100 microns decrease in center point thickness.[14]<br><br>Other studies have shown similar correlation coefficients of 0.574,[16] 0.558,[17] and 0.56.[18] It is not known whether stratifying these data by patient sex and/or specific thicknesses of individual retinal layers would increase the correlation with VA.<br><br>Other factors may influence visual outcome above and beyond the degree of DME. Macular ischemia, retinal structure alterations, and/or thinning of neuroretinal layers are probable co-variates in the equation. For example, disorganization of the retinal inner layers (DRIL) correlates with reduced visual acuity and contrast sensitivity, and has prognostic significance with regard to short-term (<1 year) visual outcomes of DME patients.[19, 20]<br><br>One DRCR study that modeled factors affecting visual acuity in DME subjects found that OCT CMT, age, fluorescein leakage, and number of thickened OCT subfields did correlate with visual acuity in a multivariate model. Duration of diabetes, ETDRS retinopathy stage, sex, and HbA1C did not show a significant correlation with VA. It should be noted that none of the patients included in this study had high-risk PDR at

	baseline[21]
**Performance Expectations in DRD**	
What sensitivity to detect change does this parameter provide compared to the current standard (if available)?	Likely more sensitive than serial fundus examinations; however, it is unknown what threshold of change in ci-DME is clinically significant (may vary from one eye to another). Additionally, sensitivity to detect change could depend on whether you are measuring <u>relative</u> change in thickness or <u>absolute</u> change in thickness.
Is there consistency of response across species?<br>If yes, please explain	n/a
Is there consistency of response across mechanistically or mechanically distinct interventions?<br>If yes, please explain	Yes. Decreased CMT has been noted in response to anti-VEGF (reference: DRCR studies), corticosteroids,[22, 23] focal laser,[24-26] and improved glucose control[27, 28]
Is there a dose response to the magnitude of changes in this parameter and changes in the clinical outcome?<br>If yes, please give specifics of that relationship	Li et al.: OCT parameters, including CMT, predicted 50-60% of variation in BCVA in eyes with DME[29]<br><br>Hatef et al.: Macular sensitivity increased by average of 0.03 dB per 1-micron (µm) increase in retinal thickness for thickness ≤280 µm and by 0.05 dB per 1 micron (µm) increase in retinal thickness for values >280 µm[30]
Is there a temporal relationship between changes in this parameter and the clinical outcome?<br>If yes, please give specifics of that relationship	Yes, decrease in CMT occurs simultaneously with improvement in BCVA in studies of anti-VEGF therapy.[31]
What is the specificity of changes in this parameter for DRD?	Non-specific. Also seen in other inflammatory/vascular conditions (post-operative, uveitis, vascular occlusion) and some retinal degenerations.
**Types of Data and Available for Evidential Evaluation**	
Are there preclinical studies that address the relationship of this parameter to outcomes in DRD?	In rat models of Type 2 diabetes, DME-like changes have been noted. However, difficult to measure visual outcomes in rats.<br>Limitations of preclinical models for DME is first and foremost due to lack of macula in rodents; however, models have been proposed to assess mechanisms of retinal

	thickening. Another limitation is the extent of the thickening in any of the models used, potentially due to duration of disease and modeling (all rather short). Finally and maybe the major limitation is the counterbalance of retinal thinning due to neurodegeneration.
If yes, please summarize the available evidence from *in silico* studies	n/a
References for *in silico* studies	n/a
If yes, please summarize the available evidence from *in vitro* studies	n/a
References for *in vitro* studies	n/a
If yes, please summarize the available evidence from *in vivo* studies	Spontaneously Diabetic Torii (SDT) fatty rats show retinal thickening at 16 to 24 weeks of age.<br>The involvement of VEGF and TNFalpha has been proposed and tested through single intravitreal injection, leading to modest increase in retinal thickness[32] (+10%), and reversed by atypical PKC inhibitor.<br>Another mechanism proposed involves a VEGF independent regulator of vascular permeability involving the plasma kallikrein-kinin system. A first study reported a 6% increase in thickness in STZ rats[33] and both studies reported that diabetes combined with intravitreal injection with modulators of the pathway lead to further increase.[34]<br>All rodent models demonstrate more propensity for retinal thinning (associated with neurodegeneration), more so than with the retinal thickening due to vasculature alterations and inflammation. Starting from studies by Barber et al.,[35] confirmed by OCT in Ins2Akita[36] or stz mice.[37, 38] Molecularly, even the VEGF overexpressing diabetic Akimba model is characterized by retinal thinning[39] (neurodegeneration). Mechanistically, propose role of inflammation and leukostasys[40] but still debate about vasogenic vs inflammatory pathophysiology of DME.[15] Potential modeling through approach similar to that used in brain[41] and simulating vasogenic brain edema using chronic infusion – still need to define what to infuse though.
References for *in vivo* studies	1. Motohashi, et al., 2018. PMID 29527913.[42]<br>2. Tanaka, et al., 2019. PMID 31637263.[43]<br>3. Lin, et al., 2018. PMID: 30220554.[32]<br>4. Clermont, et al., 2011. PMID: 21444925.[33]<br>5. Kita, et al., 2015. PMID: 25979073.[34]

	6. Barber, et al., 2005. PMID: 15914643.[35]<br>7. Sohn, et al., 2016. PMID: 27114552.[38]<br>8. Francis, et al., 2018. PMID: 29675273.[36]<br>9. Ruebsam, et al., 2018. PMID: 29467334.[37]<br>10. Rakoczy ,et al., 2010. PMID: 20829433.[39]<br>11. Liu, et al, 2017. PMID: 28931763.[40]<br>12. Romero-Aroca, et al., 2016. PMID: 27761468.[15]<br>13. Piazza, et al., 2017. PMID: 28059647[41]
Are there clinical studies that address the relationship of this parameter to outcomes in DRD?	Yes
If yes, which of the following clinical study types have been performed: systematic review, prospective randomized controlled trial, retrospective randomized controlled trial, cohort study, case/control study?	<u>Retrospective, observational, cross-sectional studies:</u><br>Li B, et al., 2020. [29]<br><br><u>Prospective, observational, cross-sectional studies:</u><br>Diabetic Retinopathy Clinical Research Network, 2007.[14]<br>Islam, 2016.[16]<br>Hannouche and Avila, 2009.[44]<br>Khojasteh, et al., 2020.[45]<br>Xia, et al., 2020.[46]<br><br><u>There are numerous prospective RCTs</u> that have evaluated visual acuity and CMT in the setting of DME therapy. Examples include:<br>Bressler, et al., 2018.[47]<br>Wells, et al., 2016.[48]
If yes, please summarize the available evidence from clinical studies	There is a moderate correlation.
References for clinical studies	See above

Are there literature reviews that address the relationship of this parameter to outcomes in DRD?	Yes
References for literature reviews	Nagai, et al., 2020.[10]
Please give the Level of Evidence available from these combined studies (use Tables 1 and 2 below to determine Level of Evidence. For this purpose, please substitute "DRD parameter" for "tumor marker" or "marker" in Table 1)	Level I<br>• Category B: prospective RCTs have included CMT as a secondary outcome<br>• 1 or more validation studies with consistent results about correlation between CMT and visual acuity
**Statistical Considerations**	
What is the specific relationship of the parameter to clinical outcomes?<br>Please specify effect sizes and measures of variability	
What is the usefulness of the parameter or its thresholds for clinical or research decision making?	High utility, as the presence and/or worsening of ci-DME may prompt treatment with intravitreal anti-VEGF, focal macular laser
Are there covariates that should be adjusted for when considering this parameter?	Covariates that influence baseline OCT measurements of retinal thickness:<br>• Patient sex (males have greater CMT in DME and diabetes without DME)[49, 50]<br>• OCT machine (e.g., Zeiss Cirrus vs. Heidelberg Spectralis)<br>• Refractive error<br><br>Covariates that may influence severity of DME:<br>• Glycemic control<br>• Age<br>• Diabetes duration<br>• Hypertension<br>• Renal function<br><br>Note: Glycemic control, age, and diabetes duration were not been shown to be predictive variabkes in 1 small study of DME response to PRN anti-VEGF treatment[51]<br><br>Covariates that influence visual acuity:

	• Disorganization of inner retinal layers<br>• Retinal neurodegeneration<br>• Macular ischemia<br>• DME duration<br>• DME & diabetes duration/neurodegeneration. UKBB
Are there any additional statistical considerations for the use of this parameter?	DRCR study of diurnal variation in CMT (156 eyes, 96 subjects) found 6% mean decrease in relative CMT when measured at 4 p.m. compared to 8 a.m.[52] Other studies have corroborated this trend.[53, 54]<br><br>Some studies of DME have found that CMT measurements in the same eye can fluctuate in the short term,[55] which may increase the threshold needed to determine if there is a significant clinical change. Other studies have not found this to be the case.[56]
**Gap Analysis**	
What are the gaps in the literature to prove or disprove the utility of this parameter?	Multiple studies have suggested foveal/parafoveal retinal tissue integrity serve as a predictor of visual acuity in DME.[57, 58] The literature does not contain a multivariate equation that correlates OCT-based thickness with visual acuity while accounting for other factors that independently influence visual potential. Furthermore, it is not clear if eyes that receive repeated anti-VEGF treatments have an increased risk of ocular hypertension, glaucoma, and/or RNFL thinning, all of which could influence retinal thickness measurements; the literature on this topic is mixed.[59-62]
In your opinion, what clinical research study/studies could address these gaps?	Longitudinal, prospective observational cohort study in eyes with/without DME and with/without DR; evaluation of relative strength of the following factors as predictive of visual acuity changes: central foveal thickness, central macular thickness, macular volume, presence of DRIL, IS/OS integrity, thickness of each retinal layer
Are there currently available datasets that could be used for these validation efforts?	Yes; OCT scans from DRCR trials could be analyzed
**Miscellaneous Questions**	
Is this parameter currently employed in clinical use?	Yes
Is assessment instrumentation needed to measure this parameter currently: available	Available commercially; multiple manufacturers

commercially, available but not FDA approved, not readily available, or not available?	
What is the ease of implementation in the following environments: high resource academic center, high resource community practice, low resource/underserved environment?	Relatively easy in high-resource practice. Could utilize older models in low-resource setting, and these would still provide accurate data.
What is the duration to measure this parameter in the average patient (order of magnitude, i.e. 1min, 10min, 100min)	30 seconds to 3 minutes per eye
Please comment on "patient friendliness" metrics (e.g. invasiveness, use of contrast dye, claustrophobia, distance to operator, ability to use given COVID-19 considerations)	Non-invasive, some units can accommodate wheelchairs.<br><br>Positioning at the machine rarely a problem; can occur with severe orthopedic problems and or moderate/severe cognitive impairment.
What sites are appropriate for this assessment?<br>Indicate all relevant site types: retina clinic, general ophthalmology clinic, optometry clinic, endocrinology clinic, general medical clinic, patient home.	All clinical sites are potential options.<br><br>If done at medical or endocrine clinic, would require some training for clinic staff on device operation.<br><br>Note: The Notal Home OCT device is FDA-approved for monitoring neovascular AMD, but not for DME.
Is there any technology or advance either currently available, in development, or not yet developed that would make this parameter no longer important or relevant?<br>If yes, please specify what technology or advance	No, but it may turn out that biomarkers other than DME – such as disorganization of inner retinal layers (DRIL) – have a more significant correlation with vision loss
What unmet need in the staging of DRD does this parameter address?	A significant proportion of DR-associated vision loss is due to macular edema.<br><br>ciDME severity does not correlate linearly with the stage of clinical (vascular) DR,[63] so it represents an important additional metric for consideration.
**Summary**	

Based on the above data, please provide an integrated evaluation regarding the overall importance of this parameter to the field currently. If not currently relevant, please summarize the potential for future relevance, necessary steps for validation and a reasonable time frame for this to occur.	ci-DME has been well documented as a predictor of decreased VA in patients with diabetes. However, the correlation is moderate. There are likely other factors, such as retinal neural integrity, that influence visual function in these eyes.<br><br>Advantages of CMT as a parameter are that it can be measured in a standardized fashion, is noninvasive, and equipment is relatively ubiquitous in ophthalmology clinical setting.<br><br>There are many existing OCT and VA data sets from longitudinal pharmacologic studies of diabetic macular edema. These could be further analyzed in a retrospective manner to determine if OCT features other than CMT are more predictive of outcomes.<br><br>In the future, it is possible that OCT will have the capability to automatically detect and quantify macular fluid using deep learning algorithms.
Additional references	Lai, et al., 2021 PMID 34012972.[1]<br>You, et al., 2021 PMID3393385.[2]<br>Moon, et al., 2021 PMID 33323903.[3]<br>Chen, et al., 2020 PMID 33262567.[4]<br>Furino, et al., 2020 PMID 33133444.[5]<br>Ciloglu, et al., 2020 PMID 33084813.[6]<br>Osathanugrah, et al., 2021 PMID 33045219.[7]<br>Vadalà, et al., 2020 PMID 32982158.[8]<br>Chatziralli, et al., 2021 PMID 32564022.[9]<br>Nagai, et al., 2020 PMID 32397232.[10]

References

1. Lai CT, Hsieh YT, Lin CJ, Wang JK, Lin CY, Hsia NY, et al. Age, Initial Central Retinal Thickness, and OCT Biomarkers Have an Influence on the Outcome of Diabetic Macular Edema Treated With Ranibizumab- Tri-center 12-Month Treat-and-Extend Study. Front Med (Lausanne). 2021;8:668107. doi: 10.3389/fmed.2021.668107. PubMed PMID: 34012972; PMCID: PMC8126636.

2. You QS, Tsuboi K, Guo Y, Wang J, Flaxel CJ, Bailey ST, et al. Comparison of Central Macular Fluid Volume With Central Subfield Thickness in Patients With Diabetic Macular Edema Using Optical Coherence Tomography Angiography. JAMA Ophthalmol. 2021;139(7):734-741. doi: 10.1001/jamaophthalmol.2021.1275. PubMed PMID: 33983385; PMCID: PMC8120439.

3. Moon KY, Choi SY, Song JH. Changes in subfoveal choroidal thickness after intravitreal dexamethasone implant therapy for diabetic macular edema. Retina. 2021;41(6):1283-1292. doi: 10.1097/iae.0000000000003029. PubMed PMID: 33323903; PMCID: PMC8140662.

4. Chen NN, Chen WD, Lai CH, Kuo CN, Chen CL, Huang JC, et al. Optical Coherence Tomographic Patterns as Predictors of Structural Outcome After Intravitreal Ranibizumab in Diabetic Macula Edema. Clin Ophthalmol. 2020;14:4023-4030. doi: 10.2147/opth.S264669. PubMed PMID: 33262567; PMCID: PMC7698720.

5. Furino C, Niro A, Reibaldi M, Boscia F, Alessio G. Efficacy of Intravitreal Dexamethasone Implant in Different Patterns of Diabetic Macular Edema. J Ophthalmic Vis Res. 2020;15(4):524-530. doi: 10.18502/jovr.v15i4.7787. PubMed PMID: 33133444; PMCID: PMC7591829.

6. Ciloglu E, Unal F, Dogan NC. Changes in the ganglion cell complex thickness after anti-VEGF treatment for diabetic macular edema. Arq Bras Oftalmol. 2020;83(5):372-377. doi: 10.5935/0004-2749.20200045. PubMed PMID: 33084813.

7. Osathanugrah P, Sanjiv N, Siegel NH, Ness S, Chen X, Subramanian ML. The Impact of Race on Short-term Treatment Response to Bevacizumab in Diabetic Macular Edema. Am J Ophthalmol. 2021;222:310-317. doi: 10.1016/j.ajo.2020.09.042. PubMed PMID: 33045219.

8. Vadalà M, Sunseri Trapani V, Guarrasi G, Ventura N, Castellucci M, Cillino S. A Real-World Study of Dexamethasone Implant in Treatment-Naïve Patients with Diabetic Macular Edema: Efficacy and Correlation Between Inflammatory Biomarkers and Treatment Outcome. Clin Ophthalmol. 2020;14:2657-2665. doi: 10.2147/opth.S257775. PubMed PMID: 32982158; PMCID: PMC7501979.

9. Chatziralli I, Kazantzis D, Theodossiadis G, Theodossiadis P, Sergentanis TN. Retinal Layers Changes in Patients with Diabetic Macular Edema Treated with Intravitreal Anti-VEGF Agents: Long-Term Outcomes of a Spectral-Domain OCT Study. Ophthalmic Res. 2021;64(2):230-236. doi: 10.1159/000509552. PubMed PMID: 32564022.

10. Nagai N, Suzuki M, Uchida A, Kurihara T, Ban N, Minami S, et al. The Area and Number of Intraretinal Cystoid Spaces Predict the Visual Outcome after Ranibizumab Monotherapy in Diabetic Macular Edema. J Clin Med. 2020;9(5). doi: 10.3390/jcm9051391. PubMed PMID: 32397232; PMCID: PMC7290842.

11. Virgili G, Menchini F, Casazza G, Hogg R, Das RR, Wang X, et al. Optical coherence tomography (OCT) for detection of macular oedema in patients with diabetic retinopathy. Cochrane Database Syst Rev. 2015;1:CD008081. doi: 10.1002/14651858.CD008081.pub3. PubMed PMID: 25564068; PMCID: PMC4438571.

12. Diabetic Retinopathy Clinical Research Network, Krzystolik MG, Strauber SF, Aiello LP, Beck RW, Berger BB, et al. Reproducibility of macular thickness and volume using Zeiss optical coherence tomography in patients with diabetic macular edema. Ophthalmology. 2007;114(8):1520-5. doi: 10.1016/j.ophtha.2006.10.055. PubMed PMID: 17353052; PMCID: PMC2253719.

13. Davis MD, Bressler SB, Aiello LP, Bressler NM, Browning DJ, Flaxel CJ, et al. Comparison of time-domain OCT and fundus photographic assessments of retinal thickening in eyes with diabetic macular edema. Invest Ophthalmol Vis Sci. 2008;49(5):1745-52. doi: 10.1167/iovs.07-1257. PubMed PMID: 18316700; PMCID: PMC2408888.

14. Diabetic Retinopathy Clinical Research Network, Browning DJ, Glassman AR, Aiello LP, Beck RW, Brown DM, et al. Relationship between optical coherence tomography-measured central retinal thickness and visual acuity in diabetic macular edema. Ophthalmology. 2007;114(3):525-36. doi: 10.1016/j.ophtha.2006.06.052. PubMed PMID: 17123615; PMCID: PMC2585542.

15. Romero-Aroca P, Baget-Bernaldiz M, Pareja-Rios A, Lopez-Galvez M, Navarro-Gil R, Verges R. Diabetic Macular Edema Pathophysiology: Vasogenic versus Inflammatory. J Diabetes Res. 2016;2016:2156273. doi: 10.1155/2016/2156273. PubMed PMID: 27761468; PMCID: PMC5059543.

16. Islam F. Retinal Thickness and Visual Acuity in Diabetic Macular Edema: An Optical Coherence Tomography-Based Study. J Coll Physicians Surg Pak. 2016;26(7):598-601. doi: 2379. PubMed PMID: 27504553.

17. Alkuraya H, Kangave D, Abu El-Asrar AM. The correlation between optical coherence tomographic features and severity of retinopathy, macular thickness and visual acuity in diabetic macular edema. Int Ophthalmol. 2005;26(3):93-9. doi: 10.1007/s10792-006-9007-8. PubMed PMID: 17063373.

18. Yang XL, Zou HD, Xu X. [Correlation of retinal sensitivity, visual acuity and central macular thickness in different types of diabetic macular edema] (Article in Chinese). Zhonghua Yan Ke Za Zhi. 2013;49(12):1081-8. PubMed PMID: 24499694.

19. Joltikov KA, Sesi CA, de Castro VM, Davila JR, Anand R, Khan SM, et al. Disorganization of Retinal Inner Layers (DRIL) and Neuroretinal Dysfunction in Early Diabetic Retinopathy. Invest Ophthalmol Vis Sci. 2018;59(13):5481-5486. doi: 10.1167/iovs.18-24955. PubMed PMID: 30452602; PMCID: PMC6735648.

20. Sun JK, Lin MM, Lammer J, Prager S, Sarangi R, Silva PS, et al. Disorganization of the retinal inner layers as a predictor of visual acuity in eyes with center-involved diabetic macular edema. JAMA Ophthalmol. 2014;132(11):1309-16. doi: 10.1001/jamaophthalmol.2014.2350. PubMed PMID: 25058813.

21. Browning DJ, Apte RS, Bressler SB, Chalam KV, Danis RP, Davis MD, et al. Association of the extent of diabetic macular edema as assessed by optical coherence tomography with visual acuity and retinal outcome variables. Retina. 2009;29(3):300-5. doi: 10.1097/IAE.0b013e318194995d. PubMed PMID: 19174719; PMCID: PMC2657814.

22. Boyer DS, Yoon YH, Belfort R, Jr., Bandello F, Maturi RK, Augustin AJ, et al. Three-year, randomized, sham-controlled trial of dexamethasone intravitreal implant in patients with diabetic macular edema. Ophthalmology. 2014;121(10):1904-14. doi: 10.1016/j.ophtha.2014.04.024. PubMed PMID: 24907062.

23. Campochiaro PA, Brown DM, Pearson A, Ciulla T, Boyer D, Holz FG, et al. Long-term benefit of sustained-delivery fluocinolone acetonide vitreous inserts for diabetic macular edema. Ophthalmology. 2011;118(4):626-635.e2. doi: 10.1016/j.ophtha.2010.12.028. PubMed PMID: 21459216.

24. Browning DJ, Fraser CM, Powers ME. Comparison of the magnitude and time course of macular thinning induced by different interventions for diabetic macular edema: implications for sequence of application. Ophthalmology. 2006;113(10):1713-9. doi: 10.1016/j.ophtha.2006.05.021. PubMed PMID: 16889833.

25. Diabetic Retinopathy Clinical Research Network. A randomized trial comparing intravitreal triamcinolone acetonide and focal/grid photocoagulation for diabetic macular edema. Ophthalmology. 2008;115(9):1447-9, 1449.e1-10. doi: 10.1016/j.ophtha.2008.06.015. PubMed PMID: 18662829; PMCID: PMC2748264.

26. Shahidi M, Ogura Y, Blair NP, Zeimer R. Retinal thickness change after focal laser treatment of diabetic macular oedema. Br J Ophthalmol. 1994;78(11):827-30. doi: 10.1136/bjo.78.11.827. PubMed PMID: 7848977; PMCID: PMC504965.

27. Peng YJ, Tsai MJ. Impact of metabolic control on macular thickness in diabetic macular oedema. Diab Vasc Dis Res. 2018;15(2):165-168. doi: 10.1177/1479164117746023. PubMed PMID: 29212365; PMCID: PMC5818022.

28. Wong WM, Chee C, Bhargava M, Chai C, Lin H, Zhao P, et al. Systemic Factors Associated with Treatment Response in Diabetic Macular Edema. J Ophthalmol. 2020;2020:1875860. doi: 10.1155/2020/1875860. PubMed PMID: 32280516; PMCID: PMC7125481.

29. Li B, Zhang B, Chen Y, Li D. Optical Coherence Tomography Parameters Related to Vision Impairment in Patients with Diabetic Macular Edema: A Quantitative Correlation Analysis. J Ophthalmol. 2020;2020:5639284. doi: 10.1155/2020/5639284. PubMed PMID: 33062313; PMCID: PMC7537709.

30. Hatef E, Colantuoni E, Wang J, Ibrahim M, Shulman M, Adhi F, et al. The relationship between macular sensitivity and retinal thickness in eyes with diabetic macular edema. Am J Ophthalmol. 2011;152(3):400-405.e2. doi: 10.1016/j.ajo.2011.02.024. PubMed PMID: 21696702.

31. Diabetic Retinopathy Clinical Research Network, Wells JA, Glassman AR, Ayala AR, Jampol LM, Aiello LP, et al. Aflibercept, bevacizumab, or ranibizumab for diabetic macular edema. N Engl J Med. 2015;372(13):1193-203. doi: 10.1056/NEJMoa1414264. PubMed PMID: 25692915; PMCID: PMC4422053.

32. Lin CM, Titchenell PM, Keil JM, Garcia-Ocaña A, Bolinger MT, Abcouwer SF, et al. Inhibition of Atypical Protein Kinase C Reduces Inflammation-Induced Retinal Vascular Permeability. Am J Pathol. 2018;188(10):2392-2405. doi: 10.1016/j.ajpath.2018.06.020. PubMed PMID: 30220554; PMCID: PMC6180272.

33. Clermont A, Chilcote TJ, Kita T, Liu J, Riva P, Sinha S, et al. Plasma kallikrein mediates retinal vascular dysfunction and induces retinal thickening in diabetic rats. Diabetes. 2011;60(5):1590-8. doi: 10.2337/db10-1260. PubMed PMID: 21444925; PMCID: PMC3292335.

34. Kita T, Clermont AC, Murugesan N, Zhou Q, Fujisawa K, Ishibashi T, et al. Plasma Kallikrein-Kinin System as a VEGF-Independent Mediator of Diabetic Macular Edema. Diabetes. 2015;64(10):3588-99. doi: 10.2337/db15-0317. PubMed PMID: 25979073; PMCID: PMC4587649.

35. Barber AJ, Antonetti DA, Kern TS, Reiter CE, Soans RS, Krady JK, et al. The Ins2Akita mouse as a model of early retinal complications in diabetes. Investigative ophthalmology & visual science. 2005;46(6):2210-2218. doi: 10.1167/iovs.04-1340. PubMed PMID: 15914643.

36. Francis AW, Wanek J, Shahidi M. Assessment of Global and Local Alterations in Retinal Layer Thickness in Ins2 (Akita) Diabetic Mice by Spectral Domain Optical Coherence Tomography. J Ophthalmol. 2018;2018:7253498. doi: 10.1155/2018/7253498. PubMed PMID: 29675273; PMCID: PMC5838457.

37. Ruebsam A, Dulle JE, Myers AM, Sakrikar D, Green KM, Khan NW, et al. A specific phosphorylation regulates the protective role of αA-crystallin in diabetes. JCI Insight. 2018;3(4). doi: 10.1172/jci.insight.97919. PubMed PMID: 29467334; PMCID: PMC5916248.

38. Sohn EH, van Dijk HW, Jiao C, Kok PH, Jeong W, Demirkaya N, et al. Retinal neurodegeneration may precede microvascular changes characteristic of diabetic retinopathy in diabetes mellitus. Proc Natl Acad Sci U S A. 2016;113(19):E2655-64. doi: 10.1073/pnas.1522014113. PubMed PMID: 27114552; PMCID: PMC4868487.

39. Rakoczy EP, Ali Rahman IS, Binz N, Li CR, Vagaja NN, de Pinho M, et al. Characterization of a mouse model of hyperglycemia and retinal neovascularization. Am J Pathol. 2010;177(5):2659-70. doi: 10.2353/ajpath.2010.090883. PubMed PMID: 20829433; PMCID: PMC2966820.

40. Liu Y, Shen J, Fortmann SD, Wang J, Vestweber D, Campochiaro PA. Reversible retinal vessel closure from VEGF-induced leukocyte plugging. JCI Insight. 2017;2(18). doi: 10.1172/jci.insight.95530. PubMed PMID: 28931763; PMCID: PMC5621911.

41. Piazza M, Munasinghe J, Murayi R, Edwards N, Montgomery B, Walbridge S, et al. Simulating vasogenic brain edema using chronic VEGF infusion. J Neurosurg. 2017;127(4):905-916. doi: 10.3171/2016.9.Jns1627. PubMed PMID: 28059647; PMCID: PMC5542877.

42. Motohashi Y, Kemmochi Y, Maekawa T, Tadaki H, Sasase T, Tanaka Y, et al. Diabetic macular edema-like ocular lesions in male spontaneously diabetic torii fatty rats. Physiol Res. 2018;67(3):423-432. doi: 10.33549/physiolres.933709. PubMed PMID: 29527913.

43. Tanaka Y, Takagi R, Ohta T, Sasase T, Kobayashi M, Toyoda F, et al. Pathological Features of Diabetic Retinopathy in Spontaneously Diabetic Torii Fatty Rats. J Diabetes Res. 2019;2019:8724818. doi: 10.1155/2019/8724818. PubMed PMID: 31637263; PMCID: PMC6766157 publication of this paper. Drs. Ohta and Sasase are employees of Japan Tobacco Inc.

44. Hannouche RZ, Avila MP. Retinal thickness measurement and evaluation of natural history of the diabetic macular edema through optical coherence tomography. Arq Bras Oftalmol. 2009;72(4):433-8. doi: 10.1590/s0004-27492009000400002. PubMed PMID: 19820779.

45. Khojasteh H, Riazi-Esfahani H, Khalili Pour E, Faghihi H, Ghassemi F, Bazvand F, et al. Multifocal electroretinogram in diabetic macular edema and its correlation with different optical coherence tomography features. Int Ophthalmol. 2020;40(3):571-581. doi: 10.1007/s10792-019-01215-4. PubMed PMID: 31712927.

46. Xia HH, Chen JL, Chen HY, Lin HJ. Correlation between optical coherence tomography, multifocal electroretinogram findings and visual acuity in diabetic macular edema. Int J Ophthalmol. 2020;13(10):1592-1596. doi: 10.18240/ijo.2020.10.13. PubMed PMID: 33078110; PMCID: PMC7511372.

47. Bressler NM, Beaulieu WT, Maguire MG, Glassman AR, Blinder KJ, Bressler SB, et al. Early Response to Anti-Vascular Endothelial Growth Factor and Two-Year Outcomes Among Eyes With Diabetic Macular Edema in Protocol T. Am J Ophthalmol. 2018;195:93-100. doi: 10.1016/j.ajo.2018.07.030. PubMed PMID: 30077569; PMCID: PMC6648655.

48. Wells JA, Glassman AR, Ayala AR, Jampol LM, Bressler NM, Bressler SB, et al. Aflibercept, Bevacizumab, or Ranibizumab for Diabetic Macular Edema: Two-Year Results from a Comparative Effectiveness Randomized Clinical Trial. Ophthalmology. 2016;123(6):1351-9. doi: 10.1016/j.ophtha.2016.02.022. PubMed PMID: 26935357; PMCID: PMC4877252.

49. Arthur E, Young SB, Elsner AE, Baskaran K, Papay JA, Muller MS, et al. Central Macular Thickness in Diabetic Patients: A Sex-based Analysis. Optom Vis Sci. 2019;96(4):266-275. doi: 10.1097/opx.0000000000001363. PubMed PMID: 30907864; PMCID: PMC6445750.

50. Chalam KV, Bressler SB, Edwards AR, Berger BB, Bressler NM, Glassman AR, et al. Retinal thickness in people with diabetes and minimal or no diabetic retinopathy: Heidelberg Spectralis optical coherence tomography. Invest Ophthalmol Vis Sci. 2012;53(13):8154-61. doi: 10.1167/iovs.12-10290. PubMed PMID: 23132803; PMCID: PMC3522439 Novartis (F), Emmes (F), Notal Vision (F), Regeneron (F), Allergan (F), Abbott Medical Optics (F), Bausch & Lomb (F), Lumenis (F), Forsight (F), Genzyme (F), Alimera Sciences (F), GlaxoSmithKline (F), Notal Vision (F), Novartis (F), Pfizer (F), QLT (F), Quark (F), Diagnos (F), ThromboGenics (F); A.R. Edwards, None; B.B. Berger, Genentech (F, C, R); N.M. Bressler, Carl Zeiss (F), Genentech (F), Novartis (F), Emmes (F), Notal Vision (F), Regeneron (F), Allergan (F), Abbott Medical Optics (F), Bausch & Lomb (F), Lumenis (F), Forsight (F), Genzyme (F), Alimera Sciences (F), GlaxoSmithKline (F), Notal Vision (F), Novartis (F), Pfizer (F), QLT (F); A.R. Glassman, None; S. Grover, None; S.K. Gupta, None; J.S. Nielsen, None.

51. Yoshitake T, Murakami T, Suzuma K, Fujimoto M, Dodo Y, Tsujikawa A. Predictor of Early Remission of Diabetic Macular Edema under As-Needed Intravitreal Ranibizumab. Sci Rep. 2019;9(1):7599. doi: 10.1038/s41598-019-44078-6. PubMed PMID: 31110273; PMCID: PMC6527559.

52. Diabetic Retinopathy Clinical Research Network, Danis RP, Glassman AR, Aiello LP, Antoszyk AN, Beck RW, et al. Diurnal variation in retinal thickening measurement by optical coherence tomography in center-involved diabetic macular edema. Arch Ophthalmol. 2006;124(12):1701-7. doi: 10.1001/archopht.124.12.1701. PubMed PMID: 17159029; PMCID: PMC2279019.

53. Larsen M, Wang M, Sander B. Overnight thickness variation in diabetic macular edema. Invest Ophthalmol Vis Sci. 2005;46(7):2313-6. doi: 10.1167/iovs.04-0893. PubMed PMID: 15980216.

54. Frank RN, Schulz L, Abe K, Iezzi R. Temporal variation in diabetic macular edema measured by optical coherence tomography. Ophthalmology. 2004;111(2):211-7. doi: 10.1016/j.ophtha.2003.05.031. PubMed PMID: 15019364.

55. Browning DJ. Interpreting thickness changes in the diabetic macula: the problem of short-term variation in optical coherence tomography-measured macular thickening (an american ophthalmological society thesis). Trans Am Ophthalmol Soc. 2010;108:62-76. PubMed PMID: 21212849; PMCID: PMC3016084.

56. Browning DJ, Fraser CM, Propst BW. The variation in optical coherence tomography-measured macular thickness in diabetic eyes without clinical macular edema. Am J Ophthalmol. 2008;145(5):889-93. doi: 10.1016/j.ajo.2008.01.007. PubMed PMID: 18329622.

57. Maheshwary AS, Oster SF, Yuson RM, Cheng L, Mojana F, Freeman WR. The association between percent disruption of the photoreceptor inner segment-outer segment junction and visual acuity in diabetic macular edema. Am J Ophthalmol. 2010;150(1):63-67.e1. doi: 10.1016/j.ajo.2010.01.039. PubMed PMID: 20451897; PMCID: PMC2900476.

58. Yohannan J, Bittencourt M, Sepah YJ, Hatef E, Sophie R, Moradi A, et al. Association of retinal sensitivity to integrity of photoreceptor inner/outer segment junction in patients with diabetic macular edema. Ophthalmology. 2013;120(6):1254-61. doi: 10.1016/j.ophtha.2012.12.003. PubMed PMID: 23499060.

59. Beck M, Munk MR, Ebneter A, Wolf S, Zinkernagel MS. Retinal Ganglion Cell Layer Change in Patients Treated With Anti-Vascular Endothelial Growth Factor for Neovascular Age-related Macular Degeneration. Am J Ophthalmol. 2016;167:10-7. doi: 10.1016/j.ajo.2016.04.003. PubMed PMID: 27084000.

60. Shin HJ, Kim SN, Chung H, Kim TE, Kim HC. Intravitreal Anti-Vascular Endothelial Growth Factor Therapy and Retinal Nerve Fiber Layer Loss in Eyes With Age-Related Macular Degeneration: A Meta-Analysis. Invest Ophthalmol Vis Sci. 2016;57(4):1798-806. doi: 10.1167/iovs.15-18404. PubMed PMID: 27077733.

61. Valverde-Megías A, Ruiz-Calvo A, Murciano-Cespedosa A, Hernández-Ruiz S, Martínez-de-la-Casa JM, García-Feijoo J. Long-term effect of intravitreal ranibizumab therapy on retinal nerve fiber layer in eyes with exudative age-related macular degeneration. Graefes Arch Clin Exp Ophthalmol. 2019;257(7):1459-1466. doi: 10.1007/s00417-019-04325-y. PubMed PMID: 31053943.

62. Du J, Patrie JT, Prum BE, Netland PA, Shildkrot YE. Effects of Intravitreal Anti-VEGF Therapy on Glaucoma-like Progression in Susceptible Eyes. J Glaucoma. 2019;28(12):1035-1040. doi: 10.1097/ijg.0000000000001382. PubMed PMID: 31633617.

63. Santos AR, Santos T, Alves D, Marques IP, Lobo C, Cunha-Vaz J. Characterization of Initial Stages of Diabetic Macular Edema. Ophthalmic Res. 2019;62(4):203-210. doi: 10.1159/000499117. PubMed PMID: 31013506.
