# Supplementary material for: A New Approach to Staging Diabetic Eye Disease: Staging of Diabetic Retinal Neurodegeneration and Diabetic Macular Edema
Source: Ophthalmol Sci. 2023 Oct 31;4(3):100420. doi: 10.1016/j.xops.2023.100420 (PMC10818256; doi:10.1016/j.xops.2023.100420)
Supplement: Table S3 [file mmc3.pdf]

**Parameter**	
Parameter name	Full field Flash Electroretinogram (ffERG)
Search terms	Electroretinogram and Diabetes
Search results	661; and human 430; and other animals 279
Pruning	Not full-text and not in English<br>**Human**: not Full field flash ERG 275; not DM or unrelated to DM severity 198; No ERG data 165; other animals 107<br>**Non-human:** Last 5 years 128; not DR model 95; No ERG data 93; methodological 91
How is this parameter assessed?	Most conveniently with RETeval portable device
Has analytical validation been accomplished?<br>If yes, give specifics of evaluation of parameter precision, accuracy, limit of detection, limit of quantitation, specificity, linearity and range, ruggedness and robustness.	**Flicker implicit time (IT)**[1-4]: Intra-subject coefficient of variation (COV) of 5.1%; 8.7% ; ICC up to 0.92; LA 3.0 flicker IT COV 5.3%<br>**b-wave IT**[3,5]: Dark adapted (DA) 0.01 inter-visit repeatability coefficient (RC) 28.8 ms; DA 3.0 RC 7.9 ms; LA 3.0 RC 1.5 ms; LA 3.0 b-wave IT COV 4.0%; DA 3.0 b-wave IT 5.2%; DA 0.01 b-wave IT 7.6%<br>**Oscillatory Potential (OP) IT**[6]: SD 0.6 ms
What kind of variable is this (e.g. a binary event, time to event or quantitative/continuous outcome)?	Continuous; milliseconds from stimulus to response peak.
Are there useful cut points or thresholds for outcome use?	**Flicker IT**[7]: 95th percentile of upper limit of normal (age adjusted)<br>**b-wave IT**[8]: 95th percentile<br>**OP IT**[6]: 95th percentile
**Scientific Understanding of Relationship to DRD**	
What is the biological, anatomic and/or functional rationale or plausibility for the association of this parameter with DRD?<br>(i.e., what is the degree to which diabetes triggers subsequent steps in a pathophysiologic pathway and the role of the parameter in that causal or outcome pathway)	The flicker ERG and photopic b-wave is generated by potassium ion flux between the cone mediated ON bipolar cells and Muller cells. OP changes are attributable to alterations in the connectivity between retinal bipolar cells and amacrine cells
What is the current understanding of the molecular mechanism(s) underlying the alterations in this parameter in association with DRD?<br>(specify whether mechanisms are physiologic, pathologic or pharmacologic)	The implicit timing of the ERG response can be increased by retinal ischemia, by metabolic derangement or by dysfunction in the cone photoreceptor itself.<br>Changes in OP IT have been attributed to decreases in retinal dopamine in diabetic retina or changes in GABA dynamics[9,10]

What is the outcome measure with which this parameter is associated?	An increase in flicker ERG IT is associated with subsequent intervention for DME or PDR[7];<br>OP abnormalities are associated with progression to vision threatening DR[11-14] and to predict onset of DR[15]<br>ERGs have been used to predict VA outcome post-vitrectomy[16,17]
What is the link between the parameter and the accepted clinical outcome measure?	There is a significant positive correlation between Flicker ERG IT and DRSS severity[18]<br>There are significant correlations between DRSS and OP amplitude and OP IT[12]
**Performance Expectations in DRD**	
What sensitivity to detect change does this parameter provide compared to the current standard (if available)?	The combination of flicker ERG IT and DRSS predicts subsequent intervention better than DRSS alone, Bresnick and Palta showed similar sensitivity of OP amplitude to DRSS in prediction of progression [7,19]
Is there consistency of response across species?<br>If yes, please explain	ERG IT delays have been reported in the STZ and db/db murine models of DR[20,21]<br>Pardue et al found similar abnormalities in OP IT in STZ mice and human diabetics[10]
Is there consistency of response across mechanistically or mechanically distinct interventions?<br>If yes, please explain	ERG improvements have been reported with multiple interventions in animal models (See list of non-clinical references with interventions). Most preclinical studies report improvements in amplitude of the a-wave, b-wave, and OPs. A smaller number of preclinical studies report improvements in IT following intervention.<br><br>Clinical studies generally find improvements in amplitude or IT ERG components reflecting inner retinal function (b-wave, OPs, PhNR) without effects on outer retinal function (a-wave). Some studies find that rod system mediated dark-adapted responses are most sensitive to retinal dysfunction in diabetic patients without evidence of DR.
Is there a dose response to the magnitude of changes in this parameter and changes in the clinical outcome?<br>If yes, please give specifics of that relationship	
Is there a temporal relationship between changes in this parameter and the clinical outcome?<br>If yes, please give specifics of that relationship	

What is the specificity of changes in this parameter for DRD?	ERG IT is increased in multiple conditions including ischemia.
**Types of Data and Available for Evidential Evaluation**	
Are there preclinical studies that address the relationship of this parameter to outcomes in DRD?	Yes
If yes, please summarize the available evidence from *in silico* studies	None
References for *in silico* studies	
If yes, please summarize the available evidence from *in vitro* studies	
References for *in vitro* studies	None
If yes, please summarize the available evidence from *in vivo* studies	
References for *in vivo* studies	Many studies have linked ERG IT and amplitude findings with histopathology in murine models of DR and have used ERG as an outcome measure in interventional pre-clinical studies (see references)
Are there clinical studies that address the relationship of this parameter to outcomes in DRD?	Yes
If yes, which of the following clinical study types have been performed: systematic review, prospective randomized controlled trial, retrospective randomized controlled trial, cohort study, case/control study?	retrospective observational trials and prospective RCTs, prospective observational trials
If yes, please summarize the available evidence from clinical studies	In Brigell et al[7], patients with all stages of DR were assessed with flicker ERG and 7-field color fundus photos at baseline and a retrospective look at outcome (intervention for DME or PDR) was assessed over a 3-year follow-up period. For patients with structural evidence of VTDR at baseline, the incidence of intervention was 19%, 31%, and 53% after 1, 2, and 3 years of follow-up. In these patients, intervention incidence increased to 34%, 54%, and 74% the subsequent 1, 2, and 3 years in eyes with increased ERG flicker IT, whereas if flicker IT was below criterion risk was reduced to 3%, 4%, and 29%, respectively; reducing risk to similar levels seen for patients without VTDR at baseline. These results were validated in a prospective RCT in which patients with moderate to severe NPDR without center involved DME were at significantly higher risk of progressing to DME or PDR over the 1-

	year course of the trial regardless of treatment group if their flicker ERG IT was prolonged at baseline.[22]<br>Bresnick et al[11] ,examined progression in 85 diabetic patients participating in the ETDRS study. Those eyes with abnormal OP amplitudes (≤75 µV) at study entry had a tenfold higher rate of progression to high risk than did eyes with normal amplitudes (>75 µV). Although the level of retinopathy severity at study entry was a significant factor in the rate of subsequent progression, the amplitudes of the OPs remained a significant risk factor even after correcting the initial retinopathy level.<br>In a subsequent report on this cohort, Bresnick & Palta[12] report that the probability of progression to severe retinopathy was significantly greater for eyes with the following baseline characteristics: greater overall retinopathy severity, higher fluorescein leakage, higher capillary nonperfusion, and lower electroretinographic oscillatory potential amplitudes. The summed amplitudes of the oscillatory potentials, the overall severity of retinopathy, and the severity of fluorescein angiographic leakage were found to be independent predictors of progression to severe proliferative retinopathy in a regression model.<br>Beneficial effects on OP IT of brimonidine and somatostatin eye drops after 24 months of treatment in patients with diabetic retinopathy have been reported in a clinical trial.[23]<br>Motz et al report reduction in scotopic (rod-mediated) OP IT with low dose L-dopa therapy in diabetic subjects with normal fundi and abnormal OP IT pre-treatment[24]. Unfortunately, this study was not placebo controlled.<br>Biersdorf et al did not find significant effects of Sorbinil (an aldose reductase inhibitor) on the LA b-wave in 19 diabetic patients in a placebo controlled RCT[25].<br>Kim et al reported beneficial effects of 1 year of treatment with cilostazol (an antiplatlet and vasodilatory agent) (n=20) compared to placebo (n=16) on DA 3.0 b-wave IT[26].<br>Nebbioso et al reported increased OP amplitude in 16 diabetics w/o retinopathy treated with anti-oxidants for 1 month compared to 16 patients treated with placebo[27].

	Lessel et al (Ophthalmic Res 1993;25:556-62) failed to find beneficial effects of postacyclins (n-6) compared to placebo (n=6) on any ffERG parameter after 8 months of treatment.<br>Yang et al (Acta Diabetol 2017;54:73-77) found increased amplitude and decreased IT of OPs in 46 diabetic patients treated with Acetagastrodin compared to placebo in a 6 month RCT.<br>Numerous studies have examined the effect of anti-VEGF IVT on the ffERG in subjects with DME with mixed results. Although a number of studies have reported beneficial effects of treatment, no effect, or worsening.[28-32]
References for clinical studies	Included above
Are there literature reviews that address the relationship of this parameter to outcomes in DRD?	
References for literature reviews	Bearse MA Jr, Adams AJ, Han Y, Schneck ME, Ng J, Bronson-Castain K, Barez S. A multifocal electroretinogram model predicting the development of diabetic retinopathy. Prog Retin Eye Res. 2006 Sep;25(5):425-48.<br><br>Frost-Larsen K, Larsen HW, Simonsen SE. Value of electroretinography and dark adaptation as prognostic tools in diabetic retinopathy. Dev Ophthalmol. 1981;2:222-34.<br><br>Pescosolido N, Barbato A, Stefanucci A, Buomprisco G. Role of Electrophysiology in the Early Diagnosis and Follow-Up of Diabetic Retinopathy. J Diabetes Res. 2015;2015:319692.<br><br>Shirao Y, Kawasaki K. Electrical responses from diabetic retina. Prog Retin Eye Res. 1998 Jan;17(1):59-76.<br><br>Speros P, Price J. Oscillatory potentials. History, techniques and potential use in the evaluation of disturbances of retinal circulation. Surv Ophthalmol. 1981 Jan-Feb;25(4):237-52.<br><br>Ting DS, Tan KA, Phua V, Tan GS, Wong CW, Wong TY. Biomarkers of Diabetic Retinopathy. Curr Diab Rep. 2016 Dec;16(12):125.

	Tzekov R, Arden GB. The electroretinogram in diabetic retinopathy. Surv Ophthalmol. 1999 Jul-Aug;44(1):53-60
Please give the Level of Evidence available from these combined studies (use Tables 1 and 2 below to determine Level of Evidence. For this purpose, please substitute "DRD parameter" for "tumor marker" or "marker" in Table 1)	IB/IIC
**Statistical Considerations**	
What is the specific relationship of the parameter to clinical outcomes?<br>Please specify effect sizes and measures of variability	
What is the usefulness of the parameter or its thresholds for clinical or research decision making?	For longitudinal measures of IT an increase of 5 ms or more is clinically significant for the DA 3.0 a-wave, the LA 3.0 a-wave, b-wave, and LA 3.0 flicker. A 10 ms criterion is needed for the DA 0.01 b-wave and the DA 3.0 b-wave IT.[8] Data supports that a 40% or more reduction in amplitude of any component of the ffERG is clinically significant IT.[8,33]<br>Thresholds for abnormality are generally determined by age adjusted 95% CI.[34] Normative data is available on most commercial ERG devices.
Are there covariates that should be adjusted for when considering this parameter?	Age, blood glucose level
Are there any additional statistical considerations for the use of this parameter?	
**Gap Analysis**	
What are the gaps in the literature to prove or disprove the utility of this parameter?	It is unclear which ERG parameter is most sensitive to disease progression, and this may vary with stage of disease. Some literature suggests that Scotopic Oscillatory potential (OP) IT is most sensitive to neural changes in diabetic eyes without evidence of DR (Pardue lab). Historically, Bresnick and colleagues have shown predictive value of Photopic OP IT. Brigell and colleagues have shown sensitivity of the cone ERG IT but have not focused on early stages of the disease.<br>The relationship between flicker ERG IT and other measures of visual function have not yet been established.
In your opinion, what clinical research study/studies could address these gaps?	A longitudinal observational study in patients stratified by DR severity comparing photopic (light adapted) and scotopic (dark-

	adapted) b-wave and OP parameters along with other functional and structural measures
Are there currently available datasets that could be used for these validation efforts?	
**Miscellaneous Questions**	
Is this parameter currently employed in clinical use?	Not widely for DR. Mostly for IRDs
Is assessment instrumentation needed to measure this parameter currently: available commercially, available but not FDA approved, not readily available, or not available?	Currently commercially available. Suggest RETeval (LKC Technologies). Low cost, portable, no pupil dilation required.
What is the ease of implementation in the following environments: high resource academic center, high resource community practice, low resource/underserved environment?	Easily implemented in all settings
What sites are appropriate for this assessment?<br>Indicate all relevant site types: retina clinic, general ophthalmology clinic, optometry clinic, endocrinology clinic, general medical clinic, patient home.	All with the exception of patient home
Is there any technology or advance either currently available, in development, or not yet developed that would make this parameter no longer important or relevant?<br>If yes, please specify what technology or advance	
What unmet need in the staging of DRD does this parameter address?	There is currently no measure of function in the staging of DR
**Summary**	
Based on the above data, please provide an integrated evaluation regarding the overall importance of this parameter to the field currently. If not currently relevant, please summarize the potential for future relevance, necessary steps for validation and a reasonable time frame for this to occur.	Recent research has shown that the flicker ERG IT has comparable predictive value to conventional ETDRS-DRSS and that the combined information has more prognostic value than the results of either parameter alone. Abnormalities in OPs are present in diabetic patients without evidence of retinopathy and are predictive of progression of the disease. ERG b-wave amplitude has been used in most preclinical models of DR. This parameter is easily translated to human, but amplitude measurements have much higher test-retest variability, as well as higher inter-subject variability, and thus are not anticipated to be good biomarkers of disease state. With advances in ERG technology the test can be implemented at a site for minimal cost and the procedure no longer requires pupil dilation, or use of a contact lens electrode,

1. Davis CQ, Kraszewska O, Manning C. Constant luminance (cd·s/m(2)) versus constant retinal illuminance (Td·s) stimulation in flicker ERGs. *Doc Ophthalmol.* 2017;134(2):75-87.
2. Kim BG, Chang IB, Jeong KD, Park JY, Kim JS, Hwang JH. Comparison of electroretinographic measurements between tabletop and handheld stimulators in healthy subjects. *Doc Ophthalmol.* 2019;139(1):1-9.
3. Zrenner E, Holder GE, Schiefer U, Wild JM. Quality Control Procedures and Baseline Values for Electroretinography, Perimetry, Color Vision, and Visual Acuity in an International Multicenter Study: Observations from a Safety Trial in Chronic Stable Angina Pectoris. *Transl Vis Sci Technol.* 2020;9(8):38.
4. Liu H, Ji X, Dhaliwal S, et al. Evaluation of light- and dark-adapted ERGs using a mydriasis-free, portable system: clinical classifications and normative data. *Doc Ophthalmol.* 2018;137(3):169-181.
5. Knickelbein JE, Jeffrey BG, Wei MM, et al. Reproducibility of Full-field Electroretinogram Measurements in Birdshot Chorioretinopathy Patients: An Intra- and Inter-visit Analysis. *Ocul Immunol Inflamm.* 2021;29(5):848-853.
6. Lachapelle P. The human suprathreshold photopic oscillatory potentials: method of analysis and clinical application. *Doc Ophthalmol.* 1994;88(1):1-25.
7. Brigell MG, Chiang B, Maa AY, Davis CQ. Enhancing Risk Assessment in Patients with Diabetic Retinopathy by Combining Measures of Retinal Function and Structure. *Translational vision science & technology.* 2020;9(9):40-40.
8. Grover S, Fishman GA, Birch DG, Locke KG, Rosner B. Variability of full-field electroretinogram responses in subjects without diffuse photoreceptor cell disease. *Ophthalmology.* 2003;110(6):1159-1163.
9. Eggers ED, Carreon TA. The effects of early diabetes on inner retinal neurons. *Vis Neurosci.* 2020;37:E006.
10. Pardue MT, Barnes CS, Kim MK, et al. Rodent Hyperglycemia-Induced Inner Retinal Deficits are Mirrored in Human Diabetes. *Transl Vis Sci Technol.* 2014;3(3):6.
11. Bresnick GH, Korth K, Groo A, Palta M. Electroretinographic oscillatory potentials predict progression of diabetic retinopathy. Preliminary report. *Arch Ophthalmol.* 1984;102(9):1307-1311.
12. Bresnick GH, Palta M. Temporal aspects of the electroretinogram in diabetic retinopathy. *Arch Ophthalmol.* 1987;105(5):660-664.
13. Moschos M, Panagakis E, Angelopoulos A. Changes of oscillatory potentials of the ERG in diabetic retinopathy. *Ophthalmic Physiol Opt.* 1987;7(4):477-479.
14. Simonsen SE. The value of the oscillatory potential in selecting juvenile diabetics at risk of developing proliferative retinopathy. *Metab Pediatr Ophthalmol.* 1981;5(1):55-61.
15. Vadalà M, Anastasi M, Lodato G, Cillino S. Electroretinographic oscillatory potentials in insulin-dependent diabetes patients: A long-term follow-up. *Acta Ophthalmol Scand.* 2002;80(3):305-309.
16. Algvere P, Persson HE, Wanger P. Preoperative electroretinograms and visual evoked cortical potentials for predicting outcome of vitrectomy in diabetics. *Retina.* 1985;5(3):179-183.
17. Summanen P. Vitrectomy for diabetic eye disease. The prognostic value of pre-operative electroretinography and visual evoked cortical potentials. *Ophthalmologica.* 1989;199(2-3):60-71.
18. Maa AY, Feuer WJ, Davis CQ, et al. A novel device for accurate and efficient testing for vision-threatening diabetic retinopathy. *Journal of Diabetes and its Complications.* 2016;30(3):524-532.
19. Bresnick GH, Palta M. Predicting progression to severe proliferative diabetic retinopathy. *Arch Ophthalmol.* 1987;105(6):810-814.
20. Hernández C, Bogdanov P, Gómez-Guerrero C, et al. SOCS1-Derived Peptide Administered by Eye Drops Prevents Retinal Neuroinflammation and Vascular Leakage in Experimental Diabetes. *International journal of molecular sciences.* 2019;20(15):3615.

21. Naderi A, Zahed R, Aghajanpour L, Amoli FA, Lashay A. Long term features of diabetic retinopathy in streptozotocin-induced diabetic Wistar rats. *Exp Eye Res.* 2019;184:213-220.
22. Brigell MG, Davis Q, Waheed NK. Predictive Value of ERG, OCT-A, and UWF-FA in Patients with Diabetic Retinopathy [ARVO Annual Meeting Abstract]. *Investigative Ophthalmology & Visual Science.* 2020;61(7):4038.
23. Simó R, Hernández C, Porta M, et al. Effects of topically administered neuroprotective drugs in early stages of diabetic retinopathy: results of the EUROCONDOR clinical trial. *Diabetes.* 2019;68(2):457-463.
24. Motz CT, Chesler KC, Allen RS, et al. Novel detection and restorative levodopa treatment for preclinical diabetic retinopathy. *Diabetes.* 2020;69(7):1518-1527.
25. Biersdorf WR, Malone JI, Pavan PR, Lowitt S. Cone electroretinograms and visual acuities of diabetic patients on sorbinil treatment. *Doc Ophthalmol.* 1988;69(3):247-254.
26. Kim HD, Lee SH, Kim YK, Oh JR, Ohn YH. The effect of cilostazol on electrophysiologic changes in non-proliferative diabetic retinopathy patients. *Doc Ophthalmol.* 2016;133(1):49-60.
27. Nebbioso M, Federici M, Rusciano D, Evangelista M, Pescosolido N. Oxidative stress in preretinopathic diabetes subjects and antioxidants. *Diabetes Technol Ther.* 2012;14(3):257-263.
28. Holm K, Schroeder M, Lövestam Adrian M. Peripheral retinal function assessed with 30-Hz flicker seems to improve after treatment with Lucentis in patients with diabetic macular oedema. *Doc Ophthalmol.* 2015;131(1):43-51.
29. Yigit K, Inan Ü, Inan S, Dogan M, Yavas GF, Cetinkaya E. Long-term full-field and multifocal electroretinographic changes after treatment with ranibizumab in patients with diabetic macular edema. *Int Ophthalmol.* 2021;41(4):1487-1501.
30. Comyn O, Sivaprasad S, Peto T, et al. A randomized trial to assess functional and structural effects of ranibizumab versus laser in diabetic macular edema (the LUCIDATE study). *Am J Ophthalmol.* 2014;157(5):960-970.
31. Ichio A, Sugimoto M, Matsubara H, Mochida D, Kato K, Kondo M. Effects of Intravitreal Aflibercept on Retinal Function and Improvement of Macular Edema Associated With Diabetic Retinopathy. *Transl Vis Sci Technol.* 2020;9(11):2.
32. Terauchi G, Shinoda K, Sakai H, et al. Retinal function determined by flicker ERGs before and soon after intravitreal injection of anti-VEGF agents. *BMC Ophthalmol.* 2019;19(1):129.
33. Cordell WH, Maturi RK, Costigan TM, et al. Retinal effects of 6 months of daily use of tadalafil or sildenafil. *Arch Ophthalmol.* 2009;127(4):367-373.
34. Birch DG, Anderson JL. Standardized full-field electroretinography. Normal values and their variation with age. *Arch Ophthalmol.* 1992;110(11):1571-1576.
