# Supplementary material for: A New Approach to Staging Diabetic Eye Disease: Staging of Diabetic Retinal Neurodegeneration and Diabetic Macular Edema
Source: Ophthalmol Sci. 2023 Oct 31;4(3):100420. doi: 10.1016/j.xops.2023.100420 (PMC10818256; doi:10.1016/j.xops.2023.100420)
Supplement: Table S4 [file mmc4.pdf]

**Parameter**	
Parameter name	Retinal sensitivity
Search terms	"microperimetry and diabetes", "microperimetry and diabetic retinopathy" and "microperimetry and retinal neurodegeneration" from inception to 3/9/2021
Search results	330 articles
Pruning	We excluded repeated publications, letters, conference abstracts and those papers that were not relevant for the purpose of the study. A total of 38 articles in which full-text was available in English were reviewed. From these 38, 12 were excluded due to problems with the design of the study or absence of valuable information to answer the questions in the grid.
How is this parameter assessed?	Retinal sensitivity is assessed by microperimetry. MAIA microperimetry, similarly to standard automated perimetry (SAP), measures retinal sensitivity as the minimum light intensity that patients can perceive when spots of light stimulate specific areas of the retina.<br>The examination can be customized with different number of stimuli covering a variable field of vision. The standard MAIA examination covers a 10° diameter area with 37 measurement points. In MAIA, light stimuli are created by a white LED and projected directly onto the retina surface. The stimuli size are Goldmann III, background luminance is 4 asb and maximum luminance is 1000 asb, with a 36 decibels (dB) dynamic range.<br>MAIA can work with 3 different projection strategies (software version 1.7.0, January 2013); the full threshold 4-2, the 4 Levels Fixed (4-LF) and the Scotoma-Finder (SF). In our group we have used the second strategy [1, 2].
Has analytical validation been accomplished?<br>If yes, give specifics of evaluation of parameter precision, accuracy, limit of detection, limit of quantitation, specificity, linearity and range, ruggedness and robustness.	Yes, but it is not currently used to measure retinal neurodysfunction in DR.<br>In eyes with AMD, it has been previously found the point-wise coefficient of repeatability of microperimetry (representing the location where 95% of the test–retest differences are expected to lie) to be ±4.37 dB or less [3].<br><u>*Factors that should be taken into account*</u>:<br>• *Pupil dilation*: pupil dilation does not significantly influence microperimetry performance [4].<br>• *Adaptation*: mesopic adaptation can influence microperimetry performance. For this purpose, 20 min of mesopic adaptation prior to testing is required.
What kind of variable is this (e.g. a binary event, time to event or quantitative/continuous outcome)?	Continuous
Are there useful cut points or thresholds for outcome use?	There is a lack of consensus regarding the normal values. This is because the different devices used and the characteristics of subjects included. Please see below the most important series:<br>• *Midena et al. Macular automatic fundus threshold versus standard perimetry threshold. Eur J Ophthalmol. 2007;17:63–8. [5]*

	• *Shah et al. Values for macular perimetry using the MP-1 microperimeter in normal subjects. Ophthalmic Res. 2009;41:9–13. [6]*<br>• *Sabates et al. Normative data set identifying properties of the macula across age groups: integration of visual function and retinal structure with microperimetry and spectral-domain optical coherence tomography. Retina. 2011;31:1294-302. [7]*<br>• *Gella et al. Indian J Ophthalmol. 2014; 62: 284–286. [8]*<br>• *Molina-Martín et al. Normal values for microperimetry with the MAIA microperimeter: sensitivity and fixation analysis in healthy adults and children. Eur J Ophthalmol. 2017;27:607-613. [9]*<br><br>The values mainly depend on the following: age, device, the central degrees of the macula analyzed, and the number of stimulus points used.
**Scientific Understanding of Relationship to DRD**	
What is the biological, anatomic and/or functional rationale or plausibility for the association of this parameter with DRD?<br>(i.e., what is the degree to which diabetes triggers subsequent steps in a pathophysiologic pathway and the role of the parameter in that causal or outcome pathway)	Retinal neurodysfunction and neurodegeneration are early events in the pathogenesis of DRD.
What is the current understanding of the molecular mechanism(s) underlying the alterations in this parameter in association with DRD?<br>(specify whether mechanisms are physiologic, pathologic or pharmacologic)	The reason by which there is a reduction of retinal sensitivity in the diabetic retina could be primarily due to the neural impairment/neural loss as has been reported in aging [10]. It should be noted that a total reduction of 27 µm in retinal thickness results in approximately 1 dB of sensitivity loss in subjects with mild NPDR [11].<br>In addition, it has been reported that retinal sensitivity is correlated to GCL-IPL thickness in diabetic subjects [12] and ganglion cell count [13].<br><br>The following confounding factors should be considered in clinical practice:<br><br>Macular edema: In patients with DME a decrease of 0.83 dB for every 10% of deviation of retinal thickness from normal values has been reported [14]. Macular edema could cause light to be blocked or scattered before it reaches the photoreceptors, suggesting that optical effects are a major cause of sensitivity loss. In addition, it has been recently reported a direct relationship between retinal sensitivity and macular thickness in the DM1 group [15].<br>Nonperfused areas: Areas of capillary nonperfusion resulting from severe nonproliferative or proliferative diabetic retinopathy show morphologic changes of the retinal structure, which may

	lead to a loss of sensitivity [16]. In addition, retinal sensitivity correlated inversely with superficial foveal avascular zone area measured by optical coherence tomography angiography (OCTA) [17].<br><br>Cognitive impairment: Retinal sensitivity has been found decreased in those patients with mild cognitive impairment and dementia [1]. Therefore, cognitive status should be taken into account in the interpretation of the results or as exclusion criteria in clinical trials.
What is the outcome measure with which this parameter is associated?	Retinal neurodysfunction
What is the link between the parameter and the accepted clinical outcome measure?	Retinal sensitivity is a measurement of retinal neurodysfunction/neurodegneneration
**Performance Expectations in DRD**	
What sensitivity to detect change does this parameter provide compared to the current standard (if available)?	The current gold standard to assess neurodysfunction is mfERG which is an objective measurement of suprathreshold responses at photopic adaptation levels and the first-order kernel responses originate from the cone photoreceptors and bipolar cells [18]. However, this examination is cumbersome and time consuming and, therefore, it is reserved for clinical trials.<br>Microperimetry is a measurement of retinal sensitivity at mesopic adaptation levels, which may be mediated by both rod and cone photoreceptors. Measurements of retinal sensitivity are also not solely influenced by the physiological condition of the retina, but by the entire visual pathway. Retinal sensitivity measured by microperimetry has been found to correlate strongly with the integrity of the photoreceptor band on high-resolution OCT imaging in AMD [19, 20].<br><br>It should be noted that no significant correlation between the measured functional deficit of microperimetric retinal sensitivity and mfERG implicit time nor response amplitude was observed in AMD subjects [21]. This lack of correlation could be explained because they are giving us different information about retinal function and are measured under different conditions. It has been reported that microperimetry is even more sensitive that mfERG at detecting early functional changes [22] and changes in response to treatment [23].<br><br>In comparison with mfERG, microperimetry is a noninvasive and rapid test, with the total procedural time for each test in our study lasting for approximately 6 to 7 minutes. This contrasts with the total procedural time of at least 15 to 20 minutes for mfERG recording of one eye.
Is there consistency of response across species?<br>If yes, please explain	N/A
Is there consistency of response across mechanistically or mechanically distinct interventions?<br>If yes, please explain	N/A

Is there a dose response to the magnitude of changes in this parameter and changes in the clinical outcome?<br>If yes, please give specifics of that relationship	The reported experience on this issue is very limited.<br>In early stages of DR, supplementation with high-dose DHA plus xanthophyll carotenoid multivitamin during 90 days was associated with a progressive and significant improvement of macular function measured by microperimetry (macular sensitivity increased from 25.9 ± 2.4 dB at baseline to 27.3± 2.3 dB in the DHA group; p <0.05) [24].<br>In advanced stages of DR, microperimetry retinal sensitivity was correlated with visual response in patients treated with undergoing intravitreal ranibizumab for DME. Thus, in good responders the mean intra-subject improvement after month 3 of treatment was 2.28 dB (p=0.049), whereas in poor responders was 1.07 db (p=0.28) [25].
Is there a temporal relationship between changes in this parameter and the clinical outcome?<br>If yes, please give specifics of that relationship	Impairment of retinal sensitivity is already the outcome (neurodysfunction). If we consider the clinical outcome microvascular progression there is a lack of experience.
What is the specificity of changes in this parameter for DRD?	It seems a very high specific method. However, the presence of cataracts [26], vascular leakage and cognitive impairment are potential confounding factors that should be considered.
**Types of Data and Available for Evidential Evaluation**	
Are there preclinical studies that address the relationship of this parameter to outcomes in DRD?	N/A
If yes, please summarize the available evidence from *in silico* studies	
References for *in silico* studies	
If yes, please summarize the available evidence from *in vitro* studies	
References for *in vitro* studies	
If yes, please summarize the available evidence from *in vivo* studies	
References for *in vivo* studies	
Are there clinical studies that address the relationship of this parameter to outcomes in DRD?	Please see above [ref. 24]
If yes, which of the following clinical study types have been performed: systematic review, prospective randomized controlled trial,	Prospective randomized controlled trial [24]

retrospective randomized controlled trial, cohort study, case/control study?	
If yes, please summarize the available evidence from clinical studies	
References for clinical studies	Please see above [ref. 24]
Are there literature reviews that address the relationship of this parameter to outcomes in DRD?	
References for literature reviews	N/A
Please give the Level of Evidence available from these combined studies (use Tables 1 and 2 below to determine Level of Evidence. For this purpose, please substitute "DRD parameter" for "tumor marker" or "marker" in Table 1)	
**Statistical Considerations**	
What is the specific relationship of the parameter to clinical outcomes?<br>Please specify effect sizes and measures of variability	This is a parameter of neurodysfunction.<br><br>This point should be explored.
What is the usefulness of the parameter or its thresholds for clinical or research decision making?	A large study in order to obtain a normative data base is needed. This will be crucial to standardize the presence of neurodysfunction and monitoring any intervention that could have an impact on neurodysfunction.
Are there covariates that should be adjusted for when considering this parameter?	Age. It has been reported a loss in visual field sensitivity with increasing age (approximately 0.8 dB per decade) by Humphrey Field Analyzer [10].
Are there any additional statistical considerations for the use of this parameter?	No
**Gap Analysis**	
What are the gaps in the literature to prove or disprove the utility of this parameter?	The main gaps are: 1) Lack of standardization. 2) Altered values in patients with cognitive impairment*<br><br>*There is evidence that retinal sensitivity assessed by microperimetry is able to discriminate normocognition, mild cognitive impairment and dementia in subjects with type 2 diabetes. In addition, a significant correlation was found between retinal sensitivity and the MRI and 18FDG-PET parameters related to brain neurodegeneration [1]. This is because, fundus-driven microperimetry assesses not only the functional status of the retina but also the entire visual

	system, and it is a dynamic test that requires short-term memory and adequate perceptual speed and executive function. Moreover, the impairment of gaze fixation (which can also be assessed by microperimetry) is strongly related with cognitive decline [2].
In your opinion, what clinical research study/studies could address these gaps?	A large study in order to obtain a normative data base is needed.
Are there currently available datasets that could be used for these validation efforts?	A large prospective study (RECOGNISED. H2020. Grant agreement: 847749) is ongoing. In this study the correlation between retinal sensibility measured by MAIA and other parameters of DRD will be evaluated in type 2 diabetic patients.
**Miscellaneous Questions**	
Is this parameter currently employed in clinical use?	Yes, but it has not been generally used for the assessment of neurodysfunction in the setting of DR.
Is assessment instrumentation needed to measure this parameter currently: available commercially, available but not FDA approved, not readily available, or not available?	Available commercially
What is the ease of implementation in the following environments: high resource academic center, high resource community practice, low resource/underserved environment?	High resource academic center and high resource community practice. However, this could be extended to low-income countries when the cost would be reduced due to competitiveness among the companies.
What sites are appropriate for this assessment?<br>Indicate all relevant site types: retina clinic, general ophthalmology clinic, optometry clinic, endocrinology clinic, general medical clinic, patient home.	Clinical sites, in particular in diabetic units and/or in the ophthalmology clinic
Is there any technology or advance either currently available, in development, or not yet developed that would make this parameter no longer important or relevant?<br>If yes, please specify what technology or advance	No
What unmet need in the staging of DRD does this parameter address?	Screening of neurodysfunction.
**Summary**	
Based on the above data, please provide an integrated evaluation regarding the overall importance of this parameter to the field currently. If not currently relevant, please summarize the	Retinal neurodysfunction and/or neurodegeneration are early events in the pathogenesis of DRD. Retinal sensitivity, assessed by microperimetry is currently used to measure retinal neurodysfunction in AMD or retinitis pigmentosa, but not in the setting of DRD.

potential for future relevance, necessary steps for validation and a reasonable time frame for this to occur.	The reason by which there is a reduction of retinal sensitivity in the diabetic retina could be primarily due to the neural impairment/neural loss as has been reported in aging.<br>Overall, retinal sensitivity assessed by microperimetry seems a very high specific method. However, the presence of cataracts, vascular leakage and cognitive impairment are potential confounding factors that should be considered. Among them, the presence of cognitive impairment represents a significant limiting factor in patients >65 years old.<br>The current gold standard to assess neurodysfunction in diabetic patients is mfERG. However, this examination is cumbersome and time consuming and, therefore, it is reserved for clinical trials. In comparison with mfERG, microperimetry is a noninvasive and rapid test, with the total procedural time for each test lasting for approximately 6 to 7 minutes. This contrasts with the total procedural time of at least 15 to 20 minutes for mfERG recording of one eye.<br><br>The reported clinical experience on retinal sensitivity to evaluate neurodysfunction due to diabetes is very limited. However, in early stages of DR, supplementation with high-dose DHA plus xanthophyll carotenoid multivitamin was associated with a progressive and significant improvement of macular function measured by microperimetry.<br><br>There is a lack of consensus regarding the normal values of retinal sensitivity that mainly depend on the following variables: age, type of device, central degrees of the macula analyzed and the number of stimulus points used. Therefore, a large study in order to obtain a normative data base is needed. This will be crucial to standardize the screening of neurodysfunction and monitoring any intervention that could have an impact on neurodysfunction/neurodegeneration.<br><br>It is unknown whether neurodysfunction assessed by microperiemtry is a predictor of microvascular impairment. A large prospective study (RECOGNISED. H2020. Grant agreement: 847749) is ongoing. In this study, the correlation between retinal sensibility measured by MAIA and other microvascular parameters of DRD will be evaluated in type 2 diabetic patients. In addition, the RECOGNISED clinical trial will determine whether retinal sensitivity is a predictor of cognitive decline and dementia. This study will be finished in 2024.

References

1. Ciudin A, Simó-Servat O, Hernández C, Arcos G, Diego S, Sanabria Á, Sotolongo Ó, Hernández I, Boada M, Simó R. Retinal Microperimetry: A New Tool for Identifying Patients With Type 2 Diabetes at Risk for Developing Alzheimer Disease. Diabetes. 2017 Dec;66(12):3098-3104. doi: 10.2337/db17-0382. Epub 2017 Sep 26. PMID: 28951388.
2. Simó-Servat O, Ciudin A, Ortiz-Zúñiga ÁM, Hernández C, Simó R. Usefulness of Eye Fixation Assessment for Identifying Type 2 Diabetic Subjects at Risk of Dementia. J Clin Med. 2019 Jan 8;8(1):59. doi: 10.3390/jcm8010059. PMID: 30626106.
3. Wu Z, Ayton LN, Guymer RH, Luu CD. Intrasession test-retest variability of microperimetry in age-related macular degeneration. Invest Ophthalmol Vis Sci. 2013 Nov 11;54(12):7378-85. doi: 10.1167/iovs.13-12617. PMID: 24135753.

4. Han RC, Jolly JK, Xue K, MacLaren RE. Effects of pupil dilation on MAIA microperimetry. Clin Exp Ophthalmol. 2017 Jul;45(5):489-495. doi: 10.1111/ceo.12907. Epub 2017 Jan 24. PMID: 28002873.
5. Midena E, Radin PP, Convento E, Cavarzeran F. Macular automatic fundus perimetry threshold versus standard perimetry threshold. Eur J Ophthalmol. 2007 Jan-Feb;17(1):63-8. doi: 10.1177/112067210701700109. PMID: 17294384.
6. Shah VA, Chalam KV. Values for macular perimetry using the MP-1 microperimeter in normal subjects. Ophthalmic Res. 2009;41(1):9-13. doi: 10.1159/000162111. Epub 2008 Oct 13. PMID: 18849636.
7. Sabates FN, Vincent RD, Koulen P, Sabates NR, Gallimore G. Normative data set identifying properties of the macula across age groups: integration of visual function and retinal structure with microperimetry and spectral-domain optical coherence tomography. Retina. 2011 Jul-Aug;31(7):1294-302. doi: 10.1097/IAE.0b013e3182019be2. PMID: 21358460.
8. Gella L, Nittala MG, Raman R. Retinal sensitivity in healthy Indians using microperimeter. Indian J Ophthalmol. 2014 Mar;62(3):284-6. doi: 10.4103/0301-4738.111211. PMID: 23619501.
9. Molina-Martín A, Piñero DP, Pérez-Cambrodí RJ. Normal values for microperimetry with the MAIA microperimeter: sensitivity and fixation analysis in healthy adults and children. Eur J Ophthalmol. 2017 Aug 30;27(5):607-613. doi: 10.5301/ejo.5000930. Epub 2017 Jan 23. PMID: 28127734.
10. Johnson CA, Adams AJ, Lewis RA. Evidence for a neural basis of age-related visual field loss in normal observers. Invest Ophthalmol Vis Sci. 1989 Sep;30(9):2056-64. PMID: 2777523.
11. Park JC, Chen YF, Liu M, Liu K, McAnany JJ. Structural and Functional Abnormalities in Early-stage Diabetic Retinopathy. Curr Eye Res. 2020 Aug;45(8):975-985. doi: 10.1080/02713683.2019.1705983. Epub 2020 Jan 10. PMID: 31847599.
12. Montesano G, Gervasoni A, Ferri P, Allegrini D, Migliavacca L, De Cillà S, Rossetti L. Structure-function relationship in early diabetic retinopathy: a spatial correlation analysis with OCT and microperimetry. Eye (Lond). 2017 Jun;31(6):931-939. doi: 10.1038/eye.2017.27. Epub 2017 Mar 3. PMID: 28257130; PMCID: PMC5518836.
13. Young IS, Kee F, Wright DM, Crabb DP, Hogg RE. Evidence for Structural and Functional Damage of the Inner Retina in Diabetes With No Diabetic Retinopathy. Invest Ophthalmol Vis Sci. 2021 Mar 1;62(3):35. doi: 10.1167/iovs.62.3.35. PMID: 33760040.
14. Vujosevic S, Midena E, Pilotto E, Radin PP, Chiesa L, Cavarzeran F. Diabetic macular edema: correlation between microperimetry and optical coherence tomography findings. Invest Ophthalmol Vis Sci. 2006 Jul;47(7):3044-51. doi: 10.1167/iovs.05-1141. PMID: 16799051.
15. Orduna-Hospital E, Otero-Rodríguez J, Perdices L, Sánchez-Cano A, Boned-Murillo A, Acha J, Pinilla I. Microperimetry and Optical Coherence Tomography Changes in Type-1 Diabetes Mellitus without Retinopathy. Diagnostics (Basel). 2021 Jan 16;11(1):136. doi: 10.3390/diagnostics11010136. PMID: 33467213.
16. Unoki N, Nishijima K, Sakamoto A, Kita M, Watanabe D, Hangai M, Kimura T, Kawagoe N, Ohta M, Yoshimura N. Retinal sensitivity loss and structural disturbance in areas of capillary nonperfusion of eyes with diabetic retinopathy. Am J Ophthalmol. 2007 Nov;144(5):755-760. doi: 10.1016/j.ajo.2007.07.011. Epub 2007 Sep 14. PMID: 17868632.
17. Tsai ASH, Gan ATL, Ting DSW, Wong CW, Teo KYC, Tan ACS, Lee SY, Wong TY, Tan GSW, Gemmy Cheung CM. DIABETIC MACULAR ISCHEMIA: Correlation of Retinal Vasculature Changes by Optical Coherence Tomography Angiography and Functional Deficit. Retina. 2020 Nov;40(11):2184-2190. doi: 10.1097/IAE.0000000000002721. PMID: 31842192.
18. Hood DC, Frishman LJ, Saszik S, Viswanathan S. Retinal origins of the primate multifocal ERG: implications for the human response. Invest Ophthalmol Vis Sci. 2002 May;43(5):1673-85. PMID: 11980890.
19. Hartmann KI, Bartsch DU, Cheng L, Kim JS, Gomez ML, Klein H, Freeman WR. Scanning laser ophthalmoscope imaging stabilized microperimetry in dry age-related macular degeneration. Retina. 2011 Jul-Aug;31(7):1323-31. doi: 10.1097/IAE.0b013e31820a6850. PMID: 21540764.
20. Landa G, Su E, Garcia PM, Seiple WH, Rosen RB. Inner segment-outer segment junctional layer integrity and corresponding retinal sensitivity in dry and wet forms of age-related macular degeneration. Retina. 2011 Feb;31(2):364-70. doi: 10.1097/IAE.0b013e3181e91132. PMID: 21221051.
21. Wu Z, Ayton LN, Guymer RH, Luu CD. Comparison between multifocal electroretinography and microperimetry in age-related macular degeneration. Invest Ophthalmol Vis Sci. 2014 Aug 26;55(10):6431-9. doi: 10.1167/iovs.14-14407. PMID: 25159206.

22. Jivrajka RV, Genead MA, McAnany JJ, Chow CC, Mieler WF. Microperimetric sensitivity in patients on hydroxychloroquine (Plaquenil) therapy. Eye (Lond). 2013 Sep;27(9):1044-52. doi: 10.1038/eye.2013.112. Epub 2013 Jun 14. PMID: 23764990.
23. Querques G, Lattanzio R, Querques L, Triolo G, Cascavilla ML, Cavallero E, Del Turco C, Casalino G, Bandello F. Impact of intravitreal dexamethasone implant (Ozurdex) on macular morphology and function. Retina. 2014 Feb;34(2):330-41. doi: 10.1097/IAE.0b013e31829f7495. PMID: 23945638.
24. Rodríguez González-Herrero ME, Ruiz M, López Román FJ, Marín Sánchez JM, Domingo JC. Supplementation with a highly concentrated docosahexaenoic acid plus xanthophyll carotenoid multivitamin in nonproliferative diabetic retinopathy: prospective controlled study of macular function by fundus microperimetry. Clin Ophthalmol. 2018 May 29;12:1011-1020. doi: 10.2147/OPTH.S157635. PMID: 29881256; PMCID: PMC5983010.
25. Palkovits S, Hirnschall N, Georgiev S, Leisser C, Findl O. Effect of cataract extraction on retinal sensitivity measurements. Ophthalmic Res. 2020 Mar 25. doi: 10.1159/000507450. Epub ahead of print. PMID: 32209789.
26. Santos AR, Raimundo M, Alves D, Lopes M, Pestana S, Figueira J, Cunha-Vaz J, Silva R. Microperimetry and mfERG as functional measurements in diabetic macular oedema undergoing intravitreal ranibizumab treatment. Eye (Lond). 2021 May;35(5):1384-1392. doi: 10.1038/s41433-020-1054-2. Epub 2020 Jul 2. PMID: 32616867.
