# Supplementary material for: A New Approach to Staging Diabetic Eye Disease: Staging of Diabetic Retinal Neurodegeneration and Diabetic Macular Edema
Source: Ophthalmol Sci. 2023 Oct 31;4(3):100420. doi: 10.1016/j.xops.2023.100420 (PMC10818256; doi:10.1016/j.xops.2023.100420)
Supplement: Table S5 [file mmc5.pdf]

**Parameter**	
Parameter name	Vascular changes in the macula of patients with diabetic macular edema
Search terms	“optical coherence tomography” and “angiography” and "diabetic macular edema”
Search date	September 20, 2021
Search results	525
Pruning	Excluded case reports and case series, articles not in english, reviewed abstracts and excluded studies that did not answer questions in the grid
How is this parameter assessed?	OCT-A: Regular and Swept-source
Has analytical validation been accomplished?<br>If yes, give specifics of evaluation of parameter precision, accuracy, limit of detection, limit of quantitation, specificity, linearity and range, ruggedness and robustness.	Overall reproducible metrics, but macular edema affects segmentation of the algorithm and makes it less reproducible.<br><br>Majority of studies have looked at DR as opposed to DME.<br><br>Repeatability metrics in patients with DM and any levels of DR **but no pronounced edema** using the **AngioVue OCTA**: ICC > 0.90 for both intrasession and between-visit comparisons for FAZ and vessel density (VD) at 3mm and parafoveal ring. Intrasession Coefficient of Variation (CoV), defined as the ratio of the standard deviation to the mean, was 7.8, 2.9.3.6 for FAZ, VD at 3mm and VD parafoveally and<br>Intersession CoV was 12.3, 2.95 and 4.03 for FAZ, VD at 3m and VD at parafoveal ring respectively. (1)<br><br>SD-OCTA and SS-OCTA data are not comparable. “Results obtained with SD-OCTA (AngioPlex) and SS-OCTA(PlexElite) for the same eyes and area showed a Pearson correlation of 0.7(2).<br><br>**SSOCT reliability metrics:** including 30 eyes with no DR, all levels of DR, excluded eyes with poor quality, motion artifacts, inaccurate segmentation: *13% of included eyes had DME and 14% of excluded eyes had DME*. (3)

	Intra-section		Inter-section	
	ICC (95% CI)	CR (95% CI)	ICC (95% CI)	CR (95% CI)
FAZ Area (mm^2)	0.976 (0.950–0.988)	0.040 (0.036–0.043)	0.967 (0.932–0.984)	0.050 (0.045–0.055)
FAZ Circularity	0.751 (0.540–0.873)	0.125 (0.114–0.138)	0.720 (0.490–0.856)	0.129 (0.117–0.141)
Total Vessel Density (%)	0.840 (0.691–0.920)	0.069 (0.063–0.076)	0.777 (0.583–0.887)	0.090 (0.081–0.098)
Parafoveal Vessel Density (%)	0.826 (0.667–0.913)	0.078 (0.071–0.085)	0.774 (0.577–0.885)	0.098 (0.088–0.107)
Fractal Dimension	0.989 (0.978–0.995)	0.010 (0.009–0.011)	0.987 (0.972–0.994)	0.010 (0.009–0.011)
Vessel Density Index (mm)	0.633 (0.358–0.807)	0.001 (0.0008–0.001)	0.624 (0.346–0.802)	0.001 (0.0008–0.001)

Table 2. Reliability estimates of quantitative optical coherence tomography-angiography (OCT-A) metrics measurements using an automated analysis program in a subset of 30 eyes from 30 diabetic participants. Two OCT-A scans were acquired during first visit for these participants for intra-session repeatability assessment. These 30 patients were also invited for another visit for OCT-A scan within 2 weeks for inter-session reproducibility assessment. CR: Coefficient of repeatability; FAZ: Foveal avascular zone; ICC: Intra-class correlation coefficient.

Measurements from **7 different devices for 18 healthy volunteers** were compared for VD, FD and FAZ for SCP or DCP(4): Different OCTA devices use different algorithms (e.g.Optovue uses SSADA, Spectralis uses the Probablistic algorithm, Angioplex and PlexElite use the OMAG algorithm, Nidek uses CODAA, Canon OCT HS-100 uses Full spectrum and Optopol uses SOA. They also use different boundaries with different offsets to identify the superficial and deep capillary plexi. Superficial VD values ranged from: 22.6-45.2
VD Deep: 38.9-48.4. Depending on the devices the differences were smaller between some devices than others. The authors presented 21 comparisons (i.e. of each device vs another) for SVD, DVD, FD, FAZ. When choosing devices need to determine how the outputs differ from each other, plus need to assess for the ability to change segmentation boundaries so that they are similar across devices.

Another study evaluated 4 devices. It used the older version of Heidelberg. Used Angiotool for vessel measurements and interestingly reported v similar density measurements but indicated that the correlation coeffcients were very weak (-0.16-0.35). “Overall vessel density among the evaluated modules using
Angiotool (Zeiss 48.7±4%, Optovue 47.9±3%, Topcon 48.3±2%, Heidelberg 46.5±4%, p = 0.2).”No graph presented, only coefficients given in supplemental table. Both superficial and deep capillary plexi were segmented per the methods section but only the superficial vessel density was assessed.(5).

Reliability of the metrics is affected by DME(6), this could be due to segmentation errors(7), the presence of fluid weakens the signal from the deeper layers, cystic spaces may compress the vessels and show falsely decreased perfusion as the flow decreases below the detection limits of OCTA(8). In addition, the extra-vascular fluid in cysts, may have suspended scattered particles in motion, and those may generate an OCT flow signal(9).

	Artifacts in OCTA with DME: in a study of 20 eyes with DME and 24 healthy eyes, it was observed that vessel density measurement errors occurred in all eyes with DME and in about a third of healthy eyes(10).<br><br>Studies have suggested that **3-D OCTA derived metrics** of perfusion (measurements done by the authors using Image J and semi-automated algorithms to visualize 3D OCTA data), have better repeatability metrics esp in the presence of DME, improvement is thought to be due avoiding the segmentation errors in 2D-OCT scans. In 20 eyes with DR and DME, ICC was 0.6-0.8 for 2D and 0.93-0.97 for 3D OCT metrics; Coefficient of Variation (CV) ranged from 2.2-4.2 for 2D and 1.9-2.0 for 3D(11)
What kind of variable is this (e.g. a binary event, time to event or quantitative/continuous outcome)?	Quantitative
Are there useful cut points or thresholds for outcome use?	No
**Scientific Understanding of Relationship to DRD**	
What is the biological, anatomic and/or functional rationale or plausibility for the association of this parameter with DRD?<br>(i.e., what is the degree to which diabetes triggers subsequent steps in a pathophysiologic pathway and the role of the parameter in that causal or outcome pathway)	Evaluates microvasculature, this is well established to be affected in DR.

What is the current understanding of the molecular mechanism(s) underlying the alterations in this parameter in association with DRD? (specify whether mechanisms are physiologic, pathologic or pharmacologic)	Ischemic and VEGF mediated changes in microvasculature
What is the outcome measure with which this parameter is associated?	**OCTA measures associated with progression of DR or development of DME:**<br>Authors used macular and peripapillary OCTA data from a subset of patients enrolled in the TIME2b clinical trial, and used one of 3 commercially available OCT devices: Zeiss Cirrus, Angiovue and Topcon SSOCT (patients with mod-severe DR were treated with AKB over 1 year). Images were graded by a reading center. Pre-treatment *larger FAZ area, presence of IRMA* was associated with **DME development**; IRMA and reduced peripapillary VD (in superior temporal and inferior temporal regions) **was associated with increased odds of DRSS progression** (12). *Limitation*: pooled data from 3 devices<br><br>Prospective study: using Topcon ssOCTA, 205 eyes(129 patients with DM), f/u 2 years. Automated measurements obtained using MATLAB regarding FAZ area, FAZ circularity, VD, fractal dimension(FD) of SCP and DCP obtained after quality check. **The FAZ area, VD, and FD of DCP predict DR progression, VD of SCP predicted DME development.** DR graded from FP using two field photos(13).<br><br>A prospective study of 16 eyes with persistent DME showed that superficial and deep capillary plexus densities decreased through 24 months despite continued aflibercept treatment(14).<br><br>**VA outcomes:**<br>In eyes with DME, parafoveal VD of the superficial layer was shown to be an independent predictor of visual acuity improvement after RBZ treatment (15).<br><br>Study of 24 eyes of 21 age/sex matched patients with resolved DME showed that Larger FAZ correlated with lower visual acuity(16).

	In a cases series of 77 eyes with DME, the vessel density of the DCP at the time of treatment initiation was significantly associated with the degree of recovery of the ellipsoid zone and improvement in visual acuity(17)<br><br>**Treatment response:**<br>Decreased DCP/SCP ratio noted in patients with poor treatment response: poor response was defined as needing > 5 Avastin treatments in a year after receiving an initial dex implant. Swept source OCT was used. Compared several metrics and found DCP/SCP ratio to be significant (18).<br>Suspended Scattered Particles in Motion observed by OCTA have been shown to be associated with treatment response in a study analyzing 45 patients with DME, 24 of whom were treated with anti-VEGF and 21 with a steroid(17).<br><br>This study showed that MAs as detected by ssOCTA decreased in response to antiVEGF treatment(19). "Foveal avascular zone enlargement, high number of microaneurysms (MAs), lower vessel density (VD) in deep capillary plexus and lower parafoveal VD in superficial capillary plexus were considered as OCT angiography biomarkers of poor responsiveness."<br><br>**DRIL:**<br>Study of 24 eyes of 21 age/sex matched patients with resolved DME showed larger FAZ in eyes with DRIL compared to without DRIL(16)
What is the link between the parameter and the accepted clinical outcome measure?	**See above:**<br>**<u>Information below is more relevant to DR rather than DME</u>**<br><br>**Fluorescein angiographic risk factors:**<br>Flourescein leakage, capillary loss, and capillary dilatation, and arteriolar staining are all associated with risk for progression to PDR at 1 year. Eyes with angiographic risk factor (compared to none at baseline) had a 1.7-to 3-fold increase in rates of PDR at 1 year, 1.4 to 2.5-fold increase at 3 years, and 1.2 to 1.8-fold increase at 5 years (20).

	**MAs:**<br>Number of MAs in stereoscopic 7-field photos positively associated with the progression of DR over 4 years(21).<br><br>Conflicting reports on the ability of OCTA to detect MAs compared to FA. Some report that the # of MAs detected on OCTA and FA is the same, others report that significantly lower MAs are detected on OCTA compared to FA, as low as 62%(22). Detection of MA on OCTA is influenced by its composition and reflectivity(8).<br><br>"The sensitivity of ssOCTA in detecting microaneurysms when compared with FA was 85% (95% CI, 53-97), while the specificity was 75% (95% CI, 21-98). The positive predictive value and the negative predictive value were 91% (95% CI, 59-99) and 60% (95% CI, 17-92), respectively": small sample size, only 17 DM eyes had FA and ssOCTA done and not always on the same day, time interval b/w the two images was up to 8 weeks(23).<br><br>**IRMA:**<br>IRMA is associated with worsening severity of DR.<br>This retrospective study showed that IRMA as evaluated on OCTA changed in appearance: regressed, stable, progressed or obliterated in anti-VEGF treated eyes. In control eyes (DR eyes with similar severity but no anti-VEGF treatment) none of the areas of IRMA regressed, they either remained stable, progressed or obliterated(24).<br><br>Higher detection of IRMA on OCTA compared to color fundus photos(25).<br><br>**NV:** Can detect NV and give more detailed info about the features of NV, authors proposed classification based on OCTA appearance(26).<br><br>**Non-perfusion via vessel density measurements,**<br>Decrease in vessel density associated with great DR severity. Observational cross-sectional study of 102 eyes with newly diagnosed NPDR. (27)<br><br>Retrospective cross-sectional study of 137 eyes (86 patients) with different stages of DR (no DR, NPDR, PDR) compared to

	44 eyes (26 healthy age matched controls). FAZ, VD, and percent area of non-perfusion (PAN) were significantly associated with DR severity. There was a difference between patients with diabetes without DR, and healthy controls in PAN measured in the SCP, full retina and choriocapillaris. Non-perfusion of the retinal capillary plexus was strongly correlated with DR severity.(28)<br><br>VD may be decreased in DM even before the development of DR.(22) However, there is a lot of variability in the measurements of patients with different DR severity and no DR. Authors reported significantly decreased parafoveal density in the SCP using ssOCTA, but review of the data and confidence intervals showed that the values from non-DR, mild DR and VTDR were overlapping: this was a study with one of the larger sample sizes: 919 patients (29). Others have suggested using spacing between vessels rather than density measurements as a better measure of DR severity(30).<br><br><br>**FAZ: (area, circularity(shape), radius)**<br>ICC between FAZ measurement on ssOCTA and FA high(0.983), mean difference -0.01mm^2.(3)<br>Generally enlarges in eyes with DR, loss of circularity in the superficial and deep plexi even in DM without clinical signs of DR(22)<br><br>Additional parameters: PP vessel density. PP RNFL thickness, GCC thickness, blood vessel tortuosity (BVT), blood vessel caliber (BVC), vessel perimeter index (VPI),), vessel complexity index (VCI), branchpoint analysis (BPA), differential artery–vein (A–V) analysis, flow analysis, Choriocapillaris metrics(31)<br><br>Compromised foveal microvascular integrity (i.e. lower vessel density within 300 microns around the fovea) was associated with sub-optimal clinical course when treating DME with anti-VEGF agents(32).
**Performance Expectations in DRD**	

What sensitivity to detect change does this parameter provide compared to the current standard (if available)?	Detection of capillary non-perfusion was higher in an observational study with ss-WF-OCTA as compared to UWF-FA in 10 eyes of 9 patients with severe NPDR or PDR(33).<br><br>“The **sensitivity of ssOCTA in detecting microaneuryms when compared with FA was 85% (95% CI, 53-97), while the specificity was 75%** (95% CI, 21-98). The positive predictive value and the negative predictive value were 91% (95% CI, 59-99) and 60% (95% CI, 17-92), respectively”: *small sample size*, only 17 DM eyes had FA and ssOCTA done and not always on the same day, *time interval b/w the two images was up to 8 weeks*. (23)<br><br>**NV:** Can detect NV and give more detailed info about the features of NV, authors proposed classification based on OCTA appearance(26).
Is there consistency of response across species?<br>If yes, please explain	Yes, similar findings across species
Is there consistency of response across mechanistically or mechanically distinct interventions?<br>If yes, please explain	
Is there a dose response to the magnitude of changes in this parameter and changes in the clinical outcome?<br>If yes, please give specifics of that relationship	Yes, quantitative OCTA metrics have been correlated with DR severity and development of DME.(8, 34)<br><br>See above for specifics regarding changes in clinical outcome
Is there a temporal relationship between changes in this parameter and the clinical outcome?	Yes – few prospective studies suggestive of OCTA features being predictive of incident DME: see above.

If yes, please give specifics of that relationship	
What is the specificity of changes in this parameter for DRD?	Enlarged FAZ is associated with shorter axial length and thinner central sub-field thickness. There is a wide variation in FAZ area (0.071 -0.527mm2) in healthy eyes with 20/20 VA(35).<br><br>Decreased SCP-VD was correlated with thinner macular GC-IPL(3).<br>Density metrics are associated with age(34) and gender and highly correlated with signal strength(8).
**Types of Data and Available for Evidential Evaluation**	
Are there preclinical studies that address the relationship of this parameter to outcomes in DRD?	Yes
If yes, please summarize the available evidence from *in silico* studies	
References for *in silico* studies	
If yes, please summarize the available evidence from *in vitro* studies	
References for *in vitro* studies	
If yes, please summarize the available evidence from *in vivo* studies	Detection of microvascular retinal changes in type I diabetic mice with optical coherence tomography angiography (Uehara)<br><br>OCT-A detects age dependent retinal vascular differences between type 1 diabetic mice (Ins2$^{Akita/+}$) and control mice.

	Vascular density was decreased in the diabetic mice compared to controls at both 2 and 6 months of age (n=4 in each group).(36) In the superficial, intermediate and deep layers, vascular volume density was lower in the 6month old diabetic mice (compared to WT and 2-month old diabetic mice). Aged diabetic mice showed inner plexiform layer loss. *Additional info about animal studies in the OCT-grid.*
References for *in vivo* studies	
Are there clinical studies that address the relationship of this parameter to outcomes in DRD?	yes
If yes, which of the following clinical study types have been performed: systematic review, prospective randomized controlled trial, retrospective randomized controlled trial, cohort study, case/control study?	Prospective cohort studies, retrospective, case/control studies
If yes, please summarize the available evidence from clinical studies	See details above
References for clinical studies	
Are there literature reviews that address the relationship of this parameter to outcomes in DRD?	No reviews addressing this to outcomes in DRD or DME

References for literature reviews	
Please give the Level of Evidence available from these combined studies (use Tables 1 and 2 below to determine Level of Evidence. For this purpose, please substitute “DRD parameter” for “tumor marker” or “marker” in Table 1)	Level 2B<br>(Two prospective studies showed associated between OCTA metrics and risk of DME and DRSS progression but identified different OCTA metrics; another caveat is that the one of the studies used data from the TIME2b trial, the original trial was not powered to study the DRD marker i.e. OCTA metrics).
**Statistical Considerations**	
What is the specific relationship of the parameter to clinical outcomes?<br>Please specify effect sizes and measures of variability	Vessel density metrics on OCTA have been linked to more severe DR, associated with DR progression and development of DME.
What is the usefulness of the parameter or its thresholds for clinical or research decision making?	Lack of consensus regarding which variable to use from the many parameters provided by OCTA.<br><br>There are some normative data available in the literature but these will likely vary between devices and across ethnicities. 135 eyes from 70 healthy Caucasian adults: *“at the level of the SCP, the mean VD and mean FAZ area were respectively 52.58 ± 3.22% and 0.28 ± 0.1 mm^2 in the whole en face image. At the level of the DCP, mean VD and mean FAZ area were respectively 57.87 ± 2.82% and 0.37 ± 0.12 mm^2 in the whole en face image”(37)*<br><br>There is a wide variation in FAZ area (0.071 -0.527mm^2) for superficial plexus and (0.160 mm^2–0.795 mm^2) for deep plexus among healthy eyes with 20/20 VA(35). This study did not find a significant correlation with age or gender. Area was inversely correlated with central macular thickness and central macular volume. Retinal vascular density measurements are significantly associated with FAZ(38). Another study among Chinese adults

	found that gender, axial length, retinal thickness including RNFL and GCL thickness affected FAZ area(39).
Are there covariates that should be adjusted for when considering this parameter?	Yes, age, gender, ethnicity, scan quality, axial length, retinal thickness impact vessel density measurements. Density metrics are associated with age(34) and gender and highly correlated with signal strength(8).
Are there any additional statistical considerations for the use of this parameter?	OCTA gives many parameters, diversity in scanning patterns (3mm vs 6mm, macula vs peripapillary), need to evaluate which parameters and scanning modalities are the most reliable to assess risk of DME development or response to treatment and to adjust for the many parameters. Also important to control for multiple statistical comparisons when presenting the results.
**Gap Analysis**	
What are the gaps in the literature to prove or disprove the utility of this parameter?	Lack of consistency of parameters across different devices, different OCTA instruments display different tissue reflectivity that influences flow detection. DME characteristics e.g. cysts and non-perfused areas show up differently in devices that use different algorithms e.g. split-spectrum amplitude-decorrelation algorithm (SSADA) used by Angiovue OCTA vs optical microangiography (OMAG) algorithm used by the Zeiss by Angioplex and Plex Elite Zeiss models. (40).<br><br>Lack of a head-to-head comparison between OCT-A and FP/FA to see if it can reliably identify the FA factors that have been shown to be associated with risk of progression in the ETDRS.
In your opinion, what clinical research study/studies could address these gaps?	Incorporate OCTA into prospective studies and compare head-to-head with existing modalities i.e. color fundus photos and FA and evaluate if treatment plan/outcome changes.<br><br>More studies on how to pool OCT-A measurements from different devices. This may require using the same segmentation boundaries, offsets, same area of measurement (e.g 3x3mm) and same number of B-scans within that area. We may need to develop different thresholds and/or conversion equations similar to central foveal thickness for OCT.

	Prospective hypothesis driven studies to evaluate which OCTA output are predictive of meaningful outcome (e.g. DME development)<br>*prior studies described above have suggested that FAZ area (although irregularity of FAZ boundary may be more important), presence of IRMA, VD when controlled for confounders are associated with DME development*.
Are there currently available datasets that could be used for these validation efforts?	Some of the data collected as part of clinical trials could probably be used and evaluated in a masked fashion by a reading center.
**Miscellaneous Questions**	
Is this parameter currently employed in clinical use?	yes
Is assessment instrumentation needed to measure this parameter currently: available commercially, available but not FDA approved, not readily available, or not available?	yes
What is the ease of implementation in the following environments: high resource academic center, high resource community practice, low resource/underserved environment?	Easy, in high resource academic centers, smaller practices may not own one
What sites are appropriate for this assessment?<br>Indicate all relevant site types: retina clinic, general ophthalmology	retina clinic, general ophthalmology clinic

clinic, optometry clinic, endocrinology clinic, general medical clinic, patient home.	
Is there any technology or advance either currently available, in development, or not yet developed that would make this parameter no longer important or relevant?<br>If yes, please specify what technology or advance	Not known
What unmet need in the staging of DRD does this parameter address?	Early and non-invasive detection of microvascular abnormalities
**Summary**	
Based on the above data, please provide an integrated evaluation regarding the overall importance of this parameter to the field currently. If not currently relevant, please summarize the potential for future relevance, necessary steps for validation and a reasonable time frame for this to occur.	It definitely has potential as a non-invasive device that gives detailed information about the retinal microvasculature as well as structural information regarding the different retinal layers. However, scanning protocols and parameters to be used need to be standardized. Analytical methods should also account for the multiple measurement outputs from one eye.

1. Czakó C, Sándor G, Ecsedy M, Récsán Z, Horváth H, Szepessy Z, et al. Intrasession and between-visit variability of retinal vessel density values measured with OCT angiography in diabetic patients. Scientific Reports. 2018;8(1):1-8.
2. Santos T, Warren LH, Santos AR, Marques IP, Kubach S, Mendes LG, et al. Swept-source OCTA quantification of capillary closure predicts ETDRS severity staging of NPDR. British Journal of Ophthalmology. 2020.
3. Tang FY, Ng DS, Lam A, Luk F, Wong R, Chan C, et al. Determinants of quantitative optical coherence tomography angiography metrics in patients with diabetes. Scientific reports. 2017;7(1):1-10.
4. Corvi F, Pellegrini M, Erba S, Cozzi M, Staurenghi G, Giani A**.** Reproducibility of vessel density, fractal dimension, and foveal avascular zone using 7 different optical coherence tomography angiography devices. American journal of ophthalmology. 2018;186:25-31.
5. Munk MR, Giannakaki-Zimmermann H, Berger L, Huf W, Ebneter A, Wolf S, et al. OCT-angiography: A qualitative and quantitative comparison of 4 OCT-A devices. PloS one. 2017;12(5):e0177059.
6. Borrelli E, Sacconi R, Parravano M, Costanzo E, Querques L, Battista M, et al. OPTICAL COHERENCE TOMOGRAPHY ANGIOGRAPHY ASSESSMENT OF THE DIABETIC MACULA: A Comparison Study Among Different Algorithms. Retina. 2021;41(9):1799-808.
7. Lee J, Moon BG, Cho AR, Yoon YH**.** Optical Coherence Tomography Angiography of DME and Its Association with Anti-VEGF Treatment Response. Ophthalmology. 2016;123(11):2368-75.
8. Sun Z, Yang D, Tang Z, Ng DS, Cheung CY**.** Optical coherence tomography angiography in diabetic retinopathy: an updated review. Eye (Lond). 2021;35(1):149-61.
9. Kashani AH, Green KM, Kwon J, Chu Z, Zhang Q, Wang RK, et al. Suspended scattering particles in motion: a novel feature of OCT angiography in exudative maculopathies. Ophthalmology retina. 2018;2(7):694-702.
10. Ghasemi Falavarjani K, Habibi A, Anvari P, Ghasemizadeh S, Ashraf Khorasani M, Shenazandi H, et al. Effect of segmentation error correction on optical coherence tomography angiography measurements in healthy subjects and diabetic macular oedema. Br J Ophthalmol. 2020;104(2):162-6.
11. Borrelli E, Parravano M, Costanzo E, Sacconi R, Querques L, Pennisi F, et al. Using three-dimensional OCTA metrics improves repeatability on quantification of ischemia in eyes with diabetic macular edema. Retina. 2020;Publish Ahead of Print.
12. Greig EC, Brigell M, Cao F, Levine ES, Peters K, Moult EM, et al. Macular and Peripapillary Optical Coherence Tomography Angiography Metrics Predict Progression in Diabetic Retinopathy: A Sub-analysis of TIME-2b Study Data. American Journal of Ophthalmology. 2020;219:66-76.
13. Sun Z, Tang F, Wong R, Lok J, Szeto SKH, Chan JCK, et al. OCT Angiography Metrics Predict Progression of Diabetic Retinopathy and Development of Diabetic Macular Edema: A Prospective Study. Ophthalmology. 2019;126(12):1675-84.
14. Statler B, Conti TF, Conti FF, Silva FQ, Rachitskaya A, Yuan A, et al. Twenty-Four-Month OCTA Assessment in Diabetic Patients Undergoing Fixed-Interval Intravitreal Aflibercept Therapy. Ophthalmic Surg Lasers Imaging Retina. 2020;51(8):448-55.
15. Hsieh YT, Alam MN, Le D, Hsiao CC, Yang CH, Chao DL, et al. OCT Angiography Biomarkers for Predicting Visual Outcomes after Ranibizumab Treatment for Diabetic Macular Edema. Ophthalmol Retina. 2019;3(10):826-34.

16. Moein H-R, Novais EA, Rebhun CB, Cole ED, Louzada RN, Witkin AJ, et al. OPTICAL COHERENCE TOMOGRAPHY ANGIOGRAPHY TO DETECT MACULAR CAPILLARY ISCHEMIA IN PATIENTS WITH INNER RETINAL CHANGES AFTER RESOLVED DIABETIC MACULAR EDEMA. RETINA. 2018;38(12):2277-84.
17. Moon BG, Um T, Lee J, Yoon YH**.** Correlation between Deep Capillary Plexus Perfusion and Long-Term Photoreceptor Recovery after Diabetic Macular Edema Treatment. Ophthalmol Retina. 2018;2(3):235-43.
18. Park YG, Park YH**.** Quantitative Analysis of Retinal Microvascular Perfusion and Novel Biomarkers of the Treatment Response in Diabetic Macular Edema. J Diabetes Res. 2020;2020:2132037.
19. Pongsachareonnont P, Charoenphol P, Hurst C, Somkijrungroj T**.** The Effect of Anti-Vascular Endothelial Growth Factor on Retinal Microvascular Changes in Diabetic Macular Edema Using Swept-Source Optical Coherence Tomography Angiography. Clin Ophthalmol. 2020;14:3871-80.
20. Fluorescein angiographic risk factors for progression of diabetic retinopathy. ETDRS report number 13. Early Treatment Diabetic Retinopathy Study Research Group. Ophthalmology. 1991;98(5 Suppl):834-40.
21. Klein R, Meuer SM, Moss SE, Klein BE**.** The relationship of retinal microaneurysm counts to the 4-year progression of diabetic retinopathy. Archives of Ophthalmology. 1989;107(12):1780-5.
22. Cicinelli MV, Cavalleri M, Brambati M, Lattanzio R, Bandello F**.** New imaging systems in diabetic retinopathy. Acta Diabetol. 2019;56(9):981-94.
23. Salz DA, Talisa E, Adhi M, Moult E, Choi W, Baumal CR, et al. Select features of diabetic retinopathy on swept-source optical coherence tomographic angiography compared with fluorescein angiography and normal eyes. JAMA ophthalmology. 2016;134(6):644-50.
24. Sorour OA, Mehta N, Baumal CR, Ishibazawa A, Liu K, Konstantinou EK, et al. Morphological changes in intraretinal microvascular abnormalities after anti-VEGF therapy visualized on optical coherence tomography angiography. Eye Vis (Lond). 2020;7:29.
25. Schaal KB, Munk MR, Wyssmueller I, Berger LE, Zinkernagel MS, Wolf S**.** Vascular abnormalities in diabetic retinopathy assessed with swept-source optical coherence tomography angiography widefield imaging. Retina. 2019;39(1):79-87.
26. Pan J, Chen D, Yang X, Zou R, Zhao K, Cheng D, et al. Characteristics of neovascularization in early stages of proliferative diabetic retinopathy by optical coherence tomography angiography. American journal of ophthalmology. 2018;192:146-56.
27. Sambhav K, Abu-Amero KK, Chalam KV**.** Deep Capillary Macular Perfusion Indices Obtained with OCT Angiography Correlate with Degree of Nonproliferative Diabetic Retinopathy. Eur J Ophthalmol. 2017;27(6):716-29.
28. Nesper PL, Roberts PK, Onishi AC, Chai H, Liu L, Jampol LM, et al. Quantifying Microvascular Abnormalities With Increasing Severity of Diabetic Retinopathy Using Optical Coherence Tomography Angiography. Invest Ophthalmol Vis Sci. 2017;58(6):Bio307-bio15.
29. Xie N, Tan Y, Liu S, Xie Y, Shuai S, Wang W, et al. Macular vessel density in diabetes and diabetic retinopathy with swept-source optical coherence tomography angiography. Graefes Arch Clin Exp Ophthalmol. 2020;258(12):2671-9.
30. Bhanushali D, Anegondi N, Gadde SG, Srinivasan P, Chidambara L, Yadav NK, et al. Linking retinal microvasculature features with severity of diabetic retinopathy using optical coherence tomography angiography. Investigative ophthalmology & visual science. 2016;57(9):OCT519-OCT25.

31. Yao X, Alam MN, Le D, Toslak D. Quantitative optical coherence tomography angiography: A review. Exp Biol Med (Maywood). 2020;245(4):301-12.
32. Huang WH, Lai CC, Chuang LH, Huang JC, Wu CH, Lin YT, et al. Foveal Microvascular Integrity Association With Anti-VEGF Treatment Response for Diabetic Macular Edema. Invest Ophthalmol Vis Sci. 2021;62(9):41.
33. Couturier A, Rey PA, Erginay A, Lavia C, Bonnin S, Dupas B, et al. Widefield OCT-Angiography and Fluorescein Angiography Assessments of Nonperfusion in Diabetic Retinopathy and Edema Treated with Anti-Vascular Endothelial Growth Factor. Ophthalmology. 2019;126(12):1685-94.
34. Durbin MK, An L, Shemonski ND, Soares M, Santos T, Lopes M, et al. Quantification of Retinal Microvascular Density in Optical Coherence Tomographic Angiography Images in Diabetic Retinopathy. JAMA Ophthalmology. 2017;135(4):370-6.
35. Samara WA, Say EA, Khoo CT, Higgins TP, Magrath G, Ferenczy S, et al. Correlation of foveal avascular zone size with foveal morphology in normal eyes using optical coherence tomography angiography. Retina. 2015;35(11):2188-95.
36. Uehara H, Lesuma T, Stocking P, Jensen N, Kumar SR, Zhang MA, et al. Detection of microvascular retinal changes in type I diabetic mice with optical coherence tomography angiography. Exp Eye Res. 2019;178:91-8.
37. Coscas F, Sellam A, Glacet-Bernard A, Jung C, Goudot M, Miere A, et al. Normative data for vascular density in superficial and deep capillary plexuses of healthy adults assessed by optical coherence tomography angiography. Investigative ophthalmology & visual science. 2016;57(9):OCT211-OCT23.
38. Fujiwara A, Morizane Y, Hosokawa M, Kimura S, Shiode Y, Hirano M, et al. Factors affecting foveal avascular zone in healthy eyes: An examination using swept-source optical coherence tomography angiography. Plos one. 2017;12(11):e0188572.
39. Zhou Y, Zhou M, Gao M, Liu H, Sun X. Factors Affecting the Foveal Avascular Zone Area in Healthy Eyes among Young Chinese Adults. BioMed research international. 2020;2020.
40. Parravano M, Costanzo E, Borrelli E, Sacconi R, Virgili G, Sadda SR, et al. Appearance of cysts and capillary non perfusion areas in diabetic macular edema using two different OCTA devices. Sci Rep. 2020;10(1):800.
