# Supplementary material for: A New Approach to Staging Diabetic Eye Disease: Staging of Diabetic Retinal Neurodegeneration and Diabetic Macular Edema
Source: Ophthalmol Sci. 2023 Oct 31;4(3):100420. doi: 10.1016/j.xops.2023.100420 (PMC10818256; doi:10.1016/j.xops.2023.100420)
Supplement: Table S6 [file mmc6.pdf]

**Parameter**				
Parameter name	Macular retinal nerve fiber layer thickness (mRNFL)	mGCL	mGC-IPL	pRNFL
Search Terms	"retinal neurodegeneration" and "diabetes";<br>"retinal nerve fiber layer" and "optical coherence tomography" and "diabetes";<br>"ganglion cell inner plexiform layer" and "optical coherence tomography" and "diabetes";<br>"ganglion cell layer" and "optical coherence tomography" and "factors";<br>"neurodegeneration" and "diabetes" and "animal" and "optical coherence tomography";<br>retina neurodegeneration diabetes mice optical coherence tomography;<br>"swept source optical coherence tomography" and "diabetes"<br>(Corneal neurodegeneration) and (microvascular);<br>(diabetic peripheral neuropathy) and (neurodegeneration) and (optical coherence tomography);<br>((diabetes)) AND (optical coherence tomography)) AND (animal model));<br>((retinal nerve fiber layer) AND (diabetes)) AND (optical coherence tomography) AND (animal model));<br>(diabetes) AND (neurodegeneration eye) AND (mouse) AND (optical coherence tomography);<br>Original Search Date: 11/16/2020; updated 9/9/2021 in Pubmed			
Search results	754			
Pruning	Not diabetes, not reporting structural OCT changes in retina, not case-series, not in english<br>reviewed abstracts and excluded studies that were not relevant to the questions in the grid<br>Preference given to prospective longitudinal studies, large cross-sectional studies and meta-analyses			
How is this parameter assessed?	SD-OCT			
Has analytical validation been accomplished?<br>If yes, give specifics of evaluation of parameter precision, accuracy, limit of detection, limit of quantitation, specificity, linearity and range, ruggedness and robustness.	RTVue 100 (normal eyes): Intraobserver, repeatability: 0.13 micron (+/- 0.53); inter-observer: 0.27(+/- 0.59); Coefficients of repeatability 1.84%; Coefficient of reproducibility: 2.72% [1]	For normal eyes, Zeiss Cirrus: <u>For each segmented retinal layer</u> (therefore applies to the other columns as well) thickness, the average difference between repeated measurements with registration was: 0.46 ± 0.25 µm (1.70 ± 0.72 %)[2]	In normal eyes for average GC+IPL thickness: ICC was 99.6-99.7, coefficient of variation 0.63- 0.67%, and test-retest variability (TRTV) ranged from 1-1.1 µm for intraobserver and interobserver reproducibility[3]<br><br>RTVue 100 (normal eyes): Intraobserver,	For average RNFL thickness: COR were 5.30 and 6.05 µm for the first and the second operator, interoperator ICCs of 0.95 and 0.96 for the first and the second session, and intersession CCCs of 0.96 and 0.97 for the first and second operator[4]”

			Repeatability: -0.27 micron (+/- 0.82); inter-observer: 0.14(+/- 1.00); Coefficients of repeatability 1.74%; Coefficient of reproducibility: 2.15% [1]	
What kind of variable is this (e.g. a binary event, time to event or quantitative/continuous outcome)?	Quantitative/Continuous			
Are there useful cut points or thresholds for outcome use?	No there is no cut point available. Important to control for factors that can impact RNFL and GCL. Change over time may be more useful.		The propriety software uses a normative database to identify thinning. Factor affecting thickness include age, axial length, sex and RNFL thickness.[5]	Glaucoma related cut points
**Scientific Understanding of Relationship to DRD**				
What is the biological, anatomic and/or functional rationale or plausibility for the association of this parameter with DRD? (i.e., what is the degree to which diabetes triggers subsequent steps in a pathophysiologic pathway and the role of the parameter in that causal or outcome pathway)	Animal studies and studies from donor eyes show gliosis, apoptosis of neuronal cell bodies (details of studies in sections below). Studies from streptozocin rat models suggest that diabetes leads to retinal dysfunction with ERG changes. This is followed by abnormalities in glutamate metabolism. This may be followed by vascular leakage and increased vegf expression and increased muller cell density. This is followed by neuronal apoptosis and retinal thinning. GFAP increases in Muller cells and this is followed by capillary changes and appearance of MAs [6]. Recent studies using mouse models that are more representative of Type 2 DM such as BTBR ob/ob mice, show thinning of inner retina on OCT and early functional deficits. At 20 weeks, thickness of the entire retina including inner and outer segments, ONL, INL and inner retina were reduced in the diabetic mice. On histological exam, counts of the number of nuclei in the two nuclear layers in the retina, suggests that the greatest reduction was present in cells of the inner retina. [7]			
What is the current understanding of the molecular mechanism(s) underlying the alterations in this parameter in association with DRD? (specify whether mechanisms are physiologic, pathologic or pharmacologic)	**Glutamate excitotoxicity:** Animal-based studies have shown that elevated glutamate concentrations were toxic to retinal ganglion cells and that these effects could be blocked by memantine.[8] **Mechanism:** Hyperglycemia>> decreased activity of glutamine synthetase & glutamate uptake decreased by Muller cells>>excessive glutamate accumulation>> neuronal cell loss [9-11]			

	**Role of PEDF**: Neuroprotective and anti-angiogenic effects of PEDF[12,13], PEDF may work by increasing the expression of glutamine synthetase. [14]. Topically administered PEDF eye drops "prevented diabetes-induced microglia activation by ~60%, retinal ganglion cell (RGC) death by ~22% and inner plexiform layer thinning by ~13%"[15]<br><br>"In diabetes, levels of all three growth factors (PEDF[13], BDNF, [16]and NGF[17] are reduced in association with retinal neurodegeneration".<br><br>PEDF administration in diabetic animals was shown to improve ERG responses as well as vascular hyperpermeability. [18]<br>**Somatostatin (SST):** is produced in the retina, in amacrine cells[19] and also in the RPE[20]. SST is thought to have neuroprotective effects. **<u>Mechanism:</u>** Neurodegenerative changes rather than ischemia may lead to decreased SST in diabetes<br>**<u>Evidence:</u>** Ischemia absent but SST levels of vit found to be low in patients with DME[21]<br>Study compared SST mRNA and SST immunoreactivity in 10 human retinas from diabetics (without DR on fundoscopy 2 years before death) and 10 non-diabetics showed a reduction of SST mRNA and SST-23 immunofluorescence in diabetic retina and associated glial activation and neural death. Total thickness of the neuroretina was lower in diabetics compared to nondiabetic retinas. [22]<br>Lower SST levels in vitreous of patients with PDR[23] and DME compared to plasma[21]<br>Topical SST eye drops can prevent retinal neurodysfunction and neurodegeneration in db/db mice. [24]<br><br>Wang *et al* showed that the lack of Sigma1R in Ins2Akita mice worsened neurodegeneration, as evaluated on OCT examination.[25]		
What is the outcome measure with which this parameter is associated?	MICROPERIMETRY[26], visual function as assessed on Rarebit visual field test[27], possible DR development[28]		Visual field changes (extensive literature from glaucoma)
What is the link between the parameter and the accepted clinical outcome measure	NO Definitive ASSOCIATION WITH DRSS<br><br>*<u>Meta-analysis[29]:</u>*<br>*<u>No DR vs normal controls:</u>*<br>No DR eyes had **significant thinning in mean m-RNFL**	*<u>Meta-analysis[29]:</u>*<br>*<u>No DR vs normal controls:</u>*<br>No DR eyes showed **significant thinner mean m-GCC than control group among** 11 cohorts (SMD = −0.28; 95%CI = [−0.48, −0.09]; p = 0.009).<br><br><u>No DR vs NPDR:</u><br>No significant differences among five cohorts<br><br>*<u>Meta-analysis[29]:</u>*<br>*<u>No DR vs normal controls:</u>*	*<u>Meta-analysis[29]:</u>*<br>*<u>No DR vs normal controls:</u>*<br>No significant differences were observed between No DR<br>eyes and control eyes in the mean p-RNFL thickness among 26 cohorts<br>as well as all sectors (all p > 0.05)

	**compared with control** among nine cohorts (SMD=−0.26; 95%CI=[−0.51, −0.01]; p=0.046)<br><br>*No DR vs NPDR:*<br>No differences noted<br><br>8-year follow-up study of Type1 DM patients: v hard to follow paper but compares GCL/INL/ RNFL in DM and controls over time, showing decrease in thickness over time in both groups. INL loss was only noted in the DM group. [30]	**m-GCIPL thickness in No DR eyes was significantly less compared with control eyes** among sixteen cohorts in a recent meta-analysis (SMD = −0.26; 95%CI = [−0.42, −0.11]; p = 0.003).<br><br>No DR vs NPDR:<br>No differences between NPDR or DR groups in mean or sectoral mGC-IPL thickness[29]		No DR vs NPDR:<br>NPDR eyes had significant thinning in mean p-RNFL compared with No DR eyes among 16 cohorts (SMD = −0.27; 95%CI = [−0.51, −0.03]; p = 0.03). In the sectoral analysis, similar finding was observed in the inferior quadrant among nine cohorts [(SMD = −0.35; 95%CI = [−0.64, −0.06]; p = 0.025), but not in other quadrants).
**Performance Expectations in DRD**				
What sensitivity to detect change does this parameter provide compared to the current standard (if available)?	There is no current standard available to detect neurodegenerative changes in DM.<br><br>In 45 people with DM and no to minimal DR there was decrease in RNFL (0.25 microm/y) [31]		87 eyes with type 2 DM were followed for 4 years: Average progressive mGCIPL thickness loss in the T2DM group was 0.38 microm/year compared to 0.18 microm/year in healthy controls.[32] Another series of 40 eyes showed a progressive loss of 0.45 microns per year. [33]<br><br>In 45 people with DM and no to minimal DR	164 eyes of 63 healthy individuals and 101 patients with Type 2 DM (49 with no DR and 52 with mild or moderate NPDR) were followed prospectively for 3 years and ppRNFL was evaluated yearly. “the estimated mean pRNFL loss was −0.92 µm/y in the non-DR group (P < .001) and −1.16 µm/y in the NPDR group (P < .001), which was 2.9-fold (95%CI, 1.1-14.8; P =

			there was decrease in GC-IPL (0.29 microm/yr) over 4 years [31]<br><br>In 117 eyes of patients with DM and no, mild or moderate DR: GCL loss was -0.539 +/- 0.15 microns/year and IPL loss of 0.361+/- 0.127 micros per year[34].<br><br>Lim et al compared the rate of GCL+IPL reduction between healthy control, patients with diabetes without DR and patients with mild-moderate NPDR based on annual OCT measurements over 3 years. They observed GCL+IPL thinning of −0.627 μm/year and −0.987 μm/year in the no-DR and NPDR group, which was 2.26-fold and 3.56-fold faster than in the control group[35].<br><br>Follow-up study of 125 patients with Type 2 DM showed that independent of development of DR, there was thinning in GCL and IPL over 1 year of follow-up[36]	.003) and 3.3-fold (95%CI, 1.4-18.0; P < .001) greater, respectively, than that of the control group (−0.35 μm/y; P = .01)”[37]

Is there consistency of response across species?<br>If yes, please explain	YES: animal models, donor eyes show evidence of neuronal degeneration. Individual OCT-based clinical studies show mixed results but meta-analyses show a significant thinning in mRNFL, mGCL and mGC-IPL of patients with DM (and no DR) compared to control eyes. pRNFL was significantly thinner in the NPDR vs no DR groups. [29]	
Is there consistency of response across mechanistically or mechanically distinct interventions?<br>If yes, please explain	No interventions have been definitively shown to be effective at present in preventing retinal neuronal degeneration in humans. EUROCONDOR results suggest that topical brimonidine and somatostatin can prevent progression of neurodysfunction (not structural changes) among a sub-group of patients who have neurodysfunction.[38]	
Is there a dose response to the magnitude of changes in this parameter and changes in the clinical outcome?<br>If yes, please give specifics of that relationship	None known	
Is there a temporal relationship between changes in this parameter and the clinical outcome?<br>If yes, please give specifics of that relationship	Yes, clinical and animal studies have identified structural OCT changes to occur prior to DR changes	
What is the specificity of changes in this parameter for DRD?	NOT SPECIFIC: Changes are associated with glaucoma, aging, sex, axial length, glaucoma , education score, cognitive impairment[39,40]<br>Changes seen in glaucoma, optic nerve disorders<br>GC-IPL Thickness is associated with RNFL thickness, age, axial length, sex [41]	Glaucoma, optic nerve diseases. **A study from 16, 084 European adults showed the following factors to be associated with reduced pRNFL thickness** as measured on SD-OCT: older age, higher intraocular pressure, visual impairment, history of systemic hypertension, and stroke. Factors associated with higher pRNFL thickness included: more hyperopic spherical equivalent, and smoking. [42]

**Types of Data and Available for Evidential Evaluation**				
Are there preclinical studies that address the relationship of this parameter to outcomes in DRD?	Yes, primarily in vivo studies			
If yes, please summarize the available evidence from *in silico* studies				
References for *in silico* studies				
If yes, please summarize the available evidence from *in vitro* studies				
References for *in vitro* studies				
If yes, please summarize the available evidence from *in vivo* studies	<u>**Different animal models for DM have been studied and shown neurodegenerative changes:**</u><br>Streptozocin-induced diabetic rats and spontaneously diabetic genetic model Ins2^{Akita} models are more representative of insulin-dependent diabetes.<br><br>**Pre-diabetic rat model (Wistar rats that drank high sucrose):** Authors observed decreased thickness of inner retinal layers in the pre-diabetic vs age-matched controls, while no changes were detected in permeability of blood-retinal barrier or the tight-junction protein content.[43]<br><br>**Otsuka Long-Evans Tokushima Fatty (OLETF) rats** have metabolic changes that are more similar to the course of human type 2 DM.<br>Earlier animal studies showed histological data, more recent studies also have OCT level data<br><br>“Total percentage of apoptotic cells in ONL, INL and GCL were significantly higher in diabetic mice compared with retinas from age-matched nondiabetic controls and diabetic mice treated with GLP-1R agonist had lower rate of apoptosis compared to mice treated with a vehicle. There is no difference in apoptotic cells percentage between mice treated with liraglutide (GLP-1R agonist) and those with restrictive diet”[44]<br><br>“significant, progressive inner retinal thinning in streptozotocin-induced “type 1” and B6.BKS(D)-Leprdb/J “type 2” diabetic mouse models on OCT; immunohistochemistry in type 1 mice showed GC loss but no difference in pericyte density or acellular capillaries.”[31]<br><br>“reduced thickness of IPL and INL; OPL and ONL did not change compared to controls; reduced ganglion cell body density in diabetic rats”[45]			

	**Ins2^{Akita} mouse: "**Compared with sibling control mice, the Ins2^{Akita} mice had increased retinal vascular permeability after 12 weeks of hyperglycemia (*P* < 0.005), a modest increase in acellular capillaries after 36 weeks of hyperglycemia (*P* < 0.0008), and alterations in the morphology of astrocytes and microglia, but no changes in expression of Müller cell glial fibrillary acidic protein. Increased apoptosis was identified by immunoreactivity for active caspase-3 after 4 weeks of hyperglycemia (*P* < 0.01). After 22 weeks of hyperglycemia, there was a 16.7% central and 27% peripheral reduction in the thickness of the inner plexiform layer, a 15.6% peripheral reduction in the thickness of the inner nuclear layer (*P* < 0.001), and a 23.4% reduction in the number of cell bodies in the retinal ganglion cell layer (*P* < 0.005)."[46]<br><br>The authors reported that the thickness of retinal nerve fiber layer as measured by OCT was decreased in Ins2Akita mice at 9 and 46 weeks of age, but no differences in the retinal vasculature were observed by FA. [47]<br><br>**C57Bl/6 mice made diabetic with streptozotocin: "**retinal cross sections of mice that had been diabetic 14 weeks showed ~20% to 25% fewer cells in the ganglion cell layer compared with age-matched control mice."[48]<br><br>**STZ mice:** The temporal changes associated with diabetes involving early loss of contrast sensitivity, ERG amplitudes, RNFL and GCC thinning on SD-OCT with immunohistochemical analyses linking these findings to retinal ganglion cell loss have been well characterized in this animal model.[49]<br><br>**Otsuka Long-Evans Tokushima Fatty rats (OLETF)**. Compared SD-OCT measurements of pRNFL in Diabetic OLETF (n=28 eyes) vs age-matched non-diabetic Long-Evans Tokushima Otsuka rats (LETO (n=38 eyes). The retinas did not have any obvious retinal disease in either rat prior to SD-OCT. at 36 weeks, the eyes were enucleated and histological exam was done evaluating the # of ganglion cells. "**RNFL thickness was significantly thinner in OLETF (28 weeks, 21.52 ± 1.91 µm and 36 weeks 21.08 ± 1.94 µm) than that in LETO (28 weeks, 24.75 ± 2.10 µm, *P* = 0.042 and 36 weeks, 24.37 ± 2.02 µm, *P* = 0.01**)". Interestingly the drop after 20 weeks in the diabetic mice was sudden. There was **no significant decrease in IPL thickness compared to non-diabetic mice. Different markers of apoptosis including caspase-3 and TUNEL showed increased apoptosis in the RNFL of diabetic vs non-diabetic mice.** [50]<br><br>**Ob/ob diabetic mice** showed significant thinning of the inner retinal layers on SD-OCT exam compared to controls. Histological studies also showed increased density of apoptotic cells in the GCL. Axon density was also reduced[7].<br><br>OCT examination of diabetic OM mice vs wild-type mice showed that there was no statistically significant difference between the two groups in RNFL thickness but the mean thickness of the NF-IPL layer was significantly lower in diabetic OB mice compared to control mice, capillary densities of the superficial and deep capillaries were unchanged[51].<br><br>**Meriones Shaw rodents:** animal model of Type 2 DM. Diabetes Animals were divided into 4 groups, two groups fed a high fat diet, two other served as age-matched controls. Retinal function was assessed using ffERG, retinal thickness

with OCT and retinal vessels with fundus and FA. Immunohistochemistry was performed to examine glutamate metabolism and synaptic transmission. Diabetic animals has delayed scotopic and photopic responses and decreases in scotopic a and b-wave amplitudes. Total Retinal thickness was significantly reduced, individual layer thickness was not assessed. No obvious vessel abnormality noted, except for some exudates visible in the periphery at 7 months. Immunohistochemistry showed a decreases in the expression of glutamate synthetase, vesicular glutamate transported and synaptophysin proteins.[52]

Interventions can decrease neurodegeneration in animal models of DM: Spermine oxidase; [53] Liraglutide (LIRA), a glucagon-like peptide-1 (GLP-1) analogue; [54] topical administration of bosentan (a dual endothelin receptor antagonist) reduced neurodegenerative changes in rodent models.[55]

Non-human primate models are limited with some success in showing early retinal neurodegenerative changes. [56,57]

Studies from donor eyes:
Increased GFAP in diabetic vs non-diabetic eyes: up-regulation of Bcl-2 and decreased expression of glutamine synthetase.

(Wolter 1961): "8 donor eyes of patients with DR.: proliferation of some nerves (the centrifugal nerves coming from the brain to the retinal blood vessels and atrophy of retinal neurons"

[58] article: 157 retinas from diabetic patients and 138 retinas from controls, noted vascular changes but also noted "loss of many of the neurons of IPL and GCLs"; loss of GCL is diffuse throughout the retina and quite variable. "In diabetes the retinal ganglion cells show all types of neuronal degeneration described for ganglion cells of the brain"..

"The NFL was significantly thinner (17.3 μm) in the eyes of six donors with DM than in the eyes of six similarly aged control donors (30.4 μm), although retinal capillary density did not differ in the two groups"[31]

Human donor eyes have shown over-expression of pro-apoptotic molecules in human diabetic retinal especially ganglion cells[59-61].

Indirect evidence from neurodegeneration in other areas:

Corneal neurodegeneration in the absence of vessel changes:
-132 eyes with T2D, 32 healthy controls -Nerve fiber density, nerve branch density and nerve fibre length were smaller in patients without DR, and worsened in patients with retinopathy.[62]
-19 patients with T1D (12 no DR, 7 with DR); significant reduction in corneal nerve branch (p=0.03), total nerve branch density (p=0.04), significantly decreased corneal nerve fibre density with DR (p=0.004).[63]

Corneal cellular and subbasal nerve

	-28 patients with T1D (18 with DR, 10without DR, matched healthy controls) underwent corneal confocal microscopy (CCM). Patients with T1D have lower epithelial and endothelial cell densities and higher keratocyte cell density compared to controls, but it is worse in people with T1D and Retinopathy.[64]<br><br>Cardiac autonomous neuropathy (CAN): not associated with microvascular changes. Per DCCT, CAN exists in 2.5-90% of patients with T1D, with or without diabetic retinopathy. CAN often manifests as lightheadedness, weakness, palpitations and syncope. Microvascular changes (retinopathy and albuminuria) are associated with progression of CAN.[65]<br><br>Peripheral neuropathy[66]- Changes in the peripheral nerves may not directly translate into changes in ocular nerves as peripheral nerves are myelinated with Schwann cells. Schwann cell dysfunction is thought to play a role in downstream damage to the peripheral nerves. it is thought that direct damage to Schwann cells from hyperglycemia or secondary to hyperglycemia induced endothelial dysfunction and consequent microvascular compromise, and result in nerve damage. While peripheral neuropathy clinical exam primarily focuses on assessment of nerve structure and function, microvascular changes are also thought to contribute to the nerve damage, suggesting the close link between the neural and vascular components in diabetes.[67]<br><br>GC-IPL thinning is associated with peripheral nerve conduction and autonomic nerve function[68].			
References for *in vivo* studies				
Are there clinical studies that address the relationship of this parameter to outcomes in DRD?	USER study: intravitreal fluocinolone implant was associated with decreased rate of loss of RNFL + GC/IPL complex in patients with persistent DME[69]	Quadrants of the retina with increased GCL loss were more likely to develop DR over the course of this 6 year study[28]	Retrospective cohort study showed that lower mGCIPL thickness plus the higher rate of decline in mGCIPL thickness, were associated with DR progression [32]	
If yes, which of the following clinical study types have been performed: systematic review, prospective randomized controlled trial, retrospective randomized controlled trial, cohort study, case/control study?	Multi-center retrospective review	Prospective study	Prospective	

If yes, please summarize the available evidence from clinical studies				
References for clinical studies				
Are there literature reviews that address the relationship of this parameter to outcomes in DRD?	Not with DRSS			
References for literature reviews				
Please give the Level of Evidence available from these combined studies (use Tables 1 and 2 below to determine Level of Evidence. For this purpose, please substitute "DRD parameter" for "tumor marker" or "marker" in Table 1)	2C	2C	2C	2C
**Statistical Considerations**				
What is the specific relationship of the parameter to clinical outcomes? Please specify effect sizes and measures of variability	No specific relationship established yet, prospective studies show change in thickness over time in GC-IPL and some studies show the decline in RNFL as well.			
What is the usefulness of the parameter or its thresholds for clinical or research decision making?	Difference between DM, non-DM and DM without DR have been identified in the literature and summarized in recent meta-analyses: see details above [29]			
Are there covariates that should be adjusted for when considering this parameter?	Age, cognitive impairment, glaucoma, ethnicity, axial length, refractive error: see factors affecting individual thickness measurements above			
Are there any additional statistical considerations for the use of this parameter?	Addressing all the correlated factors among eyes, multilevel models to account for clustering of thickness measures within eyes and correlation between eyes from same person			
**Gap Analysis**				
What are the gaps in the literature to prove or disprove the utility of this parameter?	1) Meaningful cutoff thresholds to determine presence of neurodegeneration<br>2) Functional implication (as assessed using e.g ERG/Reteval, contrast-sensitivity, visual field changes) for patients who have structural neurodegenerative changes.			

	3) Association between DRN and progression of vascular disease as established in the current DRSS<br>4) Implication of presence of DRN on diseases status as established by current DRSS e.g.<br>a) can treating DRN prevent vascular changes associated with DR or prevent progression of DR?<br>b) can treating it reduce the burden of treatments in DME?<br>c) Does DRN contribute to persistent DME, that is poorly responsive to current standard of care			
In your opinion, what clinical research study/studies could address these gaps?	**1) Design**: **Prospective study**<br>**Population:** All patients who have DM, no DR (as assessed on photo only? Wide-angle-photo? FA? OCT-A?),<br>**Study measures:** mRNFL and mGC-IPL thickness on SD-OCT; function (on ERG/Reteval/NEI-VFQ); DRSS at periodic intervals<br>**Outcomes:** a) factors associated with thickness measurements (2SD above or below normal using database of normal eyes) among patients with DM on SD-OCT; b) can neural degeneration as assessed on OCT predict risk of DME/PDR/progression of DR; development of persistent DME that is un-responsive to anti-VEGF; c) association between OCT measures and functional measures<br><br>**2) Design: Interventional study**<br>**Population:** DM patients with DRN as determined by definitions in Study # 1 and no DME or PDR<br>**Intervention:** Randomize to control vs treatment<br>**Outcomes:** can an intervention such as brimonidine or SST halt/slow down progressive neurodegenerative changes on OCT when compared to controls. Previously, EUROCONDOR has shown decreased progressive neurodysfunction among participants with DM and DR level≤35 who had some evidence of neurodysfunction at baseline. However, we do not know if preventing progressive neurodegeneration leads to fewer DR related complications in the future.			
Are there currently available datasets that could be used for these validation efforts?				
**Miscellaneous Questions**				
Is this parameter currently employed in clinical use?	Not for patients with DM	Not widely used	To evaluate glaucoma patients to some degree	Yes to evaluate glaucoma patients
Is assessment instrumentation needed to measure this parameter currently: available commercially, available but not FDA approved, not readily available, or not available?	Yes, available	Yes, available	Yes, available	Yes, available
What is the ease of implementation in the following environments: high resource academic center, high resource community practice, low	Easy, already in place	Easy, already in place	Easy, already in place	Easy, already in place

resource/underserved environment?				
What sites are appropriate for this assessment?<br>Indicate all relevant site types: retina clinic, general ophthalmology clinic, optometry clinic, endocrinology clinic, general medical clinic, patient home.	retina clinic, general ophthalmology clinic, optometry clinic,<br><br>potentially in endocrinology clinic if using a combined fundus photography and OCT camera<br>retina clinic, general ophthalmology clinic, optometry clinic,			
Is there any technology or advance either currently available, in development, or not yet developed that would make this parameter no longer important or relevant?<br>If yes, please specify what technology or advance	Not aware of it			
What unmet need in the staging of DRD does this parameter address?	Non-invasive structural assessment of neurodegenerative changes using an existing imaging device			
**Summary**				
Based on the above data, please provide an integrated evaluation regarding the overall importance of this parameter to the field currently. If not currently relevant, please summarize the potential for future relevance, necessary steps for validation and a reasonable time frame for this to occur.	Definitely has the potential to be a useful marker. Advantages include ease of use and its ready availability in the clinic. However, there is need for further consensus regarding which thickness measurement to use and follow over time. GC-IPL may be the most robust marker of neurodegeneration. Need a prospective study to establish which patients develop DRN, early data from prospective studies like EUROCONDOR suggest that not all patients develop DRN. Then design RCTs to test the impact of intervention on OCT markers of DRN and the functional consequences of untreated DRN.			
What is the duration to measure this parameter in the average patient (order of magnitude, i.e. 1min, 10min, 100min)	1 min			
Please comment on "patient friendliness" metrics (e.g. invasiveness, use of contrast dye,	Non-invasive and patient-friendly			

claustrophobia, distance to operator, ability to use given COVID-19 considerations)	

1. Liu X, Shen M, Huang S, Leng L, Zhu D, Lu F. Repeatability and reproducibility of eight macular intra-retinal layer thicknesses determined by an automated segmentation algorithm using two SD-OCT instruments. *PloS one.* 2014;9(2):e87996-e87996.
2. Lee K, Abràmoff MD, Sonka M, Garvin MK. Automated segmentation of intraretinal layers from spectral-domain macular OCT: reproducibility of layer thickness measurements. Paper presented at: Medical Imaging 2011: Biomedical Applications in Molecular, Structural, and Functional Imaging2011.
3. Francoz M, Fenolland JR, Giraud JM, et al. Reproducibility of macular ganglion cell-inner plexiform layer thickness measurement with cirrus HD-OCT in normal, hypertensive and glaucomatous eyes. *The British journal of ophthalmology.* 2014;98(3):322-328.
4. Carpineto P, Nubile M, Agnifili L, et al. Reproducibility and repeatability of Cirrus™ HD-OCT peripapillary retinal nerve fibre layer thickness measurements in young normal subjects. *Ophthalmologica Journal international d'ophtalmologie International journal of ophthalmology Zeitschrift fur Augenheilkunde.* 2012;227(3):139-145.
5. Mwanza JC, Durbin MK, Budenz DL, et al. Profile and predictors of normal ganglion cell-inner plexiform layer thickness measured with frequency-domain optical coherence tomography. *Investigative ophthalmology & visual science.* 2011;52(11):7872-7879.
6. Fletcher EL, Phipps JA, Ward MM, Puthussery T, Wilkinson-Berka JL. Neuronal and glial cell abnormality as predictors of progression of diabetic retinopathy. *Current pharmaceutical design.* 2007;13(26):2699-2712.
7. Lee VK, Hosking BM, Holeniewska J, et al. BTBR ob/ob mouse model of type 2 diabetes exhibits early loss of retinal function and retinal inflammation followed by late vascular changes. *Diabetologia.* 2018;61(11):2422-2432.
8. Vorwerk CK, Lipton SA, Zurakowski D, Hyman BT, Sabel BA, Dreyer EB. Chronic low-dose glutamate is toxic to retinal ganglion cells. Toxicity blocked by memantine. *Investigative ophthalmology & visual science.* 1996;37(8):1618-1624.
9. Lieth E, Barber AJ, Xu B, et al. Glial reactivity and impaired glutamate metabolism in short-term experimental diabetic retinopathy. Penn State Retina Research Group. *Diabetes.* 1998;47(5):815-820.
10. Lieth E, LaNoue KF, Antonetti DA, Ratz M. Diabetes reduces glutamate oxidation and glutamine synthesis in the retina. The Penn State Retina Research Group. *Exp Eye Res.* 2000;70(6):723-730.
11. Li Q, Puro DG. Diabetes-induced dysfunction of the glutamate transporter in retinal Müller cells. *Investigative ophthalmology & visual science.* 2002;43(9):3109-3116.
12. Barnstable CJ, Tombran-Tink J. Neuroprotective and antiangiogenic actions of PEDF in the eye: molecular targets and therapeutic potential. *Progress in retinal and eye research.* 2004;23(5):561-577.
13. Yoshida Y, Yamagishi S, Matsui T, et al. Protective role of pigment epithelium-derived factor (PEDF) in early phase of experimental diabetic retinopathy. *Diabetes Metab Res Rev.* 2009;25(7):678-686.
14. Shen X, Xie B, Cheng Y, Jiao Q, Zhong Y. Effect of pigment epithelium derived factor on the expression of glutamine synthetase in early phase of experimental diabetic retinopathy. *Ocul Immunol Inflamm.* 2011;19(4):246-254.
15. Liu Y, Leo LF, McGregor C, Grivitishvili A, Barnstable CJ, Tombran-Tink J. Pigment epithelium-derived factor (PEDF) peptide eye drops reduce inflammation, cell death and vascular leakage in diabetic retinopathy in Ins2(Akita) mice. *Mol Med.* 2012;18(1):1387-1401.
16. Seki M, Tanaka T, Nawa H, et al. Involvement of brain-derived neurotrophic factor in early retinal neuropathy of streptozotocin-induced diabetes in rats: therapeutic potential of brain-derived neurotrophic factor for dopaminergic amacrine cells. *Diabetes.* 2004;53(9):2412-2419.
17. Ali TK, Al-Gayyar MM, Matragoon S, et al. Diabetes-induced peroxynitrite impairs the balance of pro-nerve growth factor and nerve growth factor, and causes neurovascular injury. *Diabetologia.* 2011;54(3):657-668.
18. Yoshida Y, Yamagishi S-I, Matsui T, et al. Protective role of pigment epithelium-derived factor (PEDF) in early phase of experimental diabetic retinopathy. *Diabetes/Metabolism Research and Reviews.* 2009;25(7):678-686.
19. van Hagen M, Baarsma S, Mooy C, et al. Somatostatin and somatostatin receptors in retinal diseases. *European journal of endocrinology.* 2000.

20. Carrasco E, Hernández C, Miralles A, Huguet P, Farrés J, Simó R. Lower somatostatin expression is an early event in diabetic retinopathy and is associated with retinal neurodegeneration. *Diabetes care.* 2007;30(11):2902-2908.
21. Simó R, Carrasco E, Fonollosa A, García-Arumí J, Casamitjana R, Hernández C. Deficit of somatostatin in the vitreous fluid of patients with diabetic macular edema. *Diabetes Care.* 2007;30(3):725-727.
22. Carrasco E, Hernández C, Miralles A, Huguet P, Farrés J, Simó R. Lower Somatostatin Expression Is an Early Event in Diabetic Retinopathy and Is Associated With Retinal Neurodegeneration. *Diabetes Care.* 2007;30(11):2902.
23. Simó R, Lecube A, Sararols L, et al. Deficit of somatostatin-like immunoreactivity in the vitreous fluid of diabetic patients: possible role in the development of proliferative diabetic retinopathy. *Diabetes Care.* 2002;25(12):2282-2286.
24. Hernández C, Arroba AI, Bogdanov P, et al. Effect of Topical Administration of Somatostatin on Retinal Inflammation and Neurodegeneration in an Experimental Model of Diabetes. *J Clin Med.* 2020;9(8).
25. Wang J, Cui X, Roon P, Smith SB. Role of Sigma 1 Receptor in Retinal Degeneration of the Ins2Akita/+ Murine Model of Diabetic Retinopathy. *Investigative ophthalmology & visual science.* 2016;57(6):2770-2781.
26. Sato S, Hirooka K, Baba T, Tenkumo K, Nitta E, Shiraga F. Correlation between the ganglion cell-inner plexiform layer thickness measured with cirrus HD-OCT and macular visual field sensitivity measured with microperimetry. *Investigative ophthalmology & visual science.* 2013;54(4):3046-3051.
27. van Dijk HW, Verbraak FD, Stehouwer M, et al. Association of visual function and ganglion cell layer thickness in patients with diabetes mellitus type 1 and no or minimal diabetic retinopathy. *Vision research.* 2011;51(2):224-228.
28. van de Kreeke JA, Darma S, Chan Pin Yin J, et al. The spatial relation of diabetic retinal neurodegeneration with diabetic retinopathy. *PLoS One.* 2020;15(4):e0231552.
29. Tang Z, Chan MY, Leung WY, et al. Assessment of retinal neurodegeneration with spectral-domain optical coherence tomography: a systematic review and meta-analysis. *Eye (Lond).* 2020.
30. Pinilla I, Idoipe M, Perdices L, et al. CHANGES IN TOTAL AND INNER RETINAL THICKNESSES IN TYPE 1 DIABETES WITH NO RETINOPATHY AFTER 8 YEARS OF FOLLOW-UP. *Retina.* 2020;40(7):1379-1386.
31. Sohn EH, van Dijk HW, Jiao C, et al. Retinal neurodegeneration may precede microvascular changes characteristic of diabetic retinopathy in diabetes mellitus. *Proceedings of the National Academy of Sciences of the United States of America.* 2016;113(19):E2655-2664.
32. Kim K, Kim ES, Yu S-Y. Longitudinal relationship between retinal diabetic neurodegeneration and progression of diabetic retinopathy in patients with type 2 diabetes. *American Journal of Ophthalmology.* 2018;196:165-172.
33. Kim K, Kim ES, Kim DG, Yu SY. Progressive retinal neurodegeneration and microvascular change in diabetic retinopathy: longitudinal study using OCT angiography. *Acta Diabetol.* 2019;56(12):1275-1282.
34. Aschauer J, Pollreisz A, Karst S, et al. Longitudinal analysis of microvascular perfusion and neurodegenerative changes in early type 2 diabetic retinal disease. *British Journal of Ophthalmology.* 2020.
35. Lim HB, Shin YI, Lee MW, Koo H, Lee WH, Kim JY. Ganglion Cell–Inner Plexiform Layer Damage in Diabetic Patients: 3-Year Prospective, Longitudinal, Observational Study. *Scientific reports.* 2020;10(1):1-9.
36. Tavares Ferreira J, Proenca R, Alves M, et al. Retina and Choroid of Diabetic Patients Without Observed Retinal Vascular Changes: A Longitudinal Study. *Am J Ophthalmol.* 2017;176:15-25.
37. Lim HB, Shin YI, Lee MW, Park GS, Kim JY. Longitudinal Changes in the Peripapillary Retinal Nerve Fiber Layer Thickness of Patients With Type 2 Diabetes. *JAMA Ophthalmology.* 2019;137(10):1125-1132.
38. Simó R, Hernández C, Porta M, et al. Effects of topically administered neuroprotective drugs in early stages of diabetic retinopathy: results of the EUROCONDOR clinical trial. *Diabetes.* 2019;68(2):457-463.

39. Khawaja AP, Chan MPY, Garway-Heath DF, et al. Associations With Retinal Nerve Fiber Layer Measures in the EPIC-Norfolk Eye Study. *Investigative ophthalmology & visual science.* 2013;54(7):5028-5034.
40. Ko F, Muthy ZA, Gallacher J, et al. Association of Retinal Nerve Fiber Layer Thinning With Current and Future Cognitive Decline: A Study Using Optical Coherence Tomography. *JAMA neurology.* 2018.
41. Mwanza J-C, Durbin MK, Budenz DL, et al. Profile and Predictors of Normal Ganglion Cell–Inner Plexiform Layer Thickness Measured with Frequency-Domain Optical Coherence Tomography. *Investigative ophthalmology & visual science.* 2011;52(11):7872-7879.
42. Mauschitz MM, Bonnemaijer PWM, Diers K, et al. Systemic and Ocular Determinants of Peripapillary Retinal Nerve Fiber Layer Thickness Measurements in the European Eye Epidemiology (E3) Population. *Ophthalmology.* 2018;125(10):1526-1536.
43. Alves MR, Boia R, Campos EJ, et al. Subtle thinning of retinal layers without overt vascular and inflammatory alterations in a rat model of prediabetes. *Molecular vision.* 2018;24:353.
44. Hernández C, Bogdanov P, Corraliza L, et al. Topical administration of GLP-1 receptor agonists prevents retinal neurodegeneration in experimental diabetes. *Diabetes.* 2016;65(1):172-187.
45. Barber AJ, Lieth E, Khin SA, Antonetti DA, Buchanan AG, Gardner TW. Neural apoptosis in the retina during experimental and human diabetes. Early onset and effect of insulin. *The Journal of clinical investigation.* 1998;102(4):783-791.
46. Barber AJ, Antonetti DA, Kern TS, et al. The Ins2Akita mouse as a model of early retinal complications in diabetes. *Investigative ophthalmology & visual science.* 2005;46(6):2210-2218.
47. Bucolo C, Barbieri A, Viganò I, et al. Short-and Long-Term Expression of Vegf: A Temporal Regulation of a Key Factor in Diabetic Retinopathy. *Front Pharmacol.* 2021;12:707909.
48. Martin PM, Roon P, Van Ells TK, Ganapathy V, Smith SB. Death of Retinal Neurons in Streptozotocin-Induced Diabetic Mice. *Investigative ophthalmology & visual science.* 2004;45(9):3330-3336.
49. Sergeys J, Etienne I, Van Hove I, et al. Longitudinal In Vivo Characterization of the Streptozotocin-Induced Diabetic Mouse Model: Focus on Early Inner Retinal Responses. *Investigative ophthalmology & visual science.* 2019;60(2):807-822.
50. Yang JH, Kwak HW, Kim TG, Han J, Moon SW, Yu SY. Retinal Neurodegeneration in Type II Diabetic Otsuka Long-Evans Tokushima Fatty Rats. *Investigative ophthalmology & visual science.* 2013;54(6):3844-3851.
51. Zhi Z, Chao JR, Wietecha T, Hudkins KL, Alpers CE, Wang RK. Noninvasive Imaging of Retinal Morphology and Microvasculature in Obese Mice Using Optical Coherence Tomography and Optical Microangiography. *Investigative ophthalmology & visual science.* 2014;55(2):1024-1030.
52. Hammoum I, Benlarbi M, Dellaa A, et al. Retinal dysfunction parallels morphologic alterations and precede clinically detectable vascular alterations in Meriones shawi, a model of type 2 diabetes. *Exp Eye Res.* 2018;176:174-187.
53. Liu F, Saul AB, Pichavaram P, et al. Pharmacological Inhibition of Spermine Oxidase Reduces Neurodegeneration and Improves Retinal Function in Diabetic Mice. *Journal of clinical medicine.* 2020;9(2):340.
54. Liu J, Wei L, Wang Z, et al. Protective effect of Liraglutide on diabetic retinal neurodegeneration via inhibiting oxidative stress and endoplasmic reticulum stress. *Neurochem Int.* 2020;133:104624.
55. Bogdanov P, Simó-Servat O, Sampedro J, et al. Topical Administration of Bosentan Prevents Retinal Neurodegeneration in Experimental Diabetes. *International journal of molecular sciences.* 2018;19(11).
56. Robinson R, Barathi VA, Chaurasia SS, Wong TY, Kern TS. Update on animal models of diabetic retinopathy: from molecular approaches to mice and higher mammals. *Disease Models & Mechanisms.* 2012;5(4):444.
57. Xia Y, Luo Q, Chen J, et al. Retinal astrocytes and microglia activation in diabetic retinopathy rhesus monkey models. *Current eye research.* 2021.
58. Bloodworth JM, Jr. Diabetic retinopathy. *Diabetes.* 1962;11:1-22.

59. Podestà F, Romeo G, Liu W-H, et al. Bax is increased in the retina of diabetic subjects and is associated with pericyte apoptosis in vivo and in vitro. *The American journal of pathology.* 2000;156(3):1025-1032.
60. Oshitari T, Yamamoto S, Hata N, Roy S. Mitochondria-and caspase-dependent cell death pathway involved in neuronal degeneration in diabetic retinopathy. *British Journal of Ophthalmology.* 2008;92(4):552-556.
61. El-Asrar AMA, Dralands L, Missotten L, Al-Jadaan IA, Geboes K. Expression of apoptosis markers in the retinas of human subjects with diabetes. *Investigative ophthalmology & visual science.* 2004;45(8):2760-2766.
62. Bitirgen G, Ozkagnici A, Malik RA, Kerimoglu H. Corneal nerve fibre damage precedes diabetic retinopathy in patients with Type 2 diabetes mellitus. *Diabet Med.* 2014;31(4):431-438.
63. Deák EA, Szalai E, Tóth N, Malik RA, Berta A, Csutak A. Longitudinal Changes in Corneal Cell and Nerve Fiber Morphology in Young Patients with Type 1 Diabetes with and without Diabetic Retinopathy: A 2-Year Follow-up Study. *Investigative ophthalmology & visual science.* 2019;60(2):830-837.
64. Szalai E, Deák E, Módis L, Jr., et al. Early Corneal Cellular and Nerve Fiber Pathology in Young Patients With Type 1 Diabetes Mellitus Identified Using Corneal Confocal Microscopy. *Investigative ophthalmology & visual science.* 2016;57(3):853-858.
65. Witte DR, Tesfaye S, Chaturvedi N, Eaton SE, Kempler P, Fuller JH. Risk factors for cardiac autonomic neuropathy in type 1 diabetes mellitus. *Diabetologia.* 2005;48(1):164-171.
66. Lin IC, Wang YH, Lin CL, Chang YJ, Lee SH, Wang IJ. Diabetic polyneuropathy and the risk of developing diabetic retinopathy: a nationwide, population-based study. *Acta Ophthalmologica.* 2015;93(8):713-718.
67. Gonçalves NP, Vægter CB, Andersen H, Østergaard L, Calcutt NA, Jensen TS. Schwann cell interactions with axons and microvessels in diabetic neuropathy. *Nature reviews Neurology.* 2017;13(3):135-147.
68. Kim K, Kim ES, Rhee SY, Chon S, Woo J-t, Yu S-Y. Clinical characteristics and risk factors for retinal diabetic neurodegeneration in type 2 diabetes. *Acta Diabetologica.* 2017;54(11):993-999.
69. Lynch SK, Lee K, Chen Z, et al. Intravitreal Fluocinolone Acetonide May Decelerate Diabetic Retinal Neurodegeneration. *Investigative ophthalmology & visual science.* 2019;60(6):2134-2139.
