# Supplementary material for: A New Approach to Staging Diabetic Eye Disease: Staging of Diabetic Retinal Neurodegeneration and Diabetic Macular Edema
Source: Ophthalmol Sci. 2023 Oct 31;4(3):100420. doi: 10.1016/j.xops.2023.100420 (PMC10818256; doi:10.1016/j.xops.2023.100420)
Supplement: Table S7 [file mmc7.docx]

9-28-2023

To the editorial team Ophthalmology Science,

I agree to be listed in the acknowledgements section of the article “*A new approach to staging diabetic eye disease: staging of diabetic retinal neurodegeneration and diabetic macular edema*” to be published in the journal, *Ophthalmology Science*.

Electronically signed by

Patricia G. Duffel (Trish Duffel)
